# In vivo DNA damage protection during cell migration across confining embryonic tissue environments

**DOI:** 10.1038/s41556-026-02065-w

**Published:** 2026-10-09

**Authors:** Hanna-Maria Häkkinen, Soraya Villaseca, Zain Alhashem, Atrin Hamidzadeh, Szymon Chomiczewski, Maxime Desevedavy, Solene Leleux, You-Hsuan Liu, Julia Monika Becker, Mint R. Htun, Filomena Gallo, Dina El-Zohiry, Victor Petre, Kasia Stefanowski, Kristian Franze, Jörg Renkawitz, Elena Scarpa

**Affiliations:** 1 Department of Physiology, Development and Neuroscience, University of Cambridge, Cambridge, UK; 2 Medical Institute of Biophysics, Friedrich-Alexander-Universität Erlangen-Nürnberg, Erlangen, Germany; 3 Max-Planck-Zentrum für Physik und Medizin, Erlangen, Germany; 4 Randall Division for Cell and Molecular Biophysics, Guy’s Campus, London, UK; 5 Microscopy Bioscience Platform, University of Cambridge, Cambridge, UK; 6 Biomedical Center, Walter Brendel Center of Experimental Medicine, Institute of Cardiovascular Physiology and Pathophysiology, Klinikum der Universität, Ludwig-Maximilians-University Munich, Munich, Germany

**Keywords:** Cell invasion, Zebrafish, Nuclear envelope, Embryology, Double-strand DNA breaks

## Abstract

Cells migrating through confined spaces can experience nuclear deformation, nuclear envelope damage and DNA lesions, but the physiological consequences of confinement in vivo remain unclear. Using the zebrafish neural crest as a model of developmental migration, we show that tissue confinement increases along the antero–posterior axis and scales with the extent of nuclear deformation. Genetic and mechanical disruption of surrounding tissues relieves confinement and restores nuclear morphology in vivo. Surprisingly, even under extreme deformation, neural crest cells exhibit nucleo-cytoplasmic leakage without nuclear envelope rupture or increased DNA damage. Instead, confinement is associated with reduced LaminB2 at the nuclear envelope. Functional perturbation of LaminB2 reveals that its depletion accelerates recovery from deformation, whereas sustained expression causes persistent nuclear distortion, identifying LaminB2 as a regulator of nuclear deformability. RNA-seq of confined trunk neural crest cells reveals broad upregulation of DNA repair pathways, suggesting that these embryonic stem-like cells are intrinsically resistant to confinement-induced mechanical stress.

## Abstract (web summary)

Häkkinen et al. show that zebrafish neural crest cells encounter increased tissue-scale confinement along the body axis. Despite nuclear deformation, migrating neural crest cells experience neither nuclear envelope rupture nor increased DNA damage.

## Main

In vivo, migrating cells often navigate complex tissue environments, experiencing physical confinement by surrounding cells and tissues or by the extracellular matrix. The ability to negotiate passage through interstitial spaces is essential in physiology. For example, white blood cells patrol tissues or extravasate on demand upon injury or infection^1^. In disease, cancer cells often disseminate through confining tissue environments^2^, and the ability to survive physical confinement correlates with poor prognosis^3^.

Nuclear deformability represents a limiting factor for cells migrating through confinement^4^, as the nucleus is often the stiffest organelle of adult cells^5,6^. The nucleus is emerging as a key platform for cells to sense their surroundings. Cells respond to nuclear deformations by adapting their cytoskeleton organization^7,8^ and chemokine receptor profile^9^ to spatial constraints. However, confined cell migration through pores smaller than a critical 3-μm threshold^9^ can lead to compromised nuclear integrity due to transient ruptures of the nuclear envelope (NE)^10,11^ and to DNA damage accumulation^10–14^. This can drive a transition to invasive cancer phenotypes^14,15^ and tumour heterogeneity^12^. Nuclear deformation alone, in the absence of ruptures, can also induce DNA damage in cycling cells^16^. Thus, extreme spatial constraints to cell migration can have deleterious outcomes for cells. However, the consequences of confinement on physiological cell migration remain largely unexplored. Recent in vivo work in *Drosophila* immune-cell migration highlights increased endogenous DNA damage as immune cells move through highly confining haemolymph vessels^17^. Mouse primordial germ cells and mouse cerebellar granule neurons, which navigate in vivo through dense tissues during their developmental cell migration, also suffer striking cell and nuclear deformations, accumulating substantial DNA damage^18,19^. However, most immune cells and neurons are terminally differentiated^19,20^, and germ cells, being totipotent, are subject to a strict quality control and elimination of unfit cells^21^. Whether multipotent cells, essential for embryonic development, can adapt to physical stress in an in vivo physiological tissue context has not yet been addressed.

To tackle this question we used the zebrafish neural crest, harnessing the natural variation in the neural crest microenvironment to interrogate how migratory cells cope with tissue confinement in vivo. Neural crest cells (NCs) are a pluripotent stem-cell population, forming at the dorsal aspect of the neural tube from head to tail of vertebrate embryos^22^. NCs are highly invasive, and migrate long distances across tissues, eventually differentiating into multiple cell types. Across vertebrates, cranial neural crest cells (cNCs) migrate under the ectoderm through interstitial spaces filled with loosely organized extracellular matrix^23–25^. In contrast, trunk neural crest cells (tNCs) migrate in dense tissue environments, invading narrow inter-tissue spaces between the spinal cord and the somites using a highly conserved migratory path^26–30^. In this Article we show that NCs encounter a fine-grained gradient of in vivo tissue-scale confinement along the antero–posterior (AP) axis of the embryo, which results in dramatic nucleus deformations. Using quantitative in vivo imaging, as well as in vivo genetic and mechanical manipulations, we find that nucleus shape changes are caused by confinement imposed by the surrounding somite tissue. We uncover that NCs do not experience NE ruptures nor increased DNA damage. Instead, they adapt to confinement through the regulation of LaminB2, enabling efficient nuclear deformation and recovery cycles. Although LaminB2 regulation facilitates nuclear shape changes, a photoconversion and RNA-seq approach suggests that tNCs maintain low endogenous DNA damage when migrating in a confined in vivo tissue environment, probably by upregulating a complex DNA damage response program.

## Results

### Neural crest nucleus shape behaviours differ across the AP axis of the embryo

**Fig. 1:**
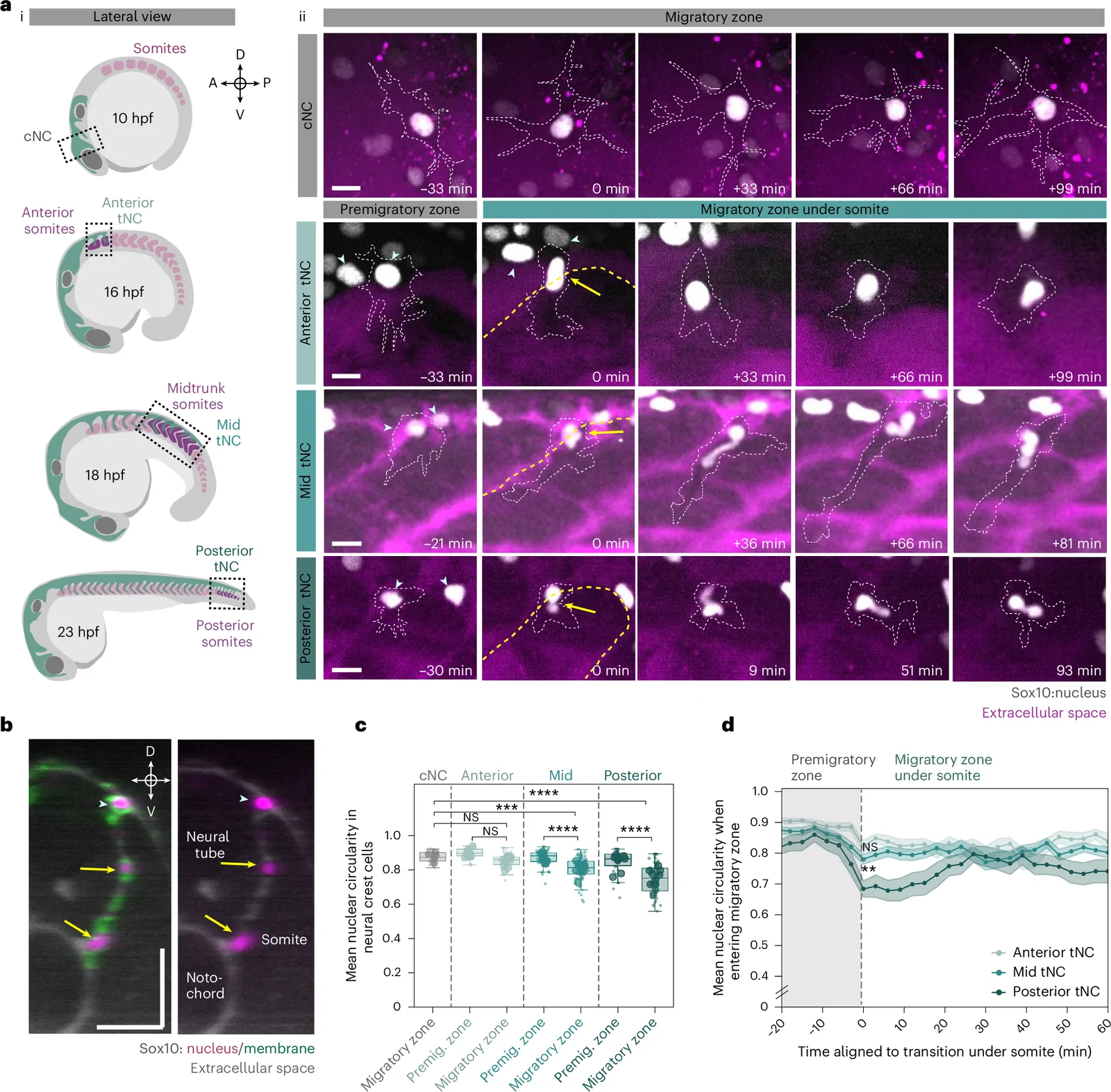
Neural crest nuclear shape dynamics differ along the AP axis of the embryo. **a**, (i) Sketches of zebrafish embryos at the developmental stages corresponding to NC migration onset (cranial 10 hours post fertilization (hpf), anterior trunk 16 hpf, mid trunk 18 hpf, posterior 23 hpf). (ii) Representative live-imaging time series of the maximum projection of single NCs expressing a tissue-specific nuclear marker during the transition from the premigratory zone to the migratory zone. Yellow arrows indicate the nuclear shape upon the migratory transition. Light blue arrowheads indicate the nuclear shape in the premigratory region. White dotted lines indicate the cell membrane. Yellow dashed lines indicate the somite boundary. The extracellular space is labelled with dextran (magenta). **b**, Representative resliced cross-section of a live embryo (line width 10 pixels) at the mid trunk region during neural crest migration. Yellow arrows indicate nuclei in the migratory region. Light blue arrowheads indicate nuclei in the premigratory region. **c**, Mean nuclear circularity per nucleus in NCs in the premigratory (premig.) region and migratory region along the AP body axis. cNCs *n* = 25 cells, five embryos; anterior, premigratory *n* = 19 cells, migratory *n* = 23 cells, nine embryos; mid trunk, premigratory *n* = 45 cells, migratory *n* = 72 cells, 11 embryos; posterior, premigratory *n* = 26 cells, migratory *n* = 51 cells, 10 embryos. Kruskal–Wallis Dunn’s multiple comparison test, ***P = 0.0001 (cNC versus mid trunk mig), ****P < 0.0001. Small dots represent the mean circularity value of a single nucleus over time; large dots represent mean values per embryo. The box plots show the median, the first and third quartiles, and 1.5× interquartile range. **d**, Mean nuclear circularity aligned to the transition from the premigratory region to the migratory region under the somite. Anterior *n* = 19 cells, nine embryos; mid trunk *n* = 40 cells, 11 embryos; posterior *n* = 23 cells, nine embryos. Data are presented as mean and s.e.m. Tukey’s multiple comparisons test at 0-min timepoint; *P* = 0.3578 (NS), **P = 0.0029. For each timepoint at least 17 nuclei are included. The dashed line separates the premigratory (grey) and migratory regions. The *y* axis is cut to improve visualization. All statistical tests are two-sided. A, anterior; P, posterior; D, dorsal; V, ventral. Scale bars, 10 µm (**a**(ii)), 40 µm (**b**). NS, not significant. Source data

**Extended Data Fig. 1:**
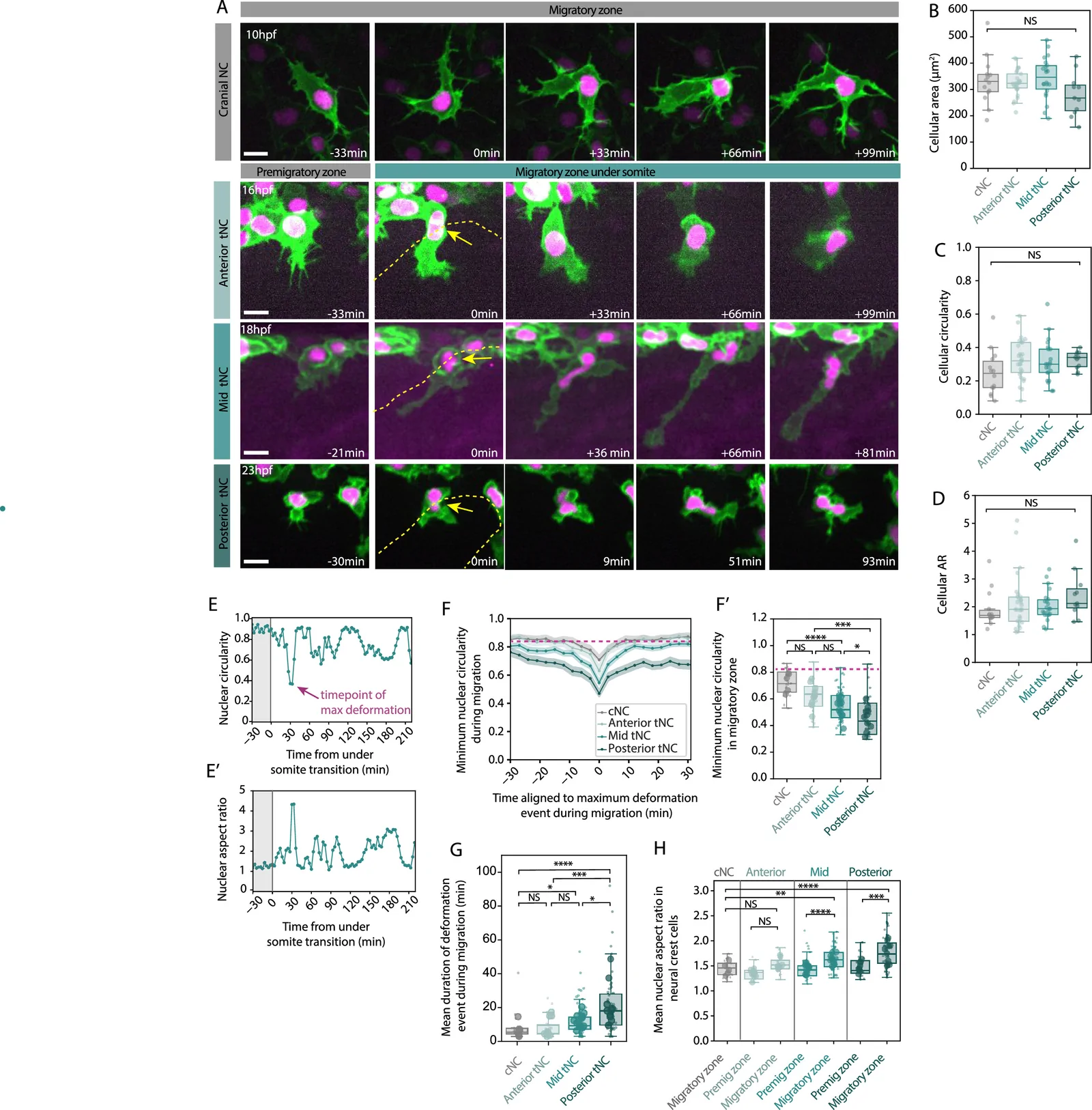
Neural crest nucleus and cell shape behaviours across the AP axis of the embryo. **A)** Representative time series of single neural crest cells nuclei and membranes during the transition from premigratory zone to the migratory zone in live embryos. Yellow arrowheads indicate nuclear shape upon the migratory transition. Yellow dashed line indicates the location of the somite boundary. **B)** Cytoplasmic area in migratory NC cells, cNC n = 14 cells, 4 embryos, Anterior n = 29 cells, 14 embryos, Mid trunk n = 22 cells, 9 embryos, Posterior n = 11 cells, 9 embryos. One-way ANOVA multiple comparison test, p = 0.1376. Each dot represents a cell. **C)** Cell shape circularity: cNC n = 14 cells, 4 embryos; Anterior n = 29 cells, 14 embryos; Mid trunk n = 22 cells, 9 embryos; Posterior n = 11 cells, 9 embryos. One-way ANOVA multiple comparison test, p = 0.2009. Each dot represents a cell. **D)** Cell shape Aspect Ratio: cNC n = 14 cells,4 embryos; Anterior n = 29 cells, 14 embryos; Mid trunk n = 22 cells, 9 embryos; Posterior n = 11 cells, 9 embryos. Kruskal-Wallis Dunn’s multiple comparison test, p = 0.2624. Each dot represents a cell. **E-E’)** Example of nuclear circularity (E) and AR (E’) over time in a single mid trunk neural crest cell aligned to the transitioning from premigratory region to the migratory region under the somite. Grey area indicates the premigratory region. **F)** Mean nuclear circularity aligned to the minimum circularity event during NC migration at different anterior-posterior regions. Mean ± SEM. At each timepoint at least 16 nuclei were included. **F’)** comparison of minimum nuclear circularity in migratory cells. cNC n = 23 cells, 5 embryos; anterior n = 21 cells, 9 embryos; mid trunk n = 67, 11 embryos; posterior n = 49 cells, 10 embryos. Kruskal-Wallis Dunn’s multiple comparison test p = 0.0273 (*), p = 0.0002 (***), p < 0.0001 (****). Each dot represents the minimum circularity value of a single nucleus, large dots represent mean value per embryo. Mean ± SEM. Pink line showing mean value of circularity in all NC populations as threshold for nuclear deformation. **G)** Mean duration of nuclear deformation events per nucleus. cNC n = 17 cells, 5 embryos; Anterior n = 17 cells, 9 embryos; Midtrunk n = 67 cells, 11 embryos; Posterior n = 47 cells,10 embryos. Kruskal-Wallis Dunn’s multiple comparison test p = 0.0244 (CNC vs Midtrunk), p = 0.0169 (Midtrunk vs Posterior), p = 0.0002 (***), p < 0.0001 (****). Each dot represents a mean duration of deformations per nucleus, large dots represents mean value per embryo. **H)** Mean nuclear aspect ratio per nucleus in neural crest cells in premigratory region and migratory region along the anterior-posterior body axis. cNC n = 25 cells, 5 embryos; anterior: n = 19 premigratory cells, n = 23 migratory cells, 9 embryos; midtrunk: n = 45 premigratory cells, n = 72 migratory cells, 11 embryos; posterior: n = 26 premigratory cells, n = 51 migratory cells, 10 embryos. Kruskal-Wallis Dunn’s multiple comparison test p = 0.0072 (**), p = 0.0003 (***), p < 0.0001 (****). Each dot represents mean AR of a single nucleus over time, large dots represents mean value per embryo. Box plots show the median, the 1st and 3rd quartile and the 1.5x inter-quartile range. All statistical tests two-sided. Scale bar 10 µm (A). Source data

To investigate how cells navigate through physically changing tissue environments in vivo, we used the zebrafish neural crest as a model system. The zebrafish neural crest migrates across a variety of tissue contexts along the AP axis of the embryo^31,32^. Cells navigating complex tissue environments deform the nucleus to overcome physical obstacles or to move through dense extracellular matrix^4^. We asked whether nuclear deformation also occurs in a physiological context during in vivo NC migration. We compared cNCs, anterior trunk neural crest (atNC) of the vagal streams (somites 1 and 2), tNC (somites 7–12) and posterior trunk neural crest (posttNC, somites 27–33; Fig. 1a(i)). In zebrafish, cNCs form lateral to the neural keel, from where they disperse into a loose collective of mesenchymal cells^23,31^. tNCs, on the other hand, form a premigratory cohort spatially restricted between the embryonic epithelium (enveloping layer (EVL)) and the neural tube. From here, they migrate collectively as a chain of cells^33^ between surrounding trunk tissues (Fig. 1b). To monitor nuclear shape changes upon in vivo migration, we carried out in vivo live imaging of embryos expressing the sox10:mG tissue-specific reporter^33^, which allows the visualization of NC and nucleus shapes (Extended Data Fig. 1a). To visualize the surrounding tissue context, we injected fluorescently labelled dextran^34^. We observed that although cNC and atNC nuclei maintain a circular, isotropic shape during migration, mid tNC and posttNC dramatically deform their nuclei (Fig. 1a(ii) and Supplementary Videos 1–4). By segmenting the nuclear shapes and measuring nucleus circularity and aspect ratio over time, we found that each cell experiences multiple deformation events during its migration (Extended Data Fig. 1e,e′). Because nuclear shape changes occur at different timepoints for every cell, to quantitatively compare nuclear morphometrics over time across the cNC, atNC, mid tNC and posttNC populations we identified the maximum deformation event per each cell track as the timepoint of minimum circularity (Extended Data Fig. 1e, arrow, and 1f) and we found that nuclear shape changes became increasingly stronger along the AP axis of the embryo (Extended Data Fig. 1f’). Similarly, we found that the duration of each deformation event also increases along the AP axis of the embryo (Extended Data Fig. 1g). We observed that, although premigratory tNC nuclei appeared relatively circular in shape across the tNC population (Fig. 1a(ii), light blue arrowheads, and Fig. 1c), nuclear deformation started as they moved to the migratory zone, squeezing under the somite (Fig. 1a(ii), yellow arrows, dotted lines). Using the dextran labelling to align nuclear shape tracks to the timepoint when tNCs enter the interstitial space underneath the somite, we observed that mid tNC and posttNC nuclei deformed notably when migrating under the somite (Fig. 1a(ii), yellow arrows, and 1c,d). In contrast, atNC nuclei did not change shape significantly as they moved under the somite, and their nuclear shape metrics remained similar to cNCs (Fig. 1c and Extended Data Fig. 1d). Nuclear deformation scales along the AP axis of the embryo—we detected markedly lower nuclear circularities and higher nuclear aspect ratios in mid and posttNC (Fig. 1c,d and Extended Data Fig. 1f,f′,h). In contrast, cell shape metrics did not change notably across neural crest subpopulations (Extended Data Fig. 1a–d). In summary, we show that NCs display striking differences in nuclear deformation dynamics across the embryo.

### Tissue-scale confinement differs across the AP axis of the embryo

**Fig. 2:**
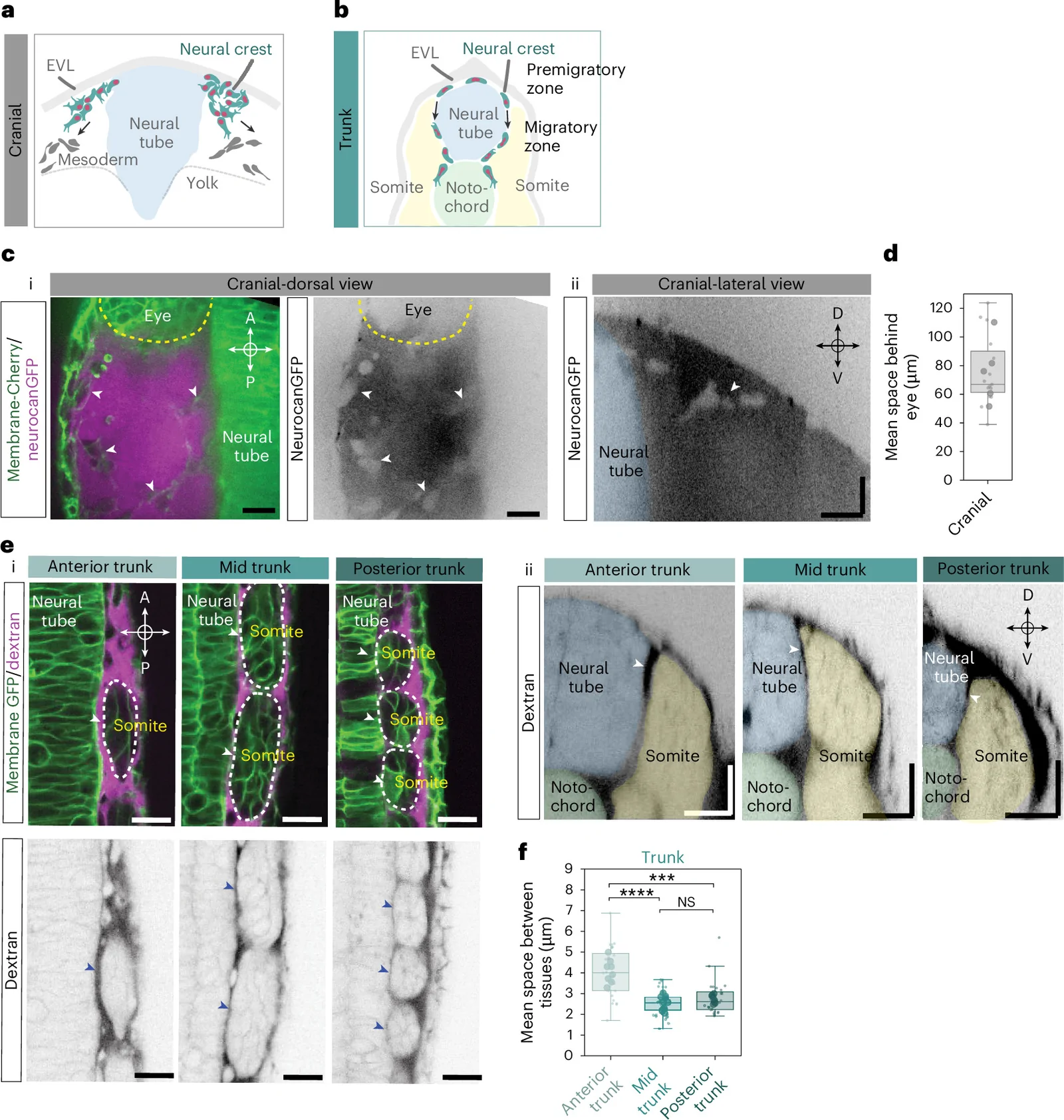
Tissue-scale confinement differs across the AP axis of the embryo. **a**,**b**, Sketch of cross-sections of the cranial region (**a**) and trunk region (**b**) in a zebrafish embryo during neural crest migration. Arrows indicate the direction of neural crest migration. **c**, (i) Representative live embryo dorsal view and (ii) resliced cross-section of the cranial region, showing the extracellular space between membrane labelled tissues along the migratory path and the migrating NCs (white arrowheads). Yellow dashed lines indicate the location of the eye. **d**, Mean space between the EVL and the neural tube behind the eye 10 μm along the migratory path of cNCs. *n* = 15 measurements, three measurements per embryo, five embryos. Small dots indicate the mean width of the extracellular space along the migratory path; large dots indicate mean values per embryo. **e**, (i) Representative dorsal view and (ii) resliced cross-section of the anterior trunk, mid trunk and posterior trunk at the onset of neural crest migration showing the extracellular space between tissues along the migratory path of the neural crest (white arrowheads, top; blue arrowheads, bottom) in live embryo. White dashed lines indicate somites. Pseudo colours indicate adjacent tissues. **f**, Mean space between somites and the neural tube at different anterior–posterior regions along the first 10 μm along the neural crest migratory path. Anterior trunk *n* = 28 somites, eight embryos; mid trunk *n* = 44 somites, nine embryos; posterior trunk *n* = 28 somites, eight embryos. Kruskal–Wallis Dunn’s multiple comparison test, ***P = 0.0002, ****P < 0.0001. Small dots indicate mean width of the extracellular space along the dorsal path per somite; large dots indicate mean values per embryo. The box plots show the median, the first and third quartiles and the 1.5× interquartile range. All statistical tests are two-sided. Scale bars, 20 μm. Source data

**Extended Data Fig. 2:**
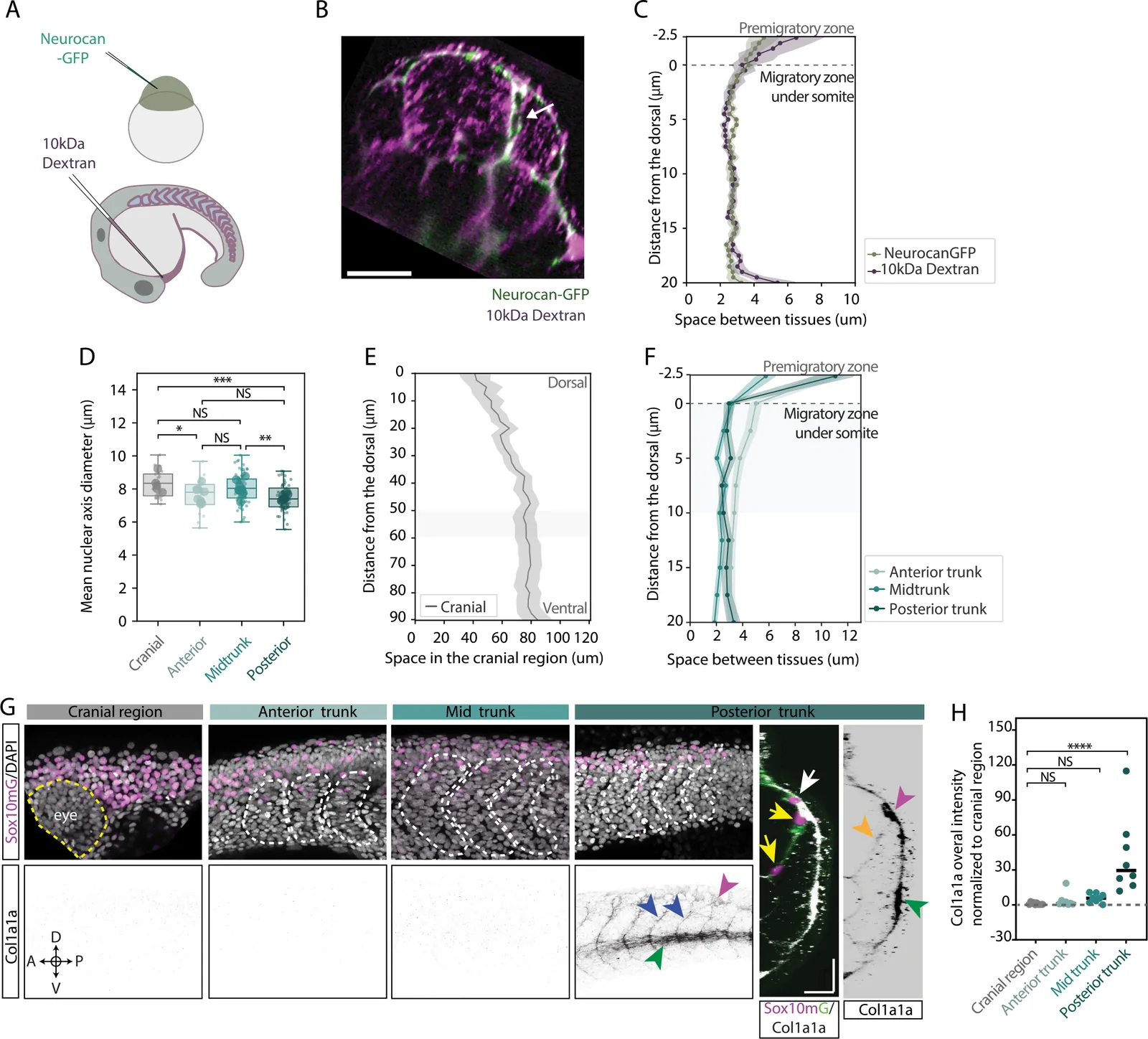
Extracellular space controls and extracellular matrix measurements across the AP axis of the embryo. **A)** Sketch of mRNA injection of Neurocan-GFP at 1-cell stage and Dextran injection prior to imaging. **B)** Representative dynamic cross-section showing the extracellular space between adjacent tissues using dextran and Neurocan-GFP in live embryo. White arrow highlight the migratory path. **C)** Mean width of extracellular space between somite and neural tube using alternative labelling methods for extracellular space, Neurocan-GFP and Dextran, in mid trunk region. n = 4 somites, 2 embryos. Mean and SEM presented. **D)** Mean nuclear axis diameter in neural crest cell at different anterior-posterior region. cNC n = 25 cells, 5 embryos; anterior n = 23 cells, 9 embryos; mid trunk n = 77 cells, 11 embryos; posterior n = 53 cells, 10 embryos. Kruskal-Wallis Dunn’s multiple comparison test p = 0.0399 (*), p = 0.0013 (**), p = 0.0004 (***). Small dots: mean nuclear axis diameter per nucleus over time, large dots: mean value per embryo. Box plots show the median, the 1st and 3rd quartile and the 1.5x inter-quartile range. **E)** Mean width of extracellular space along the migratory path of neural crest in cranial region from dorsal to ventral behind the eye. Shaded area indicates the region included to the panel Fig. 2D. n = 15 measurements, 3 measurements per embryo, 5 embryos. Mean and SEM presented. **F)** Mean width of extracellular space in along the migratory path of neural crest in anterior, mid and posterior trunk regions, aligned to the initiation of migratory path. Shaded area indicates the region included in the analysis in panel Fig. 2F. Anterior n = 28 somites,8 embryos; mid trunk n = 44 somites, 9 embryos; posterior n = 28 somites, 8 embryos. Mean ± SEM. **G)** Representative images of collagen1a1a immunostaining across the embryo AP axis at the timepoint corresponding to NC migration for each region. Maximum projections (between 45 to 95 slices with 0.5μm z-step): neural crest and surrounding tissues (upper panel) and localization of collagen1a1a at these regions (lower panel). Blue arrowheads highlight collagen1a1a at the somite boundaries, green arrowheads: collagen1a1a at the lateral line. Cross-section of the posterior trunk shows the localization of neural crest cells and collagen1a1a at the premigratory region (white arrow and purple arrowhead) and in the migratory path (yellow arrows and orange arrowhead). Yellow dashed line indicates the location of an eye in cranial regions. White dashed lines indicate the somite boundaries. **H)** Normalised col1a1a at cranial (n = 10 embryos), anterior trunk (n = 8 embryos), mid trunk (n = 8 embryos) and posterior trunk (n = 8 embryos) region at the timepoint corresponding to NC migration at each region normalised to cranial region. Kruskal-Wallis Dunn’s multiple comparison test between cranial to every other region. p < 0.0001. All statistical tests two-sided. A=anterior, P=posterior, D=dorsal, V=ventral. Scale bars 40 μm (B, G; lateral view), 20 μm (G; cross-section). Source data

We reasoned that the different nuclear shape behaviours we observe might be caused by differences in the size and matrix composition of the inter-tissue spaces encountered by the migrating neural crest. Indeed, although cranial neural crest migrates lateral to the neural tube into sparsely organized head mesoderm^23^ (Fig. 2a), trunk neural crest migrates confined between closely apposed surrounding tissues (Fig. 2b) lined with fibronectin and laminin^35,36^. To quantitatively measure the size of the interstitial spaces, we visualized them either with injected 10-kDa fluorescent dextran or with fluorescently tagged secreted Neurocan-GFP messenger RNA (mRNA), which binds to hyaluronic acid^37^ (Extended Data Fig. 2a). Measurement of interstitial spaces in embryos co-injected with 10-kDa dextran and Neurocan-GFP showed near-equivalent values (Extended Data Fig. 2b,c), so these approaches were used in parallel. cNCs migrate into a fluid-filled cavity posterior to the eye (Fig. 2c(i,ii)). By comparing the diameter of the neural crest nucleus (as an estimation of its size) (Extended Data Fig. 2d) to the width of the inter-tissue space across the mediolateral axis of the embryo (Fig. 2c,d and Extended Data Fig. 2e), we observed that the cavity is wide relative to the size of individual cNCs (Fig. 2c(i,ii), arrowheads). Hence, we concluded that cNCs migrate in a non-confined tissue environment. Next, we investigated the space along the migratory path in the trunk. In vivo imaging of 10-kDa dextran revealed that the spaces along the tNC migratory path, between the neural tube, somite and notochord, were extremely narrow (Fig. 2b,e(i,ii)). Because we observed that tNC nuclear deformation consistently initiated as tNC nuclei squeezed under the somite (Fig. 1c,d), we measured the mediolateral width of the inter-tissue space at the entry of the neural tube/somite interface (Fig. 2f and Extended Data Fig. 2f). We found anterior trunk inter-tissue spaces (Fig. 2f) to be slightly smaller than the mean atNC nuclear diameter (Extended Data Fig. 2d), while mid trunk and posterior trunk inter-tissue spaces were notably smaller than the mid tNC and posttNC nuclear diameter (Fig. 2f and Extended Data Fig. 2f), suggesting that mid tNC and posttNC nuclei were under constriction during migration. We also found that Neurocan-GFP localized to the inter-tissue spaces in both cranial and trunk regions, supporting that inter-tissue spaces were filled with hyaluronic acid across the AP-body axis (Extended Data Fig. 2b and Fig. 2c(i,ii)). As networks of fibrillar collagen constitute an obstacle for the nuclei of migrating cells in three dimensions^4^, we next asked whether collagens are also involved in nuclear deformations in NC migration. Most collagens are not yet expressed in early zebrafish embryos; however, Col1a1a has previously been linked to tNC migration^38^. Immunostaining of Col1a1a revealed notable immunoreactivity of Col1a1a only in the posterior trunk at the time of NC migration, while cranial, anterior and mid-trunk tissues were similarly devoid of type 1 collagen before migration onset (Extended Data Fig. 2g,h). Although Col1a1a does not show localization at the migratory path of the posterior tNC, the presence of Col1a1a in the premigratory region of the posterior trunk (Extended Data Fig. 2g) might explain the stronger nuclear shape changes we detected in the premigratory population in posttNC (Fig. 1c,d). In summary, we find that, along the zebrafish trunk, confinement imposed by the surrounding tissues and by the interstitial extracellular matrix scales with nuclear deformation across migrating neural crest subpopulations.

### In vivo perturbations of tissue-scale confinement restore nucleus circularity

**Fig. 3:**
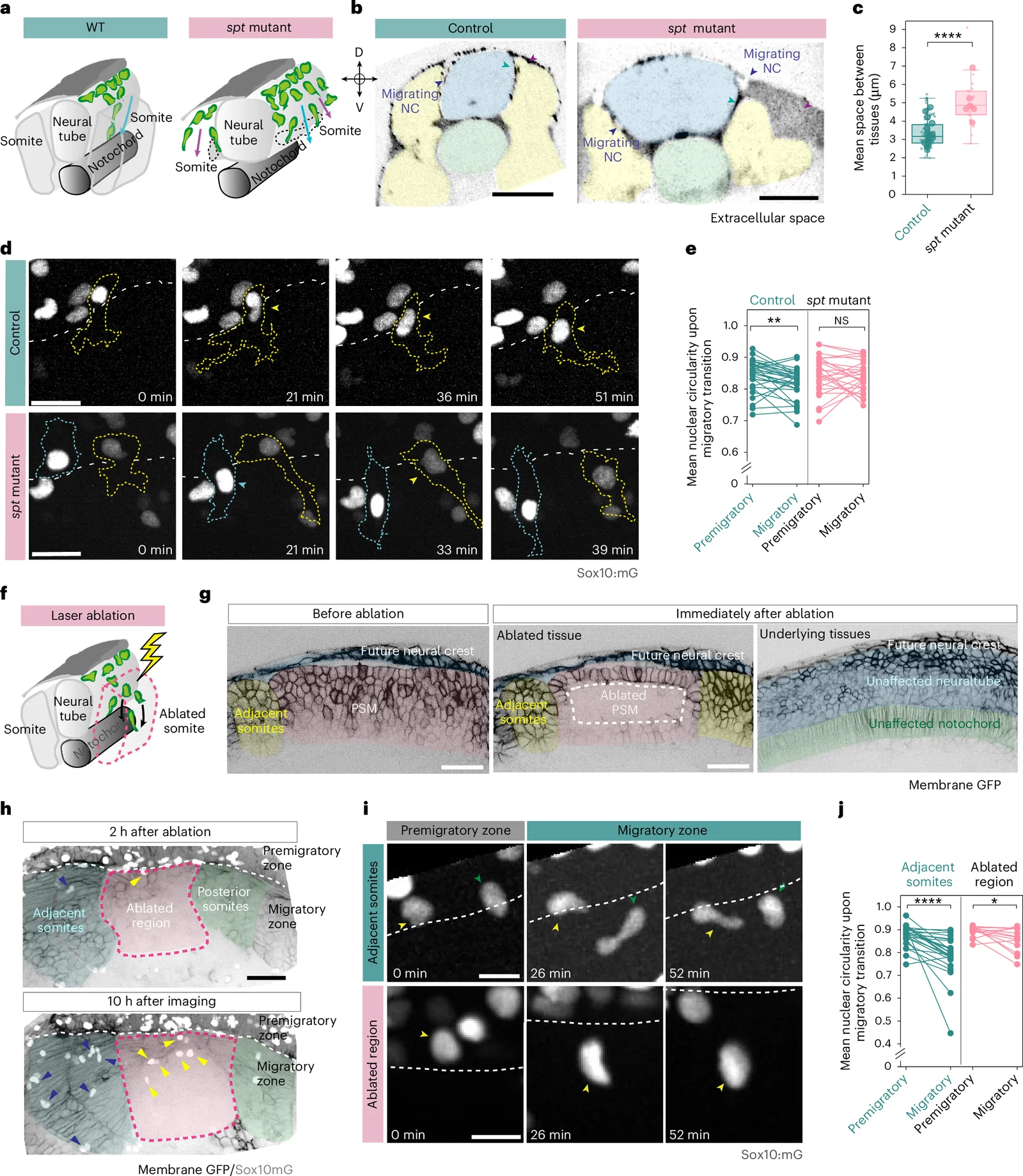
In vivo perturbations of tissue-scale confinement restore nuclear circularity. **a**, Sketches of tissue organization in zebrafish mid trunk in wild-type (WT) and spadetail mutant (*spt*) fish. Blue arrows indicate neural crest migration through the dorsal path between the somite and neural tube; the purple arrow indicates the lateral path between the somite and EVL. The dashed line shows the reduced somite size in the mutant line. **b**, Representative resliced cross-sections of a mid trunk region in control and *spt* mutant embryos showing the extracellular space between tissues along the neural crest migratory path (green arrowheads) in live embryos. The blue arrowheads indicate the migrating neural crest cells. The purple arrowhead highlights the space along the lateral migratory path between the EVL and somite. Pseudo colours facilitate the visualization of adjacent tissues. **c**, Mean width of the extracellular space along the dorsal path in control and *spt* mutant embryos. Control *n* = 80 somites, 16 embryos; *spt* mutant *n* = 23 somites, eight embryos. Mann–Whitney test, ****P < 0.0001. The box plots show the median, the first and third quartiles and the 1.5× interquartile range. Each dot represents the mean width of the extracellular space along the dorsal path per somite; large dots represent mean values per embryo. **d**, Representative time series of NCs during the transition from the premigratory zone to the migratory zone in control and *spt* mutant embryos. Arrowheads indicate the nuclear shape during the migratory transition and colours indicate specific cell. Yellow and blue dashed lines show the locations of cell membranes during migration. White dashed lines show the location of the somite boundary in trunk regions. **e**, Mean nuclear circularity before and after transitioning to the migratory zone. Control *n* = 30 cells, three embryos; *spt* mutant *n* = 26 cells, four embryos. Wilcoxon matched-pairs signed rank test: **P = 0.0030. **f**, Diagram of the laser ablation approach to remove an individual somite. Black arrows show the direction of neural crest migration. The dashed line outlines the ablated somite. **g**, Representative images of membrane GFP-labelled presomitic mesoderm (PSM) and intact surrounding tissues before and immediately after laser ablation. Pseudo colours indicate the locations of surrounding tissues. The dashed line outlines the ablated region. **h**, Representative images of tissue organization and NC location at the onset of migration (top) and 10 h after imaging (bottom) in a laser-ablated embryo 5 h after ablation. Global membrane staining visualizes the location of intact somites. Purple arrowheads highlight the neural crest migrating in intact adjacent regions, and yellow arrowheads highlight neural crest migrating into the ablated region. The white dashed lines separate the premigratory zone from the migratory zone. Pseudo colours indicate the location of the intact and ablated tissues. The maximum projection of the neural crest channel is overlaid with a single plane of membrane GFP channel for improved visualization. **i**, Representative time series of the maximum projection of NCs during the transition from the premigratory zone to the migratory zone in intact adjacent regions and ablated regions of the embryo. Arrowheads indicate the nuclear shape at the migratory transition. White dashed lines separate the premigratory region from the migratory region. **j**, Mean nuclear circularity before and after transitioning to the migratory zone at intact adjacent regions and ablated regions of the embryo. Adjacent region *n* = 25 cells and ablated region *n* = 14 cells, five embryos. Wilcoxon matched-pairs signed rank test: *P < 0.0001 (adjacent), ****P = 0.0419 (ablated). Cohen’s *d* effect size, 0.8886 (between migratory populations). All statistical tests were two-sided. Scale bars, 40 μm (**b**, **g** and **h**), 20 μm (**d**), 10 μm (**i**). Source data

**Extended Data Fig. 3:**
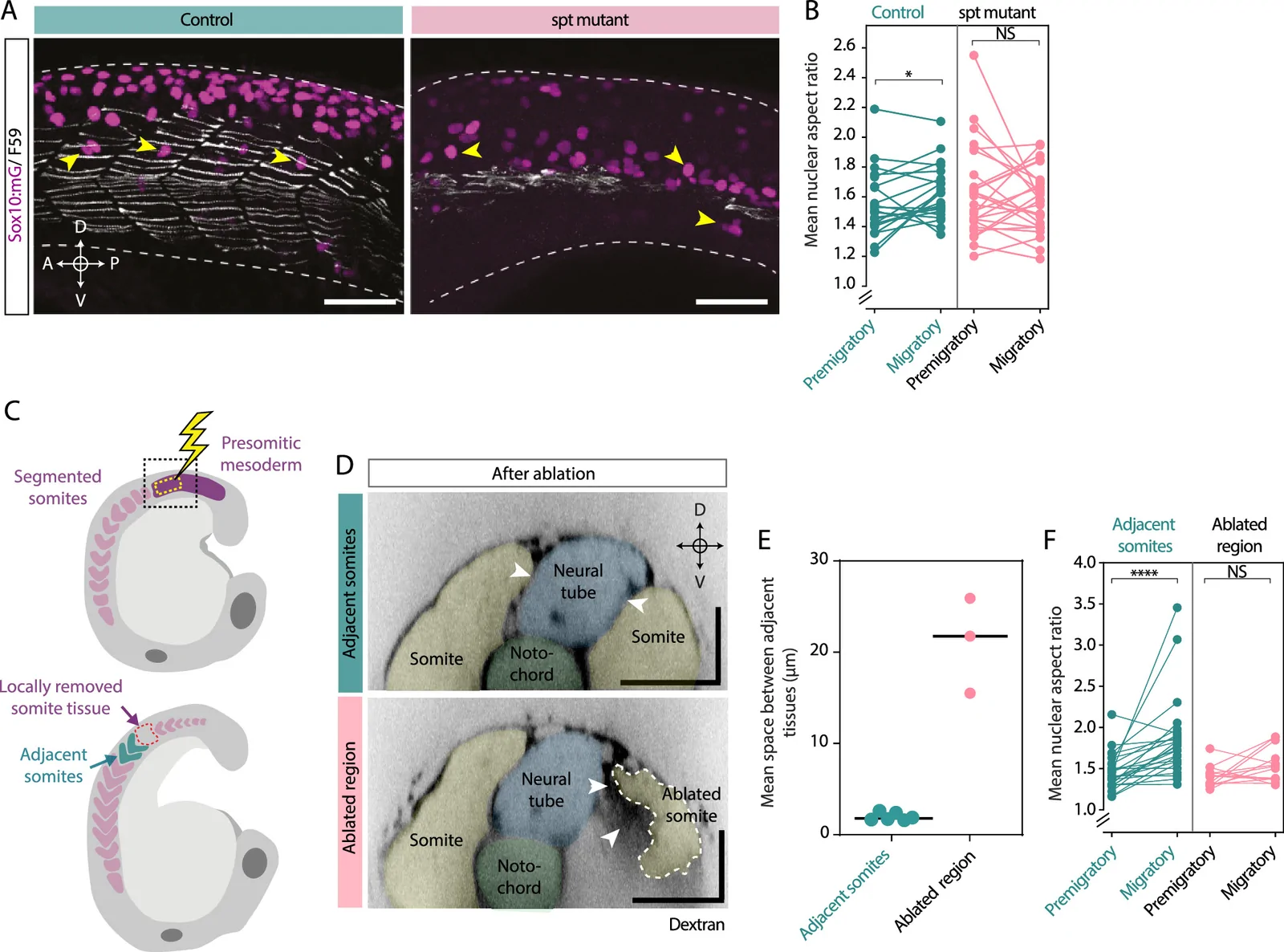
In vivo perturbations of tissue-scale confinement restore nuclear aspect ratio. **A)** Representative images of fixed control and spt mutant embryos at mid trunk region. Maximum projections (n = 30 slices, 1 μm z- step) shows the migrating neural crest cells (yellow arrowheads) and localization of myosin heavy chain (F59) in somite tissue. **B)** Mean nuclear aspect ratio before and after transitioning to the migratory zone. Control n = 22 cells from 2 embryos; spt mutant n = 26 cells from 4 embryos. Wilcoxon matched-pairs signed rank test, p = 0.0462. Each dot represents a mean value of single nucleus in given region. Y-scale cut to improve visualisation. **C)** Sketch showing the targeted region witing the embryo during and after laser ablation. **D)** Representative images from cross-sections showing the extracellular space in laser ablated embryos in ablated region and intact somite region. Dashed lines highlights the ablated somites. Pseudo colours indicate the location of adjacent tissues. White arrowheads point the neural crest migratory region. **E)** Mean space between adjacent tissues along 30um from the initiation of the dorsal migratory path at intact somite regions and at ablated somite region. Adjacent somites n = 6 from 3 embryos, ablated region n = 3 from 3 embryos. Mann-Whitney test, p = 0.0238. **F)** Mean nuclear aspect ratio before and after transitioning to the migratory zone at intact adjacent region and ablated regions of the embryo. Adjacent control region n = 25 cells, ablated region n = 14 cells from 5 embryos. Wilcoxon matched-pairs signed rank test, p < 0.0001. Y-scale cut to improve visualisation. All statistical tests 2-sided. Scale bar 50 μm (A), 40 μm (D). Source data

tNCs migrate under the somite through restricted spaces smaller than the resting size of their nucleus, and this correlates with the onset of nuclear shape changes. To directly test whether squeezing between the adjacent neural tube and somite tissues is causing neural crest nuclear deformations, we genetically perturbed tissue-scale confinement in the mid-trunk region by using the spadetail (*spt*) mutant, where somite formation is defective^39^. In *spt* mutants, tNC migration is unrestricted (Fig. 3a) due to reduced slow muscle tissue (Extended Data Fig. 3a)^40,41^. Injection of Neurocan-GFP mRNA and quantification of the width of inter-tissue spaces at the neural tube/somite interface indeed revealed the spaces along the tNC migratory path to be significantly wider in *spt* mutant embryos (Fig. 3b,c). We observed that the dorsolateral space between the EVL and the somite was also increased and some tNCs could invade this space^41^ (purple arrow). To monitor tNC migration, we generated sox10mG;*spt* mutant embryos and labelled the inter-tissue spaces using 10-kDa fluorescent dextran. We carried out in vivo live imaging to compare nuclear shape changes with those of control siblings (Fig. 3d). In *spt* mutant embryos, a larger fraction of tNCs tend to migrate along the dorsolateral path underneath the EVL^41^. These cells were excluded from our analysis as they colonized a different inter-tissue niche from control siblings. Instead, we specifically monitored nuclear shape changes for those NCs transitioning from the premigratory area to the inter-tissue spaces between the neural tube and somite (Fig. 3b, green arrow, Fig. 3d). We found that, in contrast to control sibling embryos, nuclear circularity and aspect ratios were unchanged in the *spt* mutants (Fig. 3e, Extended Data Fig. 3b and Supplementary Video 5). These findings suggest that the nucleus deformations in migrating tNCs are caused by tissue-scale confinement imposed by the surrounding somite tissues. However, because, in *spt* mutants, mesoderm formation is defective from very early stages of development^42–44^, we also developed an acute mechanical perturbation of somite formation to independently complement our *spt* findings. We used femtosecond pulsed IR-laser ablation in sox10:mG embryos to physically remove somite tissue. This method has been validated previously in zebrafish and used at similar developmental stages to target the notochord and the tailbud progenitors^45,46^. We precisely ablated a portion of the presomitic mesoderm forming midtrunk somites at 15 hpf (Fig. 3f and Extended Data Fig. 3c), preserving the premigratory neural crest, neural tube and notochord (Fig. 3g). This resulted in the formation of a gap in the somite segmental organization, while existing somites remained anterior to, and new somites formed posterior to, the ablated region (Fig. 3h and Extended Data Fig. 3c). Injection of 10-kDa dextran in ablated embryos showed a reduction in somite tissue on the ablated side of the embryo, and an increase in inter-tissue space, while somites on the contralateral side of the embryos were not affected (Extended Data Fig. 3d,e). To monitor the changes in nuclear shape, we carried out in vivo live imaging of sox10:mG in ablated embryos starting around 2 h after ablation when the premigratory to migratory transition took place at the ablated region. Similar to *spt*, somite ablation led to loss of migratory stream organization. Measurement of tNC nuclear shape changes upon transition to the inter-tissue spaces in adjacent, unaffected somites revealed a strong decrease in nucleus circularity (Fig. 3h–j and Supplementary Video 6) and a corresponding increase in aspect ratio (Extended Data Fig. 3f). In contrast, cells in the ablated region showed a more subtle, but still significant, decrease in nuclear circularity (Fig. 3h–j), while the nucleus aspect ratio remained unchanged (Extended Data Fig. 3f). Taken together, we demonstrate, using in vivo orthogonal genetic and mechanical perturbations, that tissue-scale confinement originating from the somites imposes nuclear deformations in tNCs during developmental cell migration.

### Confined migration of tNCs results in nucleo-cytoplasmic leakage

**Fig. 4:**
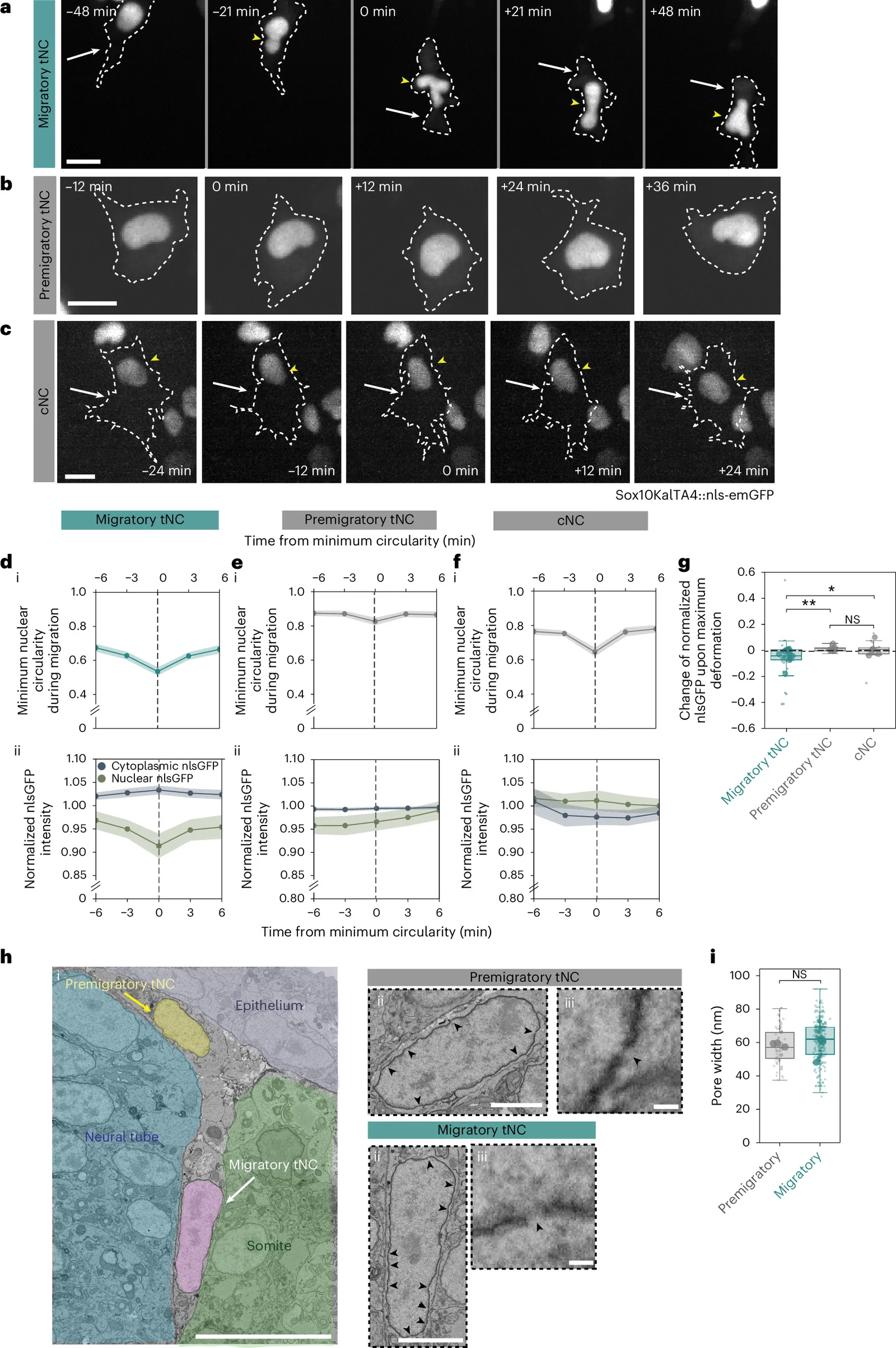
Confined migration of trunk neural crest results in nucleo-cytoplasmic leakage, but not in NE rupture. **a**–**c**, Representative time series of nls-emGFP localization during a nuclear deformation event in migratory (**a**) and premigratory NCs (**b**) in the mid trunk region and in migratory cNC cells (**c**). Yellow arrowheads indicate nuclear shape during migration. White arrows indicate the cytosolic nls-emGFP signal before, during and after the maximum deformation of the nucleus. Dashed lines indicate cell membranes. **d**–**f**, (i) Mean nuclear circularity during a nuclear deformation event and (ii) normalized nuclear (green) and cytoplasmic (blue) nls-emGFP intensity aligned to the minimum circularity event. Midtrunk, *n* = 50 migratory cells, nine embryos; *n* = 15 premigratory cells, six embryos; cNC, *n* = 15 deformation events from 13 cells, five embryos. Data are presented as mean and s.e.m. The *y* axis is cut to improve visualization. **g**, Difference in normalized nuclear nlsGFP between *t* = −6 min and *t* = 0 of maximum nuclear deformation in migratory tNC cells (*n* = 49 cells from nine embryos), premigratory tNC cells (*n* = 17 cells from six embryos) and cNC cells (*n* = 13 cells from five embryos). Kruskal–Wallis Dunn’s multiple comparisons test, **P = 0.0020, *P = 0.0271. Each dot represents a single deformation event, large dots represent mean values per embryo. **h**, (i) Representative image of an electron microscopy slice in the mid trunk region, (ii) representative zoomed images of single migratory and premigratory neural crest nuclei and (iii) further zoom-in of a single nuclear pore. The white arrow indicates a migratory cell; yellow arrow indicates a premigratory cell. Black arrowheads show the individual nuclear pores in the zoomed images. Pseudo colours facilitate the visualization of adjacent tissues and NC cells. **i**, Nuclear pore width in premigratory cells and migratory cells. Premigratory *n* = 3 embryos (63 nuclear pores); migratory *n* = 3 embryos (308 nuclear pores). Mann–Whitney test, *P* = 0.7. Cohen’s *d* effect size between individual pores, 0.2376. Each dot represents a single nuclear pore; large dots represent mean values per embryo. All outliers not shown. The box plots show the median, the first and third quartiles and the 1.5× inter-quartile range. All statistical tests two-sided. Scale bars, 10 μm (**a**–**c** and **h**), 3 μm (**h** (i)), 100 nm (**h** (ii)). Source data

**Extended Data Fig. 4:**
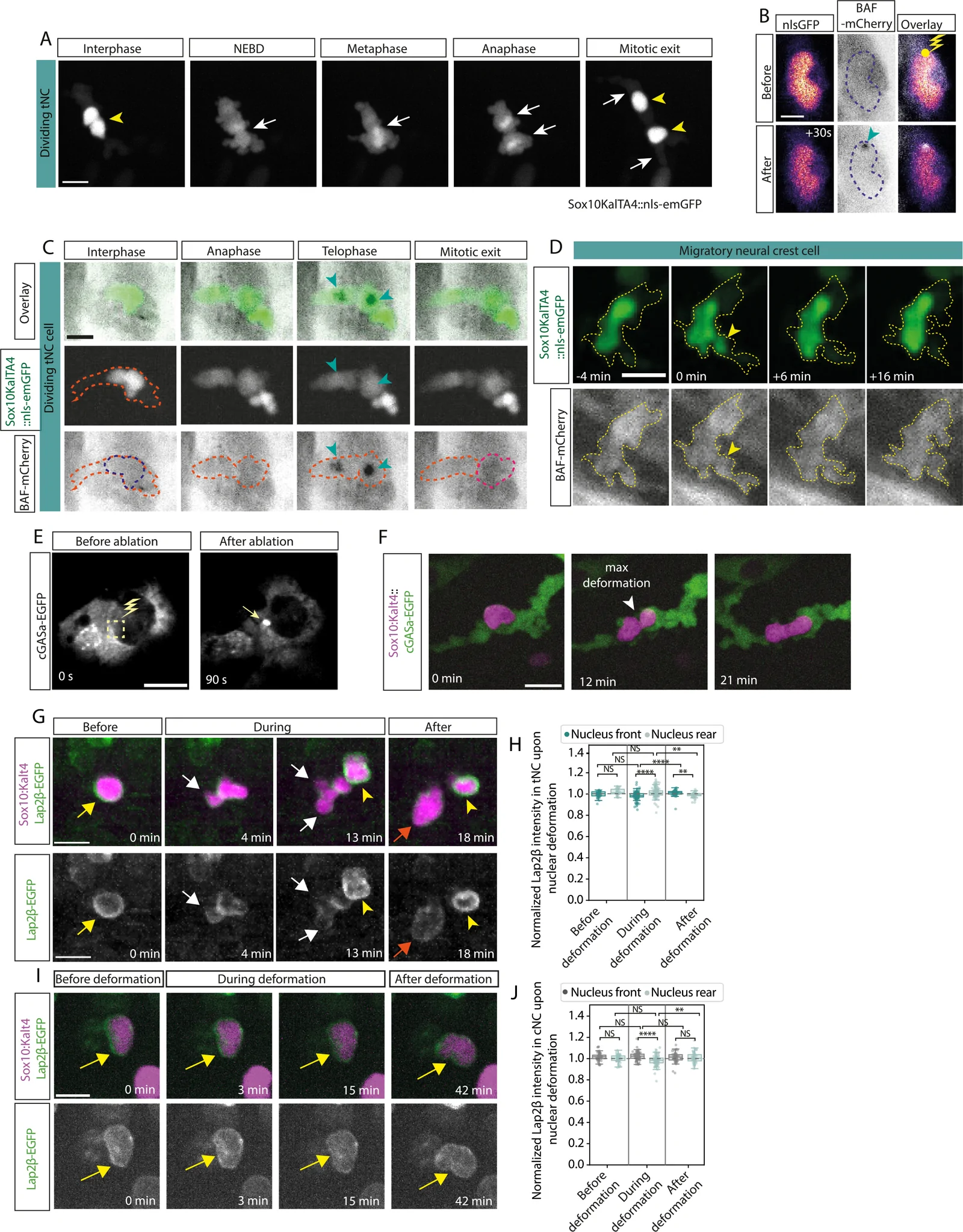
Neural crest do not experience nuclear envelope ruptures in vivo. **A)** Representative time series of nls-emGFP localization during cell division in migrating neural crest cell in mid trunk region. White arrowheads show the depletion of of nls-emGFP in the nucleus during cell division. Yellow arrowheads show the localization of nls-emGFP in nucleus in interface before cell division and the relocalization in the nuclei in daughter cells after division. Image representative of 10 embryos. **B)** Representative images of nls-emGFP and BAF-mCherry before and after laser ablation targeting nuclear envelope in nls-emGFP positive NC cell co-expressing BAF-mCherry mRNA. Green arrow indicated the re-localisation of BAF after the ablation. Lightning symbol and yellow circle indicate the region of ablation. n = 7 double-positive cell ablations from 2 embryos, n = 9 nls-emGFP cell ablations from 4 embryos. **C)** Representative time series of BAF localization during cell division in nls-emGFP positive migrating neural crest cell in mid trunk region. Cyan arrowheads show the rapid accumulation of BAF in telophase. Dashed outlines highlight the mother cell and daughter cells (orange, pink), and the nucleus of mother cell (blue). **D)** Representative time series of BAF localization during nuclear deformation event in nls-emGFP positive neural crest cell during migration in mid trunk region. Yellow arrowheads show the nuclear shape during maximum deformation event. Dashed outlines highlight the cell shape. C,D: images representative of 5 embryos. **E)** Representative images of a NC expressing cGASa-EGFP before and after laser ablation targeting the nuclear periphery in sox10:kalt4::cGASa-EGFP embryo (n = 20 cell ablations, 4 embryos). White arrow highlighting cGASa-EGFP localisation at the site of NE rupture after the ablation. Lightning symbol and yellow circle indicate the region of ablation. **F)** Representative time series of cGASa-EGFP localization during nuclear deformation event in sox10:kalt4::cGASa-EGFP neural crest cell during migration in mid trunk region. Arrowhead indicate nuclear shape during maximum deformation event. n = 7 embryos. **G)** Representative images of Lap2β localization in the nucleus of migratory cell in mid trunk region during nuclear deformation event. Yellow arrows show Lap2β localization at the leading front of the nucleus before deformation. White arrows show the depletion of lap2β at the bulking side of the deforming nucleus. Orange arrows show the relocalization of Lap2β at the leading front of the nucleus after deformation. Yellow arrowheads show Lap2β localisation in neighbouring cell nucleus without deformation. **H)** Normalised Lap2β intensity at the leading front and the rear of migratory trunk neural crest cell nucleus before, during and after nuclear deformation event. Before deformation n = 39 timepoints from 15 cells, during deformation n = 132 timepoints from 15 cells, after deformation n = 48 timepoints from 15 cells from 4 embryos. Each dot represents a timepoint. Kruskal-Wallis Dunn’s multiple comparison test: p = 0.0073 (after deformation), p = 0.0020 (rear during vs after deformation), p < 0.0001 (****). **I)** Representative images of Lap2β localization in the nucleus of a migratory cell in cranial region during nuclear deformation event. Yellow arrows show Lap2β localization at the leading front of the nucleus. **J)** Normalised Lap2β intensity at the leading front and the rear of migratory cranial neural crest cell nucleus before, during and after nuclear deformation event. Before deformation n = 62 timepoints from 19 cells, during deformation n = 92 timepoints from 19 cells, after deformation n = 50 timepoints from 19 cells. n = 5 embryos. Each dot represents a timepoint. Kruskal-Wallis Dunn’s multiple comparison test: p = 0.0089 (**), p < 0.0001 (****). Box plots show the median, the 1st and 3rd quartile and the 1.5x inter-quartile range. All statistical tests 2-sided. Scale bar 10 μm (A,C,D,G,I), 5 μm (B). Source data

Our results show that during developmental migration in narrow inter-tissue spaces, tNCs undergo striking, repeated nuclear deformations. Cells are prone to nucleus envelope (NE) ruptures in vitro below a critical confinement threshold of 3 μm (refs. ^2,9–14^). To ask whether nucleus deformations observed in migrating tNCs lead to NE ruptures, we focused on tNCs at the mid trunk region, as we observed the highest discrepancy between tissue space and nucleus size in this region (Fig. 2e,f and Extended Data Fig. 2d). To interrogate NE ruptures, we live-imaged tNC cells expressing a nuclear-localized emerald-GFP (nls-emGFP) under control of a neural crest-specific Gal4 (ref. ^47^). To validate that nls-emGFP could be used as a proxy for NE integrity in zebrafish, we imaged tNC cell divisions (Extended Data Fig. 4a). Nls-emGFP fluorescence strongly increased in the cytoplasm at mitotic entry (Extended Data Fig. 4a, white arrows), followed by sequestration of nls-emGFP back within the nucleus at mitotic exit (Extended Data Fig. 4a, yellow arrowheads). IR-laser ablation of the nucleus periphery in interphase cells also resulted in loss of nuclear nls-emGFP and a concomitant increase in cytoplasmic nls-emGFP (Extended Data Fig. 4b). We then carried out in vivo live imaging of nls-emGFP in migrating tNCs (Fig. 4a and Supplementary Video 7) and premigratory tNCs to assess NE integrity (Fig. 4b and Supplementary Video 8). Quantification of nls-emGFP nuclear intensity throughout deformation revealed leakage of a small fraction of nls-emGFP into the cytoplasm and a simultaneous decrease in nuclear nls-emGFP intensity upon maximum nuclear deformation (Fig. 4a,d(i,ii),g). nls-emGFP relocalization was not observed in premigratory tNCs, where nuclear deformation events were subtle (Fig. 4b,e(i,ii),g).

We also monitored nls-emGFP nucleo-cytoplasmic leakage in non-confined cNCs (Fig. 2a,c,d). We found that, like premigratory tNC, cNCs did not show notable changes in nls-emGFP nuclear or cytoplasmic intensity upon nucleus shape changes (Fig. 4c,f,g). These findings suggest that confined migratory tNC cells, but not non-confined premigratory tNCs or cNCs, show a transient nucleo-cytoplasmic leakage. Quantification of nls-emGFP only showed a mild 5% decrease in nuclear intensity, accompanied by a correspondingly small increase in cytoplasmic intensity in migrating tNCs. Following NE ruptures, chromatin becomes exposed to the cytoplasm and can be detected by cytoplasmic DNA-binding proteins, such as BAF^11,48^ or cGAS^12,13^. Therefore, we further investigated NE ruptures using BAF-mCherry^11,48^. We validated the BAF-mCherry reporter by observing a transient accumulation of BAF in late anaphase (Extended Data Fig. 4c), as previously reported in human cells^49^ and with IR-laser ablation, resulting in accumulation of BAF-mCherry at the ablation site (Extended Data Fig. 4b, green arrowhead). However, we were not able to identify BAF positive foci at the nuclear periphery of migrating mid tNC cells, even during extreme nuclear deformation events (Extended Data Fig. 4d and Supplementary Video 9). Furthermore, live imaging of a neural-crest-specific cGAS-EGFP reporter also did not reveal NE ruptures, whereas cGAS readily localized to sites of IR-laser-induced NE ruptures (Extended Data Fig. 4e,f and Supplementary Video 10). Altogether, these results argue against a loss of NE integrity, suggesting that the nucleo-cytoplasmic leakage of nls-emGFP might be a result of nuclear pore stretching^50,51^. To test this possibility, we carried out scanning electron microscopy (SEM) of wild-type zebrafish trunk cross-sections to visualize the NE of confined tNCs in detail. SEM revealed an intact NE in migrating tNCs (Fig. 4h(i,ii,iii), arrows and arrowheads). Measurements of nuclear pore width in migratory cells suggested a large distribution of nuclear pore sizes compared to premigratory tNCs, possibly suggesting dynamic changes in nuclear pore size under in vivo confinement (Fig. 4i). Together, our results show that nuclear deformation events cause a passive leakage of nls-emGFP in confined tNCs in vivo. As our results did not indicate a loss of NE integrity, to further resolve dynamic changes of the NE, we mosaically injected mRNA encoding the LEM-domain protein Lap2β-EGFP^8^ into sox10Kalt4 embryos, where chromatin is labelled with H2B-mCherry^52^ (Extended Data Fig. 4g and Supplementary Video 11). High spatiotemporal resolution of migrating tNC cells revealed that Lap2β-EGFP appears dynamically depleted from the front of the nucleus, resulting in a polarized distribution of Lap2β-EGFP at the NE during nuclear deformation events, which recovers after deformation (Extended Data Fig. 4h and Supplementary Video 11). In contrast, in non-confined migrating cNCs, Lap2β-EGFP shows an opposite polarized localization (Extended Data Fig. 4i,j). Lap2β links chromatin to the nuclear periphery^53,54^ and mediates force transmission from the nuclear lamina to chromatin upon stretching^55^. Hence, localized reduction of Lap2β at the nucleus front may reduce tethering of chromatin, facilitating the passage of the nucleus through small tissue spaces in vivo.

### In vivo confined migration of tNCs does not lead to accumulation of DNA double-strand breaks

**Fig. 5:**
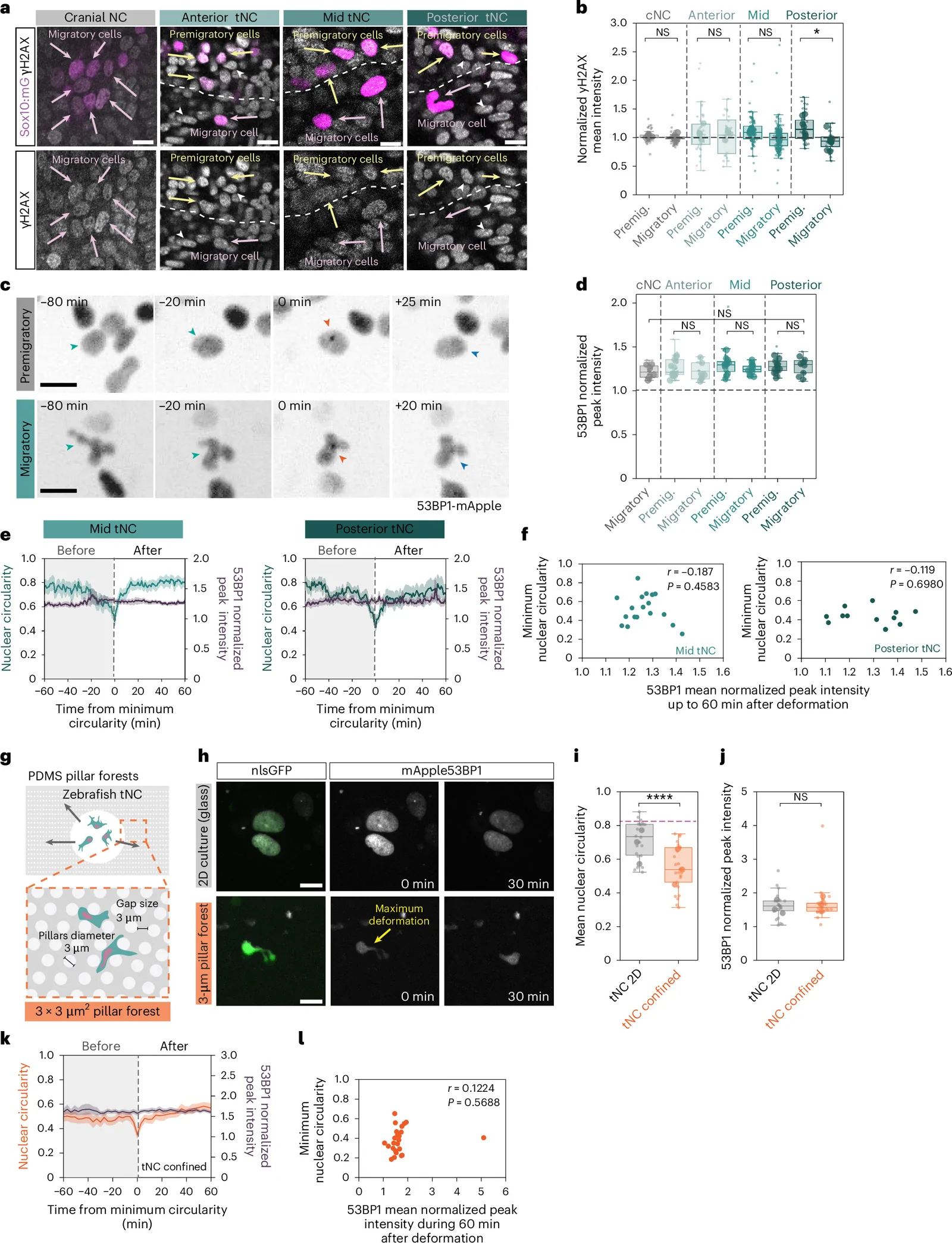
NCs do not incur DNA damage during in vivo or in vitro confined migration. **a**, Representative single *z*-slices showing γH2AX immunostaining in premigratory (yellow arrows) and migratory NCs (pink arrows) in different AP regions. Dashed lines indicate premigratory region/somite boundaries; white arrowheads highlight the intensity levels in surrounding tissues. Overlay, sox10:mG (neural crest, magenta; γH2AX, grey). **b**, γH2AX intensity in premigratory and migratory NC cells normalized to surrounding tissues in cranial, anterior trunk, mid trunk and posterior trunk. cNC, *n* = 5 embryos (premigratory region 63 cells, migratory region 136 cells); anterior, *n* = 4 embryos (premigratory region 42 cells, migratory region 24 cells); mid trunk, *n* = 6 embryos (premigratory region 87 cells, migratory region 117 cells); posterior, *n* = 7 embryos (premigratory region 45 cells, migratory region 36 cells). Kruskal–Wallis Dunn’s multiple comparison test between embryos, *P = 0.0195. Small dots represent a single nucleus; large dots represent mean values per embryo. **c**, Representative time series of the maximum projection of mApple-53BP1 localization in migratory and premigratory NCs in the mid trunk region. Cyan arrowheads show the nuclear shape before 53BP1 foci appearance, red arrowheads indicate 53BP1 foci in the nucleus, and blue arrowheads indicates the nuclear shape after foci disappear. **d**, Mean 53BP1 normalized peak intensity per nucleus in premigratory and migratory NC cells in different AP regions. cNC, *n* = 20 cells, six embryos; anterior, *n* = 35 premigratory cells, 11 embryos, *n* = 15 migratory cells, six embryos; mid trunk, *n* = 27 premigratory cells, nine embryos; *n* = 18 migratory cells, 10 embryos; posterior, *n* = 31 premigratory cells, nine embryos; *n* = 14 migratory cells, eight embryos. Kruskal–Wallis test, NS, *P* = 0.0897. Small dots represent a mean normalized 53BP1 value of a single nucleus over time; large dots represent mean values per embryo. **e**, Nuclear circularity and normalized 53BP1 peak intensity aligned to minimum circularity upon NC migration in the mid trunk and posterior regions. Mid trunk *n* = 15 cells, 10 embryos; posterior trunk *n* = 14 cells, six embryos. Data are presented as mean and s.e.m. Dashed lines separate time before (grey) and after maximum deformation. **f**, Scatter plots showing the correlation of minimum nuclear circularity value and mean value of normalized 53BP1 peak intensity up to 60 min after the maximum deformation event in NC cells at the mid trunk and posterior regions. Mid trunk *n* = 18 cells, 10 embryos; posterior trunk *n* = 13 cells, six embryos. Pearson correlation test; *r* and *P* values are indicated in each scatter plot. **g**, Sketch of NCs in confined in vitro culture condition in a PDMS pillar forest. **h**, Representative time series of cultured trunk NC cells expressing nlsGFP and mApple-53BP1 in 2D and under confinement during and 30 min after the maximal nuclear deformation event. Yellow arrow indicates the maximum nuclear deformation. **i**, Mean nuclear circularity per nucleus in trunk NC cells in 2D and in confinement. 2D *n* = 22 cells, confinement *n* = 25 cells, three independent experiments. Mann–Whitney test, ****P < 0.0001. Small dots represent the mean circularity value of a single nucleus over time; large dots represent mean values per experiment. The dashed horizontal purple line indicates the mean nuclear circularity for in vivo migrating tNC cells. **j**, Mean 53BP1 normalized peak intensity per nucleus in NC cells in 2D and in confinement. 2D *n* = 22 cells, confinement *n* = 25 cells, three independent experiments. Mann–Whitney test, NS, *P* = 0.8039. Each dot represents the mean normalized 53BP1 value of a single nucleus over time. **k**, Nuclear circularity and normalized 53BP1 peak intensity aligned to the minimum circularity upon NC migration in confined culture conditions. *n* = 20 cells, two independent experiments. Data are presented as mean and s.e.m. The dashed line separates the time before (grey) and after the maximum deformation. **l**, Scatter plot showing the correlation of minimum nuclear circularity value and mean value of normalized 53BP1 peak intensity up to 60 min after the maximum deformation event in NC cells in confined culture conditions. *n* = 24 cells, three independent experiments. Pearson correlation test; *r* and *P* values are indicated in the plot. The box plots show the median, the first and third quartiles and the 1.5× interquartile range. All statistical tests are two-sided. Scale bars, 10 μm (**a**), 20 μm (**c**,**h**). Source data

**Extended Data Fig. 5:**
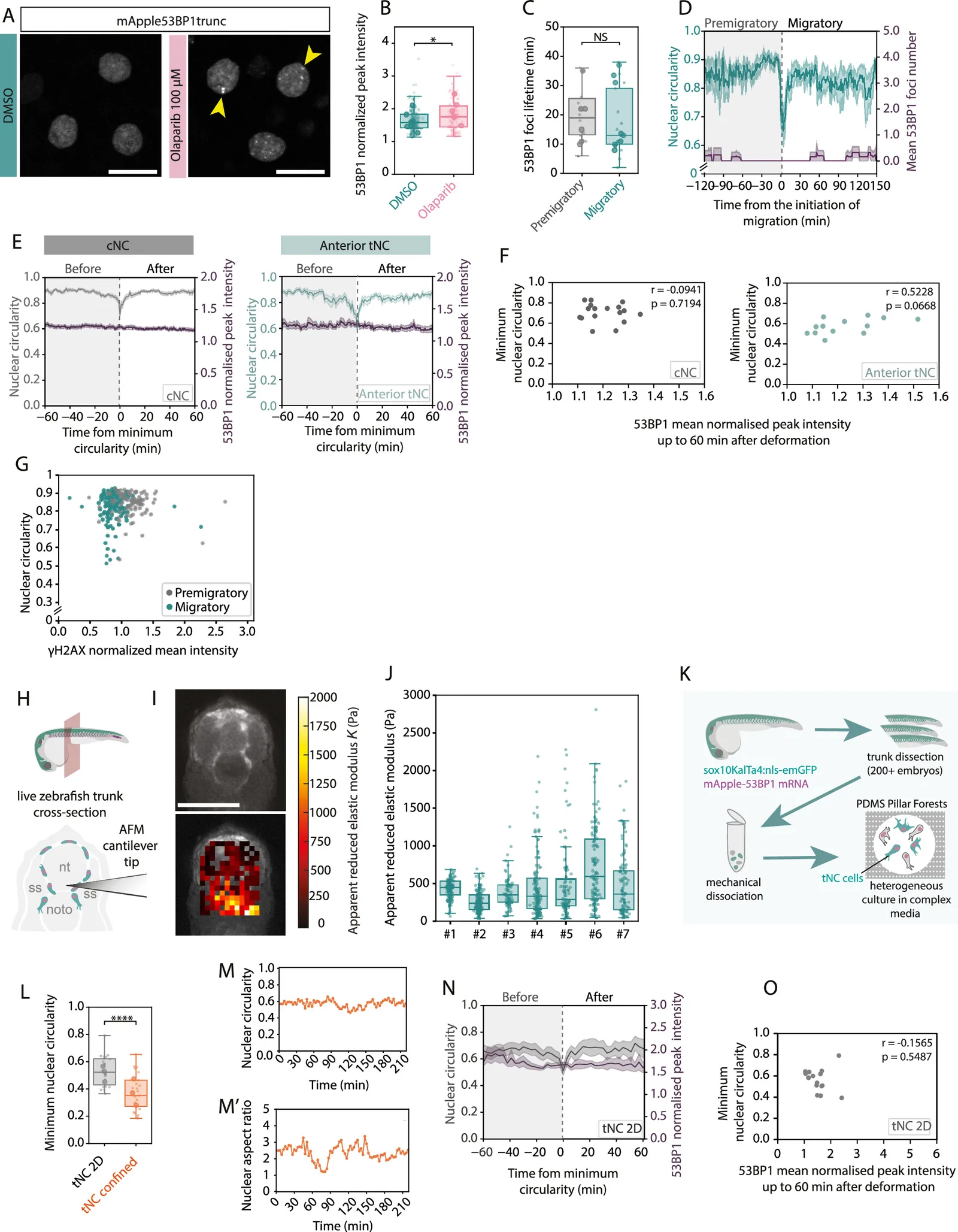
Neural crest do not accumulate DNA damage under stiff in vitro confinement. **A)** Representative images of 53BP1 accumulation in nucleus after olaparib treatment. Yellow arrowheads highlight the localization of 53BP1 foci**. B)** 53BP1 normalised peak intensity in olaparib treated and DMSO control cells at shield stage. DMSO n = 188 cells from 8 embryos, olaparib n = 115 cells from 6 embryos. Unpaired t-test; p = 0.0329. Each dot represents a nucleus. **C)** 53BP1 foci lifetime in neural crest cells in mid trunk region during premigratory and migratory phase. Foci in premigratory phase n = 14 from 11 cells from 7 embryos, migratory phase n = 17 foci from 14 cells from 8 embryos. Mann-Whitney test, p = 0.4793. Small dot represents a foci, large dots represent a mean per embryo. All outliers not shown. **D)** Nuclear circularity over time aligned to the transition from premigratory region to migratory region together with mean number of 53BP1 foci in foci positive cells in midtrunk reagion. n = 8 cells from 6 embryos. Mean and SEM presented. Y-scale cut to improve visualisation. **E)** Nuclear circularity and normalised 53BP1 peak intensity aligned to the minimum circularity upon NC migration at different cNC and anterior trunk regions, CNC n = 17 cells from 6 embryos; anterior trunk n = 14 cells from 6 embryos. Mean and SEM presented. Dashed line separates the time before (grey) and after maximum deformation. **F)** Scatter plots showing correlation of minimum nuclear circularity value and mean value of normalised 53BP1 peak intensity up to 60 minutes after maximum deformation event in NC cells at cNC and anterior trunk regions, NC n = 17 cells from 6 embryos; anterior trunk n = 13 cells from 6 embryos. Pearson Correlation test, r and p value indicated in each scatter plot. **G)** Normalised yH2AX intensity over nuclear circularity values in premigratory and migratory trunk neural crest cells. Premigratory n = 89 cells from 7 embryos. Spearman r = 0.08, p = 0.39, migratory n = 131 cells from 7 embryos. Spearman r = -0.03, p = 0.77. **H)** Sketch representing live sectioning method for AFM: a 500 μm-thick slice was sectioned from a 24hpf sox10:mG embryo embedded in 2% agarose and prepared for AFM (also see Methods). **I)** Example of tissue cross-section and (I) same image overlaid with the recorded elasticity heat map (animal #4). Every pixel represents one measurement; colours represent apparent reduced elastic modulus *K* (darker colours – softer; brighter colours – stiffer). **J)** To assess tissue elasticity, AFM measurements were conducted on fresh transverse tissue sections from Sox10mG zebrafish larvae at 24 hpf in the mid trunk region and quantified per animal (n = 67 -136 AFM measurements/animal, from 7 embryos). **K)** Sketch of heterogeneous tNC culture protocol: trunks of ~200 sox10KaltA4::nls-emGFP embryos injected with 53BP1 mRNA are dissected with fine scissors, mechanically dissociated in PBS and then plated on fibronectin coated pillar forests in complex medium (see Methods for details). **L)** Minimum nuclear circularity per nucleus in trunk NC cells in 2D and in confinement. 2D n = 22 cells, confinement n = 25 cells. Mann-Whitney test, p < 0.0001. Small dot represents a single nucleus, large dots represent a mean value per experiment. **M)** Example of nuclear shape fluctuation in a single trunk NC crest in confined culture condition presented in nuclear circularity (M) and nuclear aspect ratio (M’). **N)** Nuclear circularity and normalised 53BP1 peak intensity aligned to the minimum circularity upon NC migration in 2D culture conditions. n = 12 cells. Mean and SEM presented. Dashed line separates the time before (grey) and after maximum deformation. **O)** Scatter plot showing correlation of minimum nuclear circularity value and mean value of normalised 53BP1 peak intensity up to 60 minutes after maximum deformation event in NC in 2D culture conditions. n = 17 cells. Pearson Correlation test, r and p value indicated in each scatter plot. Box plots show the median, the 1st and 3rd quartile and the 1.5x inter-quartile range All statistical tests 2-sided. Scale bars 20 µm (A),100 µm (I). Source data

Our results show that during developmental migration, NCs dramatically deform the nucleus, which results in passive leakage of nls-emGFP across the nucleo-cytoplasmic barrier. In cultured cells, NE ruptures or nuclear deformation itself^16^ can result in an accumulation of DNA damage^12–14,56^. We thus asked whether the striking nucleus deformations we observed in tNCs during migration (Fig. 1a,c,d and Fig. 4a,d(i,ii)) could result in DNA damage. We immunostained sox10:mG embryos for phosphorylated γH2AX to visualize DNA double-strand breaks^57^ across NC subpopulations. Surprisingly, we observed that migratory NCs do not show a significant increase in γH2AX fluorescence intensity compared to premigratory NCs (Fig. 5a, compare pink with yellow arrows, and Fig. 5b), and for the most strongly deformed posttNC population, γH2AX fluorescence intensity was significantly decreased (Fig. 5a,b). To investigate whether dynamic DNA damage hotspots could be detected in live embryos, we measured the dynamics of DNA damage foci formation and resolution in live embryos. We generated mRNA encoding the minimal foci-forming region of mApple-53BP1, which localizes to DNA double-strand breaks^58^. To validate this reporter in the zebrafish, blastula-stage embryos were treated with the PARP inhibitor olaparib^57,58^ to induce DNA double-strand breaks, which were readily detected as 53BP1 subnuclear foci (Extended Data Fig. 5a,b). To ask whether migrating NC subpopulations experience DNA damage as a consequence of in vivo confined cell migration, we mosaically injected mApple-53BP1mRNA in sox10KalTA4::nls-emGFP embryos. This allowed us to concomitantly monitor nuclear shape changes and 53BP1 fluorescence intensity. For the fraction of cells that did form one or more trackable foci during live imaging, foci lifetime was in the timescale of minutes for both premigratory and migratory tNCs (Supplementary Videos 12 and 13, Fig. 5c and Extended Data Fig. 5c) and the number of foci remained low during deformation (Extended Data Fig. 5d). As most mid tNCs (53.8%) did not show trackable 53BP1 foci (Supplementary Videos 12 and 13), we measured the ratio between the maximum and mean fluorescence intensities (normalized peak intensity) to detect transient, more dynamic, fluctuations in the 53BP1 signal. Comparison of the normalized 53BP1 peak intensity in premigratory and migratory regions did not show notable changes in NC cells across the AP regions (Fig. 5d). In mammalian cells, 53BP1 intensity can increase with a delay relative to the nucleus deformation event^10–14^. To directly interrogate whether DNA damage could occur as a consequence of nucleus deformations in vivo, we measured 53BP1 normalized peak intensity for up to 1 h before and after the maximum nucleus deformation (Fig. 5e). We observed that the 53BP1 normalized peak intensity remained relatively constant even for the highly deformed mid tNC and posttNC (Fig. 5e). To ask whether cells undergoing stronger deformations would incur DNA damage, we carried out correlation analysis between the minimum circularity of each cell and the normalized peak intensity of 53BP1 up to 60 min following nucleus deformation (Fig. 5f). However, we did not detect any significant correlation between nucleus deformation and DNA damage onset across NC populations. Similarly, DNA damage remains constant in cNC and atNC populations over time (Extended Data Fig. 5e,f). Consistent with our live observations, we confirmed no correlation between tNC nucleus circularity and endogenous γH2AX intensity in mid tNCs (Extended Data Fig. 5g). Overall, our results show that in migrating, confined tNCs, endogenous DNA damage levels are similar or lower than in non-confined counterparts. We observed that migration of tNCs in highly confined tissue surroundings does not trigger DNA damage accumulation and, furthermore, we did not detect a correlation between the level of nuclear deformation and DNA damage response. We conclude that tNCs do not accumulate DNA damage during in vivo confined cell migration.

### Stiff in vitro confinement does not induce de novo DNA damage in tNCs

We hypothesized that tNCs migrating through physiologically soft embryonic tissues may not experience sufficient mechanical stresses from their surroundings to trigger DNA damage. To directly test this hypothesis, we measured the stiffness of zebrafish trunk tissues by carrying out atomic force microscopy of thick, live zebrafish mid-trunk cross-sections (Extended Data Fig. 5h–j). Consistent with previous reports^59^, zebrafish trunk tissues appear soft (~0.4 kPa) compared to the polydimethylsiloxane (PDMS) material often used in microfabrication studies (PDMS stiffness of ~1.3 MPa)^60^. To test whether NCs accumulate DNA damage when confined within rigid, non-deformable boundary conditions, we cultured primary zebrafish tNCs^61^. To mimic in vivo repeated cycles of nucleus deformation and recovery (Extended Data Fig. 1e), we confined primary zebrafish tNCs in PDMS pillar forests^62^ with pillars spaced at a distance of 3 μm from one another (Extended Data Fig. 5k and Fig. 5g). tNC cells under in vitro confinement showed significantly increased nuclear deformation in comparison to tNCs in vitro in 2D (Fig. 5h,i) and to in vivo migration (Fig. 5i, Extended Data Fig. 5l,m,m’; also Fig. 1c and Extended Data Fig. 1f’), further supporting that in vitro tNCs might experience a higher level of mechanical stress than in vivo. We assessed the DNA damage response to confinement using the mApple-53BP1 live mRNA reporter (Fig. 5h,j–l). Despite sustained nucleus deformations, tNC cells under in vitro confinement did not show an increased 53BP1 peak intensity compared to cells in 2D (Fig. 5j). Furthermore, the 53BP1 peak intensity remained constant after a maximum deformation event both in 2D and under confined culture conditions. No correlation was found between the maximum nuclear deformation and the 53BP1 response up to 60 min later (Fig. 5k,l and Extended Data Fig. 5n,o). Altogether, these results reveal that that tNCs do not incur an increase in DNA damage, even under stiff in vitro confinement.

### LaminB2 regulates neural crest nuclear shape changes

**Fig. 6:**
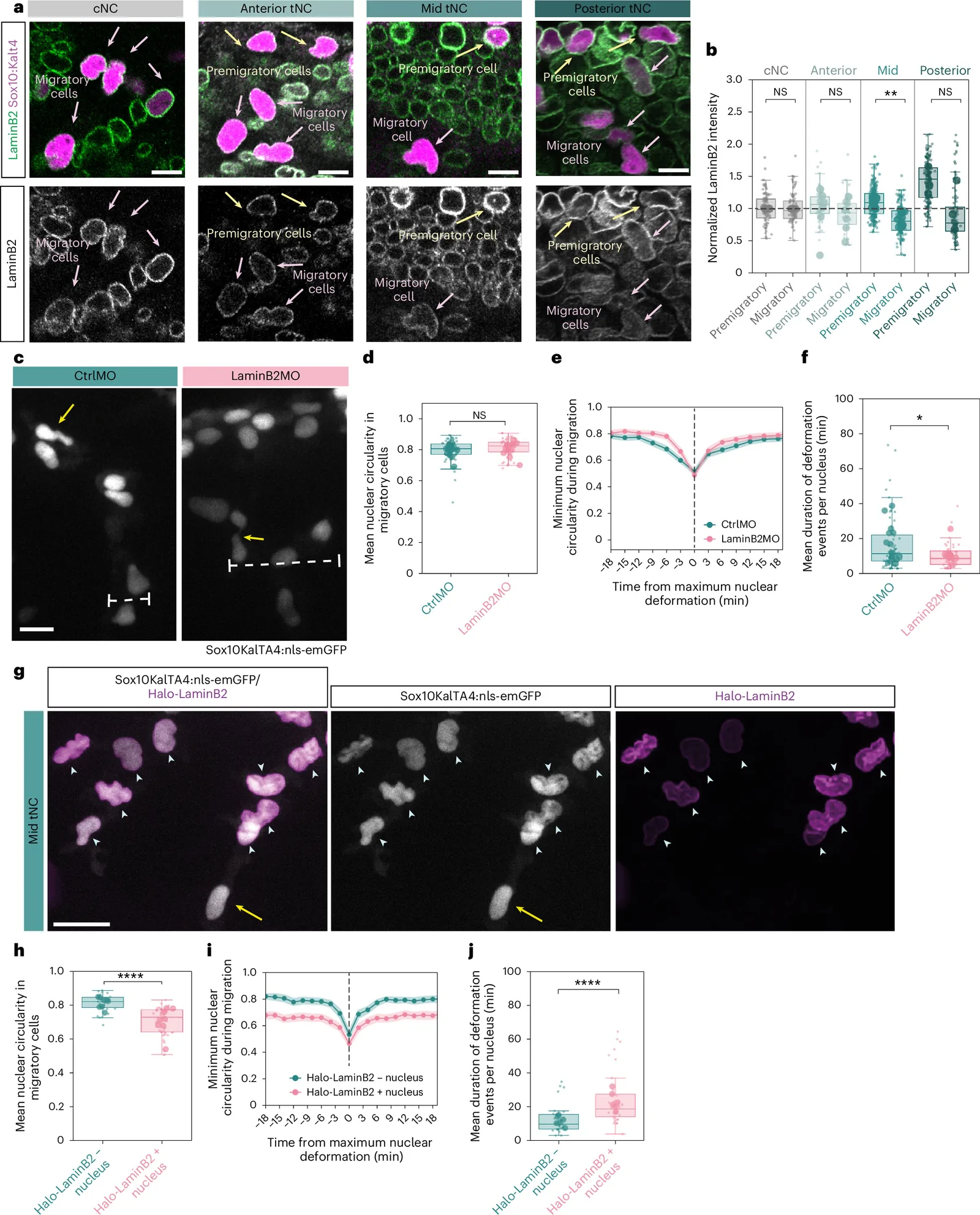
LaminB2 regulates neural crest nuclear shape changes in confinement. **a**, Representative images of the expression of nuclear LaminB2 in premigratory (yellow arrows) and migratory (pink arrows) NCs across cNC, atNC, mid tNC and posttNC regions (single *z* slices, overlay: sox10Kalt4, LaminB2). **b**, Nuclear LaminB2 intensity in premigratory and migratory NCs normalized to the mean LaminB2 intensity in cranial, anterior trunk, mid trunk and posterior trunk. cNC, *n* = 6 embryos (80 premigratory cells, 80 migratory cells); anterior, *n* = 6 embryos (56 premigratory cells, 40 migratory cells); mid trunk, *n* = 9 embryos (244 premigratory cells, 135 migratory cells); posterior, *n* = 3 embryos (150 premigratory cells, 93 migratory cells). Kruskal–Wallis Dunn’s multiple comparison test between embryos, **P = 0.0041. Small dots represent a single nucleus; large dots represent mean value per embryo. **c**, Representative maximum projection of migratory NCs at the midtrunk region in control morpholino (CtrlMO) and LaminB2 morpholino (LaminB2MO) injected nlsGFP embryos. Yellow arrows indicate nuclear deformation events. White dashed lines indicate the width of the NC streams during migration. **d**, Mean nuclear circularity per nucleus in migratory trunk NC cells in CtrlMO and laminB2MO-injected embryos. CtrlMO *n* = 67 cells, 14 embryos; laminB2MO *n* = 54 cells, 12 embryos. Mann–Whitney test, NS, *P* = 0.1150. Small dots represent the mean circularity value of a single nucleus over time; large dots represent the mean value per embryo. **e**, Mean nuclear circularity aligned to the minimum circularity event during midtrunk NC migration in CtrlMO and LaminB2MO-injected embryos. CtrlMO *n* = 67 cells, 14 embryos; LaminB2MO *n* = 54 cells, 12 embryos. Data are presented as mean ± s.e.m. **f**, Mean duration of mid tNC nuclear deformation events per nucleus in CtrlMO and LaminB2MO injected embryos, calculated as consecutive timepoints the nucleus circularity remains under the mean circularity value. CtrlMO *n* = 67 cells, 13 embryos; LaminB2MO *n* = 48 cells, nine embryos. Mann–Whitney test, *P = 0.0376, Cohen’s *d* effect size, 0.4614743. Each dot represents a mean duration of deformations per nucleus; large dots represent the mean values per embryo. **g**, Representative time series of the maximum projection of Halo-laminB2-positive (magenta) and nlsGFP (grey) double-positive migratory NC cells in the midtrunk region. White arrowheads indicate LaminB2-positive cells; yellow arrows indicate negative cells within the same embryos. **h**, Mean nuclear circularity per nucleus during trunk neural crest migration in Halo-LaminB2-negative (−) and Halo-LaminB2-positive (+) cells. Negative cells *n* = 30, eight embryos; positive cells *n* = 37, seven embryos. Mann–Whitney test, ****P < 0.0001. Small dots represent mean circularity values of a single nucleus over time; large dots represent mean values per embryo. **i**, Mean nuclear circularity aligned to the minimum circularity event during trunk neural crest migration in Halo-LaminB2-negative and Halo-LaminB2-positive cells. Negative cells *n* = 28, eight embryos; positive cells *n* = 34, seven embryos. Data are presented as mean and s.e.m. **j**, Mean duration of mid tNC nuclear deformation events per nucleus in Halo-laminB2 negative and Halo-laminB2 positive cells, calculated as consecutive timepoints the nucleus circularity remains under the mean circularity value. Negative cells *n* = 32, six embryos; positive cells *n* = 32, seven embryos. Mann–Whitney test: ****P < 0.0001. Each dot represents a mean duration of deformations per nucleus; large dots represent mean values per embryo. The box plots show the median, the first and third quartiles and the 1.5× interquartile range. All statistical tests are two-sided. Scale bars, 10 μm (**a**,**c**), 20 μm (**g**). Source data

**Extended Data Fig. 6:**
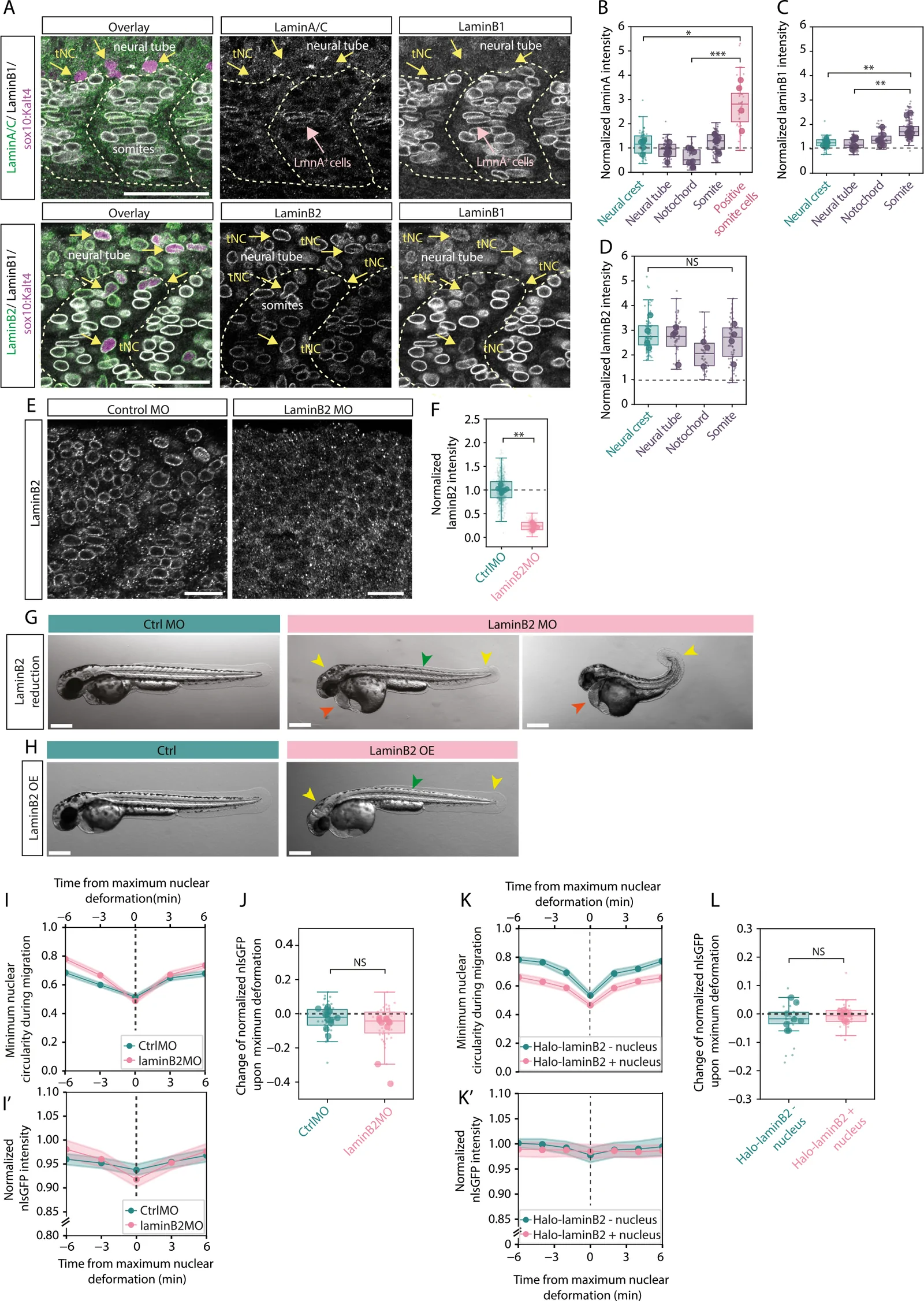
Quantifications of endogenous nuclear lamins. Perturbation of LaminB2 levels does not impact nucleo-cytoplasmic leakage. **A)** Representative single z-slices of LaminA/C and LaminB1 immunostaining in mid trunk region (top). Dashed lines indicate somites. Yellow arrows indicate neural crest cells, pink arrows indicate LaminA^+^ somite cells (LmnA^+^). Overlay: sox10Kalt4 (neural crest), LaminA, LaminB1. Representative single z-slices of LaminB2 and LaminB1 immunostaining in mid trunk region (bottom). Dashed lines indicate somites. Yellow arrows indicate neural crest cells. Overlay: sox10Kalt4 (neural crest), LaminB2, LaminB1. **B)** Quantification of LaminA immunostaining NE (nuclear envelope)/cytoplasm ratio across mid-trunk zebrafish tissues. Neural crest n = 6 embryos (70 nuclei), neural tube n = 7 embryos (141 nuclei), notochord n = 7 embryos (92 cells), somites n = 7 embryos (138 cells), positive somite cells n = 4 embryos (43 cells). Kruskal-Wallis Dunn’s multiple comparison test: p = 0.0139 (*), p = 0.0009 (***). **C)** Quantification of LaminB1 immunostaining NE (nuclear envelope)/cytoplasm ratio across mid-trunk zebrafish tissues. Neural crest n = 7 embryos (133 nuclei), neural tube n = 8 embryos (178 nuclei), notochord n = 8 embryos (118 cells), somites n = 8 embryos (199 cells). Kruskal-Wallis Dunn’s multiple comparison test: p = 0.0027 (neural crest vs somite), p = 0.0023 (neural tube vs somite). Small dots represent a single nucleus, large dots represent a mean value per embryo. **D)** Quantification of LaminB2 immunostaining NE (nuclear envelope)/cytoplasm ratio across mid-trunk zebrafish tissues. Neural crest n = 4 embryos (133 nuclei), neural tube n = 4 embryos (178 nuclei), notochord n = 3 embryos (118 cells), somites n = 4 embryos (199 cells). Kruskal-Wallis Dunn’s multiple comparison test: p = 0.5023. Small dots represent a single nucleus, large dots represent a mean value per embryo. **E)** Representative image of LaminB2 antibody staining in CtrlMO and laminB2MO injected embryos. **F)** Nuclear laminB2 intensity in CtrlMO and laminB2MO injected embryos normalised to the mean intensity of nucleus in CtrlMO injected embryos. CtrlMO n = 6 embryos (981 cells), laminB2MO n = 5 embryos (504 cells). Mann-Whitney test: p = 0.0043. Small dots represent a single nucleus, large dots represent a mean value per embryo. **G)** Representative images of CtrlMO and variety of phenotypes in laminB2MO injected embryos at 2dpf (days post fertilization) in WT background. Yellow arrowheads highlights small head and short tails in laminB2 deficient embryos. Green arrow indicates the mislocalisation of pigment cells. Red arrows indicates heart oedema. **H)** Representative images of negative and positive laminB2 OE embryos at 2dpf from an outcross of UAS-laminB2-Halo and Sox10:inducible:Kalt4 fish. LaminB2 OE fish were identified by green heart at 24 hpf. Yellow arrowheads highlights small eye and short tail in laminB2 OE embryo. Green arrow indicates the mislocalisation of pigment cells. **I)** Mean nuclear circularity during minimum circularity event during trunk neural crest migration in CtrlMO and laminB2MO injected embryos (I’) and normalised nls-emGFP intensity in nucleus aligned to the minimum circularity event. CtrlMO n = 67 cells from 14 embryos, laminB2MO n = 54 cells from 12 embryos. Mean and SEM presented. Y-scale cut in (I’) to improve visualisation. **J)** Difference in normalised nuclear nlsGFP between 6 minutes before and during maximum deformation event in migratory mid trunk NC cells in CtrlMO and laminB2MO injected embryos. CtrlMO n = 56 cells from 14 embryos, laminB2MO n = 46 cells from 12 embryos. Mann-Whitney test, p = 0.0719. Small dots represent a single nucleus, large dots represent a mean value per embryo. **K)** Mean nuclear circularity during minimum circularity event during trunk neural crest migration in Halo-laminB2 negative and Halo-laminB2 positive cells (K’) and normalised nls-emGFP intensity in nucleus aligned to the minimum circularity event. Negative cells n = 28 from 8 embryos, positive cells n = 34 from 8 embryos. Mean and SEM presented. Y-scale cut in (K’) to improve visualisation. **L)** Difference in normalised nuclear nlsGFP between 6 minutes before and during maximum deformation event in migratory mid trunk NC cells in Halo-laminB2 negative and Halo-laminB2 positive cells. Positive cells n = 28 cells from 8 embryos, negative cells n = 34 cells from 8 embryos. Mann-Whitney test, p = 0.3075. Small dots represent a single nucleus, large dots represent a mean value per embryo. Box plots show the median, the 1st and 3rd quartile and the 1.5x inter-quartile range. All statistical tests 2-sided. Scale bars 50 μm (A), 20 μm (E), 500 μm (G,H). Source data

Our results show that nuclear deformation does not trigger DNA damage in migrating tNCs. We next asked whether the neural crest nucleus may dynamically adapt to confinement to reduce stress. To test this hypothesis, we investigated the lamina composition of neural crest nuclei by immunostaining for LaminA/C, LaminB1 and LaminB2 and measured Lamin enrichment at the NE for trunk tissues (Extended Data Fig. 6a–d). Early zebrafish embryos do not express LaminA/C, except for a small subset of somite cells^63^ (Extended Data Fig. 6a, pink arrows and Extended Data Fig. 6b). LaminB1 is expressed at low levels in the central nervous system (CNS) and in NCs (Extended Data Fig. 6a,c), but is more highly expressed in somites (Extended Data Fig. 6a,c). We observed LaminB2 to be expressed across all tissues and to be the most enriched lamin in tNCs (Extended Data Fig. 6a,d). Quantification of LaminB2 across NC subpopulations reveals that tNCs, but not cNCs or atNCs, significantly reduce LaminB2 immunoreactivity upon confined migration (Fig. 6a,b). This reduction in LaminB2 indicates that the neural crest nuclear lamina may adapt under confinement. LaminB2 is required for migration of mouse CNS neurons^64^, which traverse dense tissues^65^, and loss of LaminB2 abrogates NE rupture in melanoma^66^. Accordingly, we investigated the role of LaminB2 in zebrafish tNC confined migration.

**Extended Data Fig. 7:**
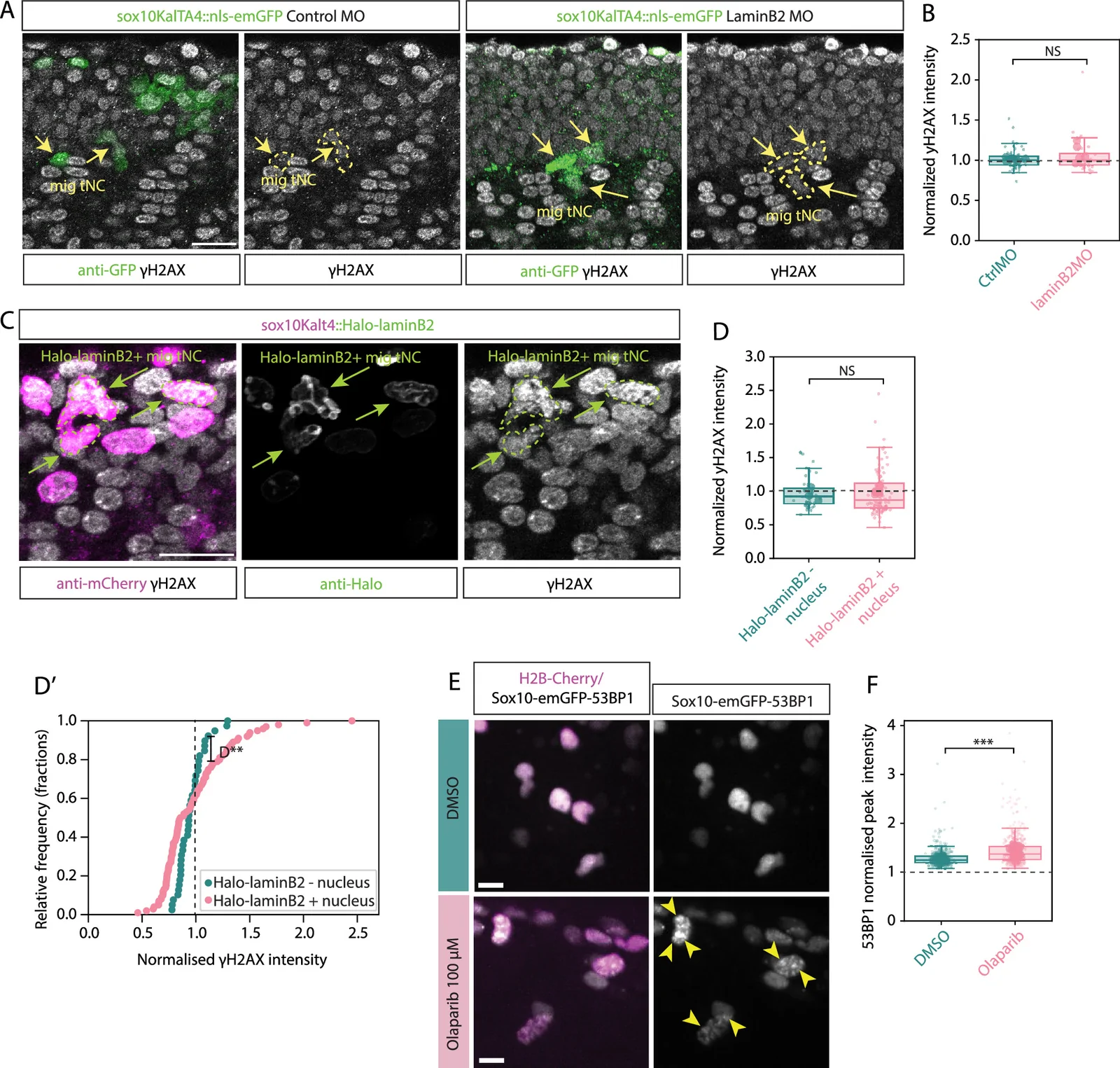
γH2AX immunostaining in LaminB2 MO and LaminB2 neural crest-specific overexpression. **A)** Representative image of single z-slice γH2AX immunostainings for sox10KalTA4:nlsGFP in CtrlMO or in laminB2MO injected embryos. Yellow arrows and outlines indicate migratory mid trunk NCs. Overlay: anti-GFP (neural crest), γH2AX. **B)** yH2AX intensity in migratory NC cells normalised to surrounding tissues in CtrlMO and laminB2MO injected embryos in mid trunk region. CtrlMO n = 6 embryos (63 cells), laminB2MO n = 5 embryos (37 cells). Mann-Whitney test: p = 0.1775. Small dots represent a single nucleus, large dots represent a mean value per embryo. **C)** Representative image of single z-slice anti Halo and γH2AX immunostainings for sox10Kalt4:UAS-Halo-LaminB2 embryos. Green arrows and outline indicate migratory Halo-LaminB2^+^ mid trunk NCs. Overlay: anti-mCherry (neural crest), γH2AX. **D)** yH2AX intensity in migratory NC cells normalised to surrounding tissues in Halo-laminB2 negative and positive cells in mid trunk region. Halo-laminB2 negative n = 3 embryos (49 cells), Halo-laminB2 positive n = 6 embryos (100 cells). Mann-Whitney test: p = 0.3810. Small dots represent a single nucleus, large dots represent a mean value per embryo. **D’)** Cumulative frequency histogram of yH2AX normalised intensity values for Halo-LaminB2^+^ and control Halo-LaminB2^−^cells. Kolmogorov-Smirnov test: p = 0.0012, D = 0.3631. **E)** Representative images of 53BP1 accumulation in nucleus after laparib treatment in NC cells in midtrunk region in Sox10-emGFP-53BP1 embryos. Yellow arrowheads highlight the localization of 53BP1 foci. **F)** 53BP1 normalised peak intensity in NC cells in olaparib treated and DMSO control cells. DMSO n = 7 embryos (564 cells, laparib n = 13 embryos (455 cells). Mann-Whitney test p = 0.0008. Small dots represent a single nucleus, large dots represent a mean value per embryo. Box plots show the median, the 1st and 3rd quartile and the 1.5x inter-quartile range. All statistical tests 2-sided. Scalebars 20μm (A, C) and 10μm (E). Source data

We carried out LaminB2 knockdown in zebrafish embryos using a translation-blocking morpholino (MO) oligonucleotide. Quantification of endogenous LaminB2 in control versus LaminB2 MO embryos showed efficient knockdown (77%, Extended Data Fig. 6e,f). LaminB2 knockdown affects the zebrafish head, eye and body axis (Extended Data Fig. 6g). We also noted a mild defect in melanophore distribution, which might indicate a neural crest defect (Extended Data Fig. 6g). LaminB2 knockdown in sox10KalTA4::nlsGFP embryos revealed that tNC migratory streams appeared shorter than control in LaminB2 MO embryos (Fig. 6c and Supplementary Video 14), further suggesting a defect in tNC migration. To ask how LaminB2 contributes to the nucleus deformation dynamics of migrating mid tNCs, we quantified nucleus shape changes. We observed that LaminB2 knockdown does not significantly affect the mean nuclear circularity of mid tNCs (Fig. 6d,e), but leads to a slight decrease in the duration of nuclear deformation events (Fig. 6f). We asked whether faster deformation dynamics could result in increased NE ruptures or DNA damage in LaminB2 MO embryos. However, we observed that neither tNC nls-emGFP nucleo-cytoplasmic leakage nor DNA damage are affected by LaminB2 knockdown (Extended Data Figs. 6i,j and 7a,b and Supplementary Video 14). Thus, downregulation of LaminB2 in migrating tNCs might support efficient nucleus deformation and recovery cycles, but LaminB2 knockdown does not compromise NE integrity, nor does it lead to DNA damage accumulation.

### Sustained expression of LaminB2 leads to highly deformed nuclei

Our observations show decreased endogenous LaminB2 in confined tNCs. To test whether sustained LaminB2 expression might affect the ability of tNCs to deform under confinement, we employed tissue-specific approaches for the overexpression of LaminB2. We generated a UAS-HaloTag-LaminB2 transgenic fish for the conditional expression of Halo-LaminB2. Neural-crest-specific expression of Halo-LaminB2 did not affect the overall embryo morphology, although it led to a mild melanophore defect at 2 dpf (days post fertilization) (Extended Data Fig. 6h). To investigate whether increased levels of LaminB2 impact nuclear shape changes and NE ruptures, we expressed Halo-LaminB2 in Sox10:KalTA4::nls-emGFP zebrafish. HaloTag-LaminB2 was detected mosaically at the NE of migrating tNCs (Fig. 6g and Supplementary Video 15). Quantification of mean nucleus circularity (Fig. 6h,i) highlighted that nuclei were significantly more deformed in Halo-LaminB2^+^ compared to Halo-LaminB2^−^ control tNC cells. Time-resolved curves of nuclear circularity showed that Halo-LaminB2^+^ nuclei remained deformed through time (Fig. 6i and Supplementary Video 15), leading to long-lasting deformations (Fig. 6j). Quantification of nls-emGFP nucleus intensity revealed that, despite increased nuclear deformations, sustained expression of Halo-LaminB2 did not further affect NE permeability (Extended Data Fig. 6k,l).

### Sustained expression of LaminB2 correlates with mild DNA damage in confinement

**Fig. 7:**
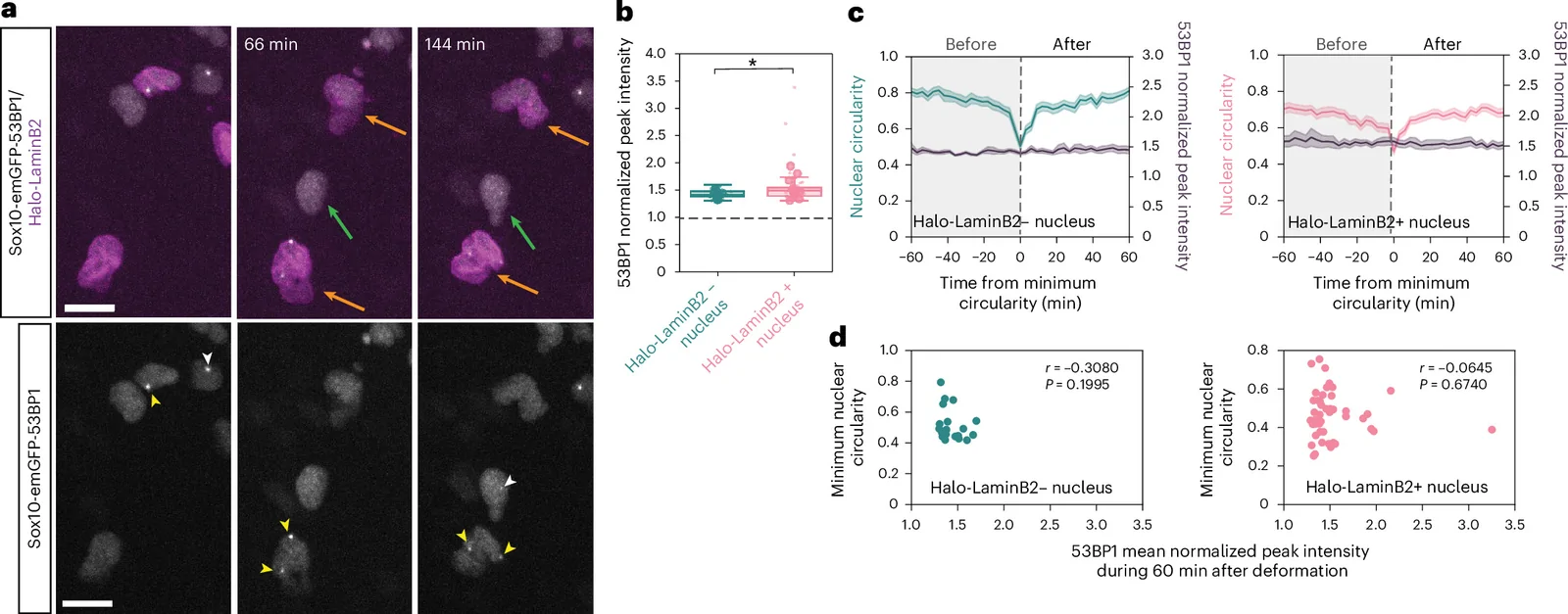
Sustained expression of LaminB2 correlates with mild DNA damage in confinement. **a**, Representative time series of the maximum projection of Halo-LaminB2-positive (magenta) and emGFP-53BP1 (grey) double-positive migratory NC cells in the mid trunk region. Orange arrows indicate Halo-LaminB2-positive cells; green arrows indicate Halo-LaminB2-negative cells. Arrowheads show 53BP1 foci in Halo-laminB2-positive cells (yellow) and -negative cells (white). **b**, Mean 53BP1 normalized peak intensity per nucleus in Halo-laminB2-negative and -positive migratory NC cells in the mid trunk region. Halo-LaminB2-negative cells *n* = 23 cells, nine embryos; Halo-LaminB2-positive cells *n* = 49 cells, 14 embryos. Mann–Whitney test, *P = 0.0272, Cohen’s *d* effect size, 0.46268. Small dots represent a mean normalized 53BP1 value of a single nucleus over time; large dots represent mean values per embryo. The box plots show the median, the first and third quartiles and the 1.5× interquartile range. **c**, Nuclear circularity and normalized 53BP1 peak intensity aligned to the minimum circularity upon NC migration in Halo-laminB2-negative (left) and -positive (right) cells. Halo-LaminB2-negative cells = 20 cells, nine embryos; Halo-LaminB2-positive cells *n* = 46 cells, 14 embryos. Data are presented as mean and s.e.m. Dashed lines separate the time before (grey) and after maximum deformation. **d**, Scatter plots showing the correlation of minimum nuclear circularity value and the mean value of normalized 53BP1 peak intensity up to 60 min after the maximum deformation event in Halo-LaminB2-negative (left) and -positive (right) cells. Halo-LaminB2-negative *n* = 19 cells, nine embryos; Halo-LaminB2-positive *n* = 45 cells, 14 embryos. The Pearson correlation test (Halo-LaminB2-negative) and Spearman correlation test (Halo-LaminB2-positive) were applied; *r* and *P* values are indicated in each scatter plot. All statistical tests are two-sided. Scale bars, 10 μm (**a**). Source data

We next asked whether sustained expression of Halo-LaminB2 in confined tNCs could result in DNA damage. Analysis of endogenous γH2AX fluorescence intensity did not show a notable increase in Halo-LaminB2^+^ nuclei compared to Halo-LaminB2^−^ control tNC cells (Extended Data Fig. 7c,d). However, the cumulative distribution of γH2AX intensity datapoints was significantly different in Halo-LaminB2^+^ cells (Extended Data Fig. 7d’, Kolmogorov–Smirnov test, **P < 0.01). To further investigate in live embryos whether the sustained expression of Halo-LaminB2 could result in increased DNA damage, we generated a neural-crest-specific sox10:emGFP-53BP1^trunc^ transgenic reporter line (emGFP-53BP1). Similar to the case with the mApple-53BP1 mRNA reporter, we readily detected 53BP1 foci upon olaparib treatment (Extended Data Fig. 7e,f). To investigate whether Halo-LaminB2 expression could result in increased DNA damage, we generated a sox10Kalt4 (ref. ^67^); sox10:emGFP-53BP1 double transgenic. To obtain conditional expression of Halo-LaminB2, we outcrossed these fish to UAS-Halo-LaminB2 fish and investigated 53BP1 dynamics in triple-positive sox10Kalt4, sox10:emGFP-53BP1, UAS-Halo-LaminB2 embryos in mid tNCs (Supplementary Video 16). EmGFP-53BP1 mean peak intensity in Halo-LaminB2^+^ versus Halo-LaminB2^−^ control cells was slightly, but significantly increased (Supplementary Video 16, Fig. 7a, orange arrows, and Fig. 7b). However, time-resolved analysis of 53BP1 peak intensity before and after maximum nuclear deformation did not specifically show a notable increase following deformation (Fig. 7c). Furthermore, we did not detect a correlation between minimum nuclear circularity and 53BP1 normalized peak intensity for up to 60 min after deformation (Fig. 7d). Our results suggest that although persistent expression of Halo-LaminB2 in migratory tNCs results in a mild increase in overall 53BP1 intensity, the small difference to the control cells suggest that other mechanisms might contribute to dampening DNA damage in migrating tNCs.

### tNCs upregulate the DNA damage response under in vivo confinement

**Extended Data Fig. 8:**
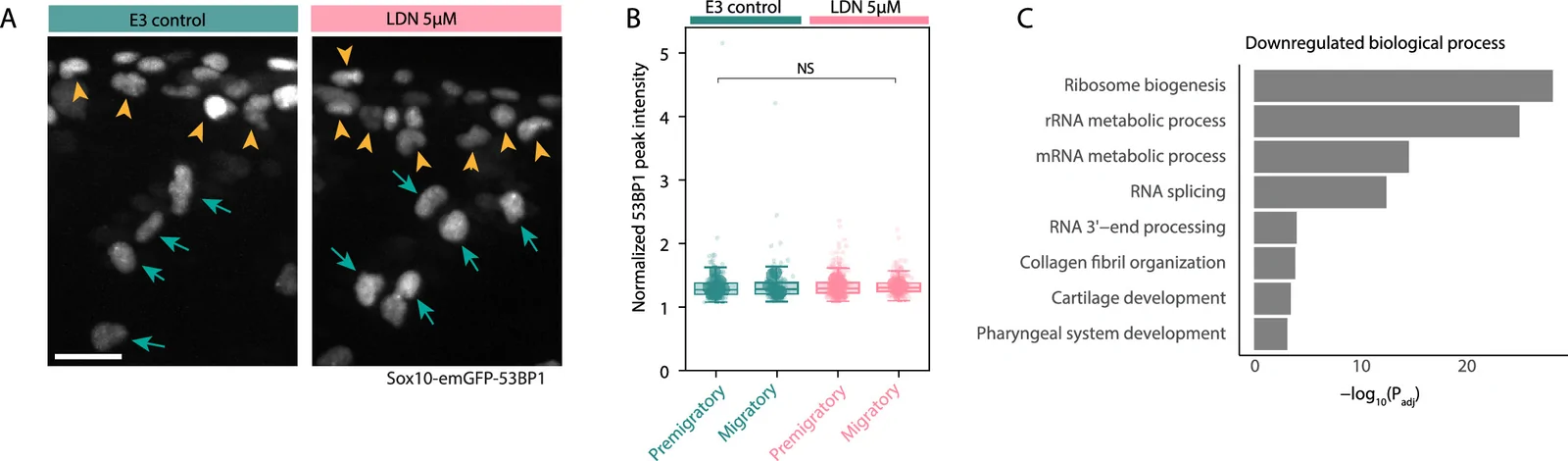
BMP and ATR inhibition do not affect DNA damage in neural crest cells; differentially downregulated biological processes at the endpoint of in vivo confined migration. **A)** Representative image of mid trunk NC streams in Sox10-emGFP-53BP1 embryos in 5uM LDN after 6 hours incubation. Green arrows show the migratory cells, orange arrowheads show the premigratory cells. **B)** Quantification of normalised 53BP1 peak intensity in premigratory and migratory cells in control embryos (n = 8 embryos, 333 premigratory cells, 177 migratory cells), and in VE821 treated embryos (n = 8 embryos, 351 premigratory cell, 169 migratory cells). Kruskal Wallis Dunn’s multiple comparisons test: p = 0.5216. Small dots represent a single nucleus, large dots represent a mean value per embryo. Box plots show the median, the 1st and 3rd quartile and the 1.5x inter-quartile range. **C)** Plot of the eight most downregulated biological processes in migratory tNCs. Adjusted P-values were calculated and adjusted for multiple comparisons using DESeq2 (see Methods). Scale bar 20 μm (A). Source data

How do NCs protect their nucleus from mechanical stresses? Recent work has identified a link between bone morphogenetic protein (BMP) signalling and DNA damage protection during zebrafish heart regeneration^68^ and in zebrafish primordial germ cells^69^. BMP signalling is active throughout the lifetime of NCs^70^ and governs the initiation of tNC migration by controlling the G1/S transition^71^. To test whether BMP signalling might protect migrating tNCs from DNA damage during confined cell migration, we inhibited BMP signalling, using a chemical inhibitor previously validated in zebrafish^68,69^. However, we found that inhibiting BMP did not affect the accumulation of emGFP-53BP1 in live embryos (Extended Data Fig. 8a,b).

**Fig. 8:**
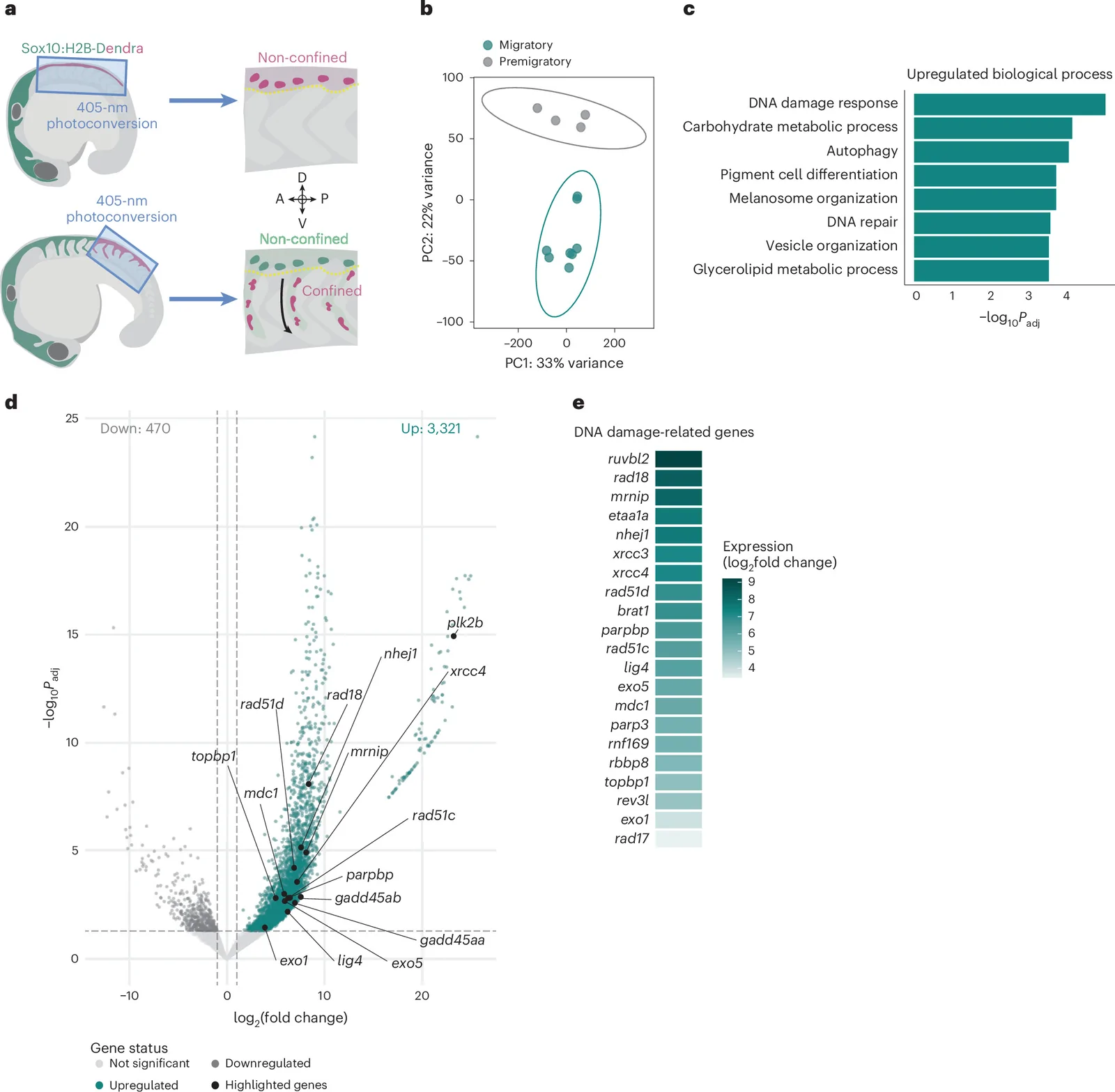
Trunk neural crest upregulates the DNA damage response at the endpoint of in vivo confined migration. **a**, Outline of the photoconversion and bulk RNA-seq experiment (Methods): sox10-H2B-Dendra2 premigratory tNC nuclei were photoconverted at 16 hpf using a 405-nm laser as non-confined control (top). Migratory streams from somites 10–15 (mid trunk) were photoconverted at 23–24 hpf using a 405-nm laser (bottom). **b**, PCA analysis of premigratory versus migratory tNC photoconverted samples; premigratory *n* = 4 biological replicates, migratory *n* = 8 biological replicates. **c**, Plot of eight of the most upregulated biological processes in migratory tNCs. **d**, Volcano plot of upregulated versus downregulated genes in migratory tNCs. A subset of upregulated genes involved in the DNA damage response are highlighted. In **c** and **d**, adjusted *P* values were calculated and adjusted for multiple comparisons using DESeq2 (Methods). **e**, Heatmap of a subset of upregulated DNA damage response genes in the migratory tNC transcriptome (log_2_fold change). Source data

To gain insight into how migrating tNCs may counteract DNA damage when migrating in confinement, we carried out RNA sequencing (RNA-seq). Using a neural-crest-specific photoconvertible transgenic line (Sox10:H2B-Dendra^67^), we photoconverted mid-trunk neural crest nuclei before confined migration (16 hpf) or at the endpoint of confined migration (23–24 hpf) and carried out low-input bulk RNA-seq (*n* = 20 cells per sample) (Fig. 8a). Principal component analysis (PCA) revealed that migration through confinement alters the gene expression of tNCs (Fig. 8b). Strikingly, the most upregulated biological process in confined tNCs is the DNA damage response (70 genes; Fig. 8c and Extended Data Fig. 8c). Analysis of this gene group reveals that components of several pathways involved in the repair of DNA double-strand breaks are upregulated at the endpoint of confined tNC migration, including homologous recombination, non-homologous end joining and checkpoint signalling (Fig. 8d,e), thus suggesting that this stem cell population is probably endowed with a complex program enabling cells to cross unscathed through confining tissue environments.

## Discussion

We have established the zebrafish neural crest as an in vivo model to investigate the cellular responses of a multipotent stem-cell population to physical confinement in a physiological in vivo context. Our findings reveal that, during their developmental migration, NCs invade highly confining inter-tissue spaces across the vertebrate body axis. This results in repeated nuclear deformation events characterized by striking nucleus shape changes that initiate as a transition of tNCs from the dorsal premigratory area to the narrow inter-tissue space under the somite. Tissue-scale confinement and col1a1a density increase with developmental time along the AP body axis, and this quantitatively scales with the extent of NCs’ nuclear deformation. In contrast to tNCs, cNCs and atNC of the vagal streams, which encounter a lesser degree of confinement, do not show notable nuclear deformation. Using a nuclear-localized emGFP, we observe nucleo-cytoplasmic leakage in migrating tNCs, but not premigratory tNCs or unconfined migrating cNCs. However, live imaging of two NE rupture reporters, BAF-mCherry and cGAS-EGFP, did not show evidence of NE rupturing. Using SEM microscopy, we detected intact NEs in migrating tNCs. The wide distribution of nuclear pore widths measured in SEM datasets suggests that nucleo-cytoplasmic leakage of nls-emGFP might be linked to dynamic stretching of nuclear pores (Fig. 4h,i). Indeed, mechanical stress can deform the nucleus^50^, leading to an expansion in nuclear pore diameter^72^, which can result in passive leakage of low-molecular-weight proteins such as nls-emGFP across the nuclear pore^50,51^. Future studies addressing active nuclear transport using live imaging methods^51^ or correlative light-electron microscopy (CLEM), where live imaging is paired with high-resolution transmission electron microscopy (TEM) imaging, will be required to validate the physical dimensions of the nuclear pores in migrating tNC cells showing transiently deformed nuclei. Work in migrating *Drosophila* immune cells^17^ also found that only a small fraction of cells show NE ruptures after a significant nuclear deformation in vivo. Importantly, in this and other in vivo models, such as in *Caenorhabditis* *elegans* oocyte development^73^, regulation of the LaminB orthologues was found to be essential to in vivo NE integrity.

### Modulation of LaminB2 and its functional implications

We observed that endogenous LaminB2 immunoreactivity consistently decreases in confined migratory NC populations, whereas it remains unchanged in non-confined NC cells. This could reflect a true downregulation of LaminB2 protein levels or masking of a mechanosensitive epitope in the Ig-fold domain^74^, although this has only been reported for LaminA/C^75^. Alternatively, downregulation of LaminB2 might be due to reduced protein translation in migratory tNCs. Indeed, our RNA-seq data highlight that mRNA processing and translation might be inhibited in migratory tNCs (Extended Data Fig. 8b), whereas we find that LaminB2 mRNA is not differentially expressed between premigratory and migratory mid tNC cells.

Downregulation of LaminB2 might prove advantageous for tNC confined migration. Indeed, LaminB2 knockdown mildly decreased the duration of nucleus deformations, which suggests decreased nuclear elasticity. Conversely, sustained expression of Halo-LaminB2 resulted in continuously deformed nuclei. Thus, LaminB2 modulation may soften nuclei as they encounter a confining environment. In line with this hypothesis, atomic force microscopy (AFM) measurements of cytoplasm stiffness in LaminB2 knockout fibroblasts reveal marked softening and an increased capability for confined cell migration^76^. Moreover, *Xenopus* neural crest become softer as they transition from a premigratory to migratory state^77^, so fine-tuning of LaminB2 levels could allow the nucleus to match the rheological properties of surrounding tissues to facilitate passing narrow constrictions, minimizing nuclear mechanical stress. Mouse LaminB2 knockout neurons show mild NE rupture and repair defects in vitro^78^. On the other hand, knockdown of LaminB2 in melanoma^66^ abrogates NE permeability and NE ruptures under confinement. LaminB2 knockdown or overexpression did not affect NE permeability in migrating tNCs. This suggests that LaminB2 might play a context-dependent role in NE integrity, depending on the relative abundance of other Lamins and of other molecular partners such as NE tethers^66^. Our results indicate that sustained expression of LaminB2 results in a mild increase in overall 53BP1 peak intensity in live tNCs. However, we could not find a correlation between increased nuclear deformations and 53BP1 peak intensity in Halo-laminB2^+^ cells. Instead, we find that even under sustained expression of Halo-LaminB2, confined tNCs still maintained their overall levels of DNA damage at low levels.

### Regulation of DNA damage pathways in tNCs

Neural crest are long-lived stem cells that, upon completion of their migration, give rise to a plethora of cell types. NCs colonize many different tissue niches within the embryo, and their adaptability is considered instrumental to the success and diversification of vertebrates^79–83^. Thus, this cell type might be endowed with robust mechanisms to withstand stressors encountered during their developmental journey. Indeed, in striking contrast with other instances of in vitro^10–12,14,16^ and in vivo confined cell migration^17–19,84^, we find that NCs do not accumulate DNA damage when migrating through confined tissue environments. Instead, we report that in vivo levels of DNA damage, both endogenous (Fig. 5a,b) and measured using a live reporter for DNA double-strand breaks (Fig. 5c,d), are unchanged, or even reduced in the most confined posttNC migratory population. Using the photoconversion of mid tNCs before and at the endpoint of confined migration as well as low-input bulk RNA-seq, we discovered that tNCs upregulate a large number of genes involved in the DNA damage response and DNA repair (Fig. 8a,e). This program appears robust, as numerous genes involved in non-redundant pathways for the repair of DNA double-strand breaks are similarly upregulated (Fig. 8d,e). Further investigation is required to understand how cells might be sensing confinement to upregulate the DNA damage response. To investigate in detail the molecular and cellular mechanisms involved, future studies might employ pharmacological and genetic approaches to inhibit nuclear mechanosensing pathways in vivo and in vitro. Furthermore, functional genetics strategies coupled to high-resolution live imaging of 53BP1 will help to pinpoint the requirement for specific components of the upregulated DNA damage repair pathways in the future.

Why would tNCs be endowed with such protection mechanisms? Failure to mount a protective response to migration through confining environments might have deleterious consequences for tNCs, as it might lead to developmental defects^19^, the accumulation of mutations, and tumour initiation. Human cancers such as melanoma and neuroblastoma are tNC-derived^85,86^. Polymorphisms predisposing to neuroblastoma occur in DNA repair genes and cell-cycle checkpoint pathways^87^. Growing clinical evidence shows that neuroblastomas originate in utero, at early stages of tNC development^88^, strongly suggesting that chromosomal instability occurs early in neuroblastoma tumorigenesis and that human tNCs may suffer inherent genomic instability^87,89^. Clinical studies^90^ have also shown that, depending on anatomical location, human neuroblastomas carry different types of chromosomal aberration. Of note, P53 zebrafish mutants spontaneously develop tNC-derived malignant peripheral nerve sheath tumours^91,92^. Thus, under unfavourable genetic circumstances, the physical environment of tNCs may influence neuroblastoma pathogenesis. Mechanical stresses are also increasingly recognized as pathogenic in acral melanoma^93,94^. Melanomas often hijack embryonic NC states^86,95^, and re-activation of embryonic adaptability programs could also contribute to aggressiveness. Importantly, nuclear adaptation to migration in confined environments by the uncoupling of the inner nuclear membrane and lamina via Lap1c was recently reported in human metastatic melanoma^3^. In summary, our work provides insight into how an evolutionarily successful, multipotent cell type, the neural crest, adapts to challenging extracellular environments in a physiological tissue context by modulating NE composition, limiting accumulation of DNA damage, and thus ensuring robust developmental migration in the early embryo.

## Methods

### Ethics

Zebrafish work was approved by the University of Cambridge Animal Welfare and Ethical Review Board (AWERB). Work was carried out under the United Kingdom Animal (Scientific Procedures) Act 1986 legislation under Home Office Project Licence PP8606293 to E.S.

### Zebrafish maintenance

Zebrafish were maintained and raised at 28 °C on a 10-h/14-h dark/light cycle according to standard conditions^96,97^. Adult fish were used exclusively for breeding, and all experiments were carried out on unregulated embryos younger than five days post fertilization. Embryos were bred from breeding tanks where three or four females were crossed to two or three males. As zebrafish fertilization occurs in water, each embryo in such clutches might have a different set of parents and hence each embryo is considered to be an independent experiment. Embryos were kept in 1X E3 medium (60X solution: 17.2 g of NaCl, 0.76 g of KCl, 2.9 g of CaCl_2_·2H_2_O, 4.9 g of MgSO_4_·7H_2_O). The temperature was kept at 28 °C until the shield stage, then adjusted between 22 °C and 28 °C depending on the developmental stage at which the embryos were to be imaged. Before experiments, embryos were staged based on morphological criteria such as the number of somites and elongation of the tail.

### Zebrafish transgenic and mutant lines

The following zebrafish lines were used in this study: WT (AB and Tüpfel long fin (TLF)), Sox10:mG^33^, Sox10-Kalt4^52^, sox10-H2B-Dendra^52^, Sox10::KaltA4;UAS-nls-emGFP^47^, *spt-1* (*b104r1*)^39^, Sox10: emGFP-53BP1trunc, UAS-cGASa-EGFP and UAS-Halo-laminB2. To generate the UAS-nls-emGFP transgenic line, 20 pg of pTol1-UAS-nls-emGFP cmlc2EGFP plasmid DNA (14xUAS-E1b:BGi-nls-emGFP clmc2-eGFP)^98^ was injected with 80 pg of Tol1 transposase mRNA at the 1-cell stage. F1 founders were identified by screening for green heart fluorescence. To generate the UAS-cGASa-EGFP transgenic line, 20 pg of pTol1-UAS-nls-emGFP cmlc2EGFP plasmid DNA was injected with 80 pg of Tol1 transposase mRNA at the 1-cell stage. F1 founders were identified by screening for green heart fluorescence. To generate the Sox10: emGFP-53BP1trunc transgenic line, 5–10 pg of plasmid DNA was co-injected with 80 pg of Tol1 transposase mRNA into 1-cell-stage zebrafish embryos. F1 founders were identified based on green heart fluorescence. To generate the UAS-Halo-LaminB2 transgenic line, 15 pg of plasmid DNA was injected with 80 pg of Tol1 transposase mRNA at the 1-cell stage. F1 founders were identified by screening for green heart fluorescence.

### Genotyping

*spt-1* (*b104r1*) heterozygous fish^39^ were crossed into sox10:mG background and Sox10mG. The primer pair sptF (5′-TTG ACC ACA ATC CCT TTG CCA A-3′) and sptb104R (5′-GCC TTC ACC TCC AGC TCT TTA CG-3′), flanking the deletion found in sptb104 mutants, was used. These primers amplified approximately 0.9-kb and 1.9-kb products in the mutant and WT, respectively, and were used for genotyping^99^. Double heterozygous fish were incrossed to generate *spt* mutant and control siblings.

### Molecular cloning

mApple-53BP1 and BAF-mCherry were subcloned into a pCS2^+^ vector for mRNA synthesis from Addgene plasmids 69531 ref. ^58^ and 172447 (ref. ^100^). For mApple-53BP1, pCS2^+^ was digested with BamHI and EcoRI (New England Biolabs), and mApple-53BP1 was amplified with the following primers: 5′-CATCATGGATCCatggtgagcaagggcgag-3′ and 5′-GAGGAGGAATTCctacccggtagaattatc-3′. Fragments were ligated using a T4 DNA ligase (Promega) and transformed into competent cells. For BAF-mCherry, pCS2^+^ was linearized with EcoRI (New England Biolabs). BAF-mCherry was amplified by polymerase chain reaction (PCR) using the primers 5′-AGGATCCCATCGATTCGAATTCatggtgagcaagggcgaggag-3′ and 5′-GCTCGAGAGGCCTTGAATTCctacaagaaggcatcacaccactctcgaa-3′, and subcloned in pCS2 using the NEB Builder HiFi Kit (New England Biolabs). The cmlc2EGFP expression cassette was amplified from pDestTol2CG2 (refs. ^101,102^) using the primers 5′-CATCATGGTACCAAAGCTTAAATCAGTTGT-3′ and 5′-ATGATGGGTACCTTACTTGTACAGCTCGTC-3′, and inserted into the Kpn I site of 14xUAS-BGI-nucEmGFP-POUT using T4 DNA ligase (Promega). To generate the UAS-cGASa-EGFP plasmid, Danio Rerio cGASa was amplified by PCR from pEGFPN1-cGASa-EGFP (ref. ^103^) using the primers 5′-CTGGGCAACGTGGAATTCGATATCATGAGCAGCCACAGGAGACCAG-3′ and 5′- GGTGGCTGAGACTTAATTACTAGTTTACTTGTACAGCTCGTCCATGCC-3′, then inserted into a pTol1-UAS-nls-emGFP plasmid backbone linearized with EcoRV-SpeI to remove the nls-emGFP insert using the NEBuilder HiFi DNA Assembly Cloning Kit (New England Biolabs, 10173994). To generate the UAS-Halo-LaminB2 plasmid, Halo was amplified by PCR from LZ10 PBREBAC-H2BHalo (Addgene 91564) using the primers 5′-CTGGGCAACGTGGAATTCGATATCATGGAAATCGGTACTGGCTTT-3′ and 5′-TCGGGGTTGCGGTCGCGATATCACCGGAAATCTCCAGAGT-3′. Danio Rerio LaminB2 (ref. ^104^) was amplified using the primers 5′-ACTCTGGAGATTTCCGGTGATATCGCGACCGCAACCCCGA-3′ and 5′-GGTGGCTGAGACTTAATTACTAGTTCACATCACTGCACATTCTCTGG-3′.

The Halo-laminB2 fusion was assembled into a pTol1-UAS-nls-emGFP plasmid backbone previously linearized with EcoRV-SpeI to remove the nls-emGFP insert using the NEBuilder HiFi DNA Assembly Cloning Kit (New England Biolabs, 10173994). DNA damage in vivo was detected using a newly generated zebrafish transgenic line expressing 53BP1trunc^58^ as a reporter under control of the Sox10 promoter, thereby restricting expression to NCs. To improve brightness and enhance the detection of 53BP1 foci, the fluorescent protein mApple was replaced with emGFP. emGFP was amplified from the pTol1-UAS-nls-emGFP cmlc2EGFP plasmid. Cloning was performed as follows. First, the Sox10-4.9-kb promoter^105^ was cloned into the pTol1-UAS-nls-emGFP plasmid backbone. The plasmid backbone was linearized by digestion with StuI-HF (New England Biolabs) and the UAS-nlsGFP sequence excised. The Sox10-4.9-kb promoter was amplified using the primers 5′-CTGAACCTGGATCAGCTTAGGCCTCCCTTATCAGAGTCAACATTCATG-3′ and 5′-GTAGAAGAGAACCCAGGACCTGAATTCGATATCGCCACCATG-3′, and assembled into the backbone using the NEBuilder HiFi DNA Assembly Cloning Kit (New England Biolabs, 10173994). To insert 53BP1-emGFP, the pTol1-Sox10-4.9 plasmid was then linearized with SpeI. emGFP and 53BP1 were amplified with the following primers and the sox10-4.9kb-emGFP-53BP1 plasmid assembled using the NEBuilder HiFi DNA Assembly Cloning Kit (New England Biolabs, 10173994): for emGFP, 5′-ACCTGTGGCCGCAGAACTAGTATGGTCTCAAAGGGAGAGGAACTG-3′ and 5′-AGGCATATCAAACTCTTCTTCTCCCTTGTACAGCTCATCCATTCCATG-3′; for 53BP1, 5′-CATGGAATGGATGAGCTGTACAAGGGAGAAGAAGAGTTTGATATGCCT-3′ and 5′-GGTGGCTGAGACTTAATTACTAGTCTACCCGGTAGAATTATCTAGATC-3′.

### mRNA synthesis and microinjections

mRNAs were synthesized from linearized pCS2^+^ plasmids using the SP6 mMessege mMachine kit (Invitrogen). mRNAs were injected as described in the following subsections.

#### Global labelling of tissues

Membrane GFP, membrane Cherry (mCherry)^106^ and Neurocan-GFP (ss-N-Can-GFP)^37^ were injected at the 1-cell stage directly to the cell at concentrations of 20–50 pg per embryo, 50 pg per embryo and 25 pg per embryo, respectively, in WT or the Sox10mG background. Embryos with the most homogenous signal were selected for experiments.

#### Mosaic labelling of single cells

mApple-53BP1 trunc and BAF-mCherry mRNAs were injected into a single cell at the 4- to 16-cell stage for mosaic expression into the Sox10::Kalta4;UAS-nls-emGFP background. To visualize 53BP1 and BAF dynamics in the NC population specifically, embryos with double expression of Sox10::Kalta4;UAS-nls-emGFP and injected mRNA were selected for mounting. Final selections of embryos with cellular co-localization of injected mRNA and Sox10::Kalta4;UAS-nls-emGFP were done at the microscope. For the measurement of nucleo-cytoplasmic leakage in collectively migrating cNCs, Sox10::Kalta4;UAS-nls-emGFP embryos were injected at 1-cell stage with 1 nl of Sox10-lynTomato plasmid DNA^107^ at a concentration of 6 pg per embryo together with 30 pg per embryo g Tol2 transposase mRNA.

#### Morpholino injections

Embryos were microinjected at the 1-cell stage into the yolk with translation-blocking morpholinos antisense oligonucleotides (Gene Tools). The following morpholinos (MOs) were used: LaminB2 MO, ‘TTATGATCGCTTCAGACCCTCTTCT’; standard control MO, ‘CCTCTTACCTGAGTTACATTTATA’. Both morpholinos were used at a concentration of 4 ng per embryo.

### Tissue-specific overexpression of LaminB2

A mosaic pattern of Halo-laminB2 overexpression for in vivo live imaging was achieved by microinjection of 15 pg of UAS-Halo-laminB2 plasmid together with 80 pg per embryo g Tol1 transposase mRNA in Sox10::Kalta4;UAS-nls-emGFP embryos at the 1-cell stage, or by outcrossing UAS-Halo-laminB2-positive fish with Sox10::Kalta4;UAS-nls-emGFP. To label the UAS-Halo-laminB2-positive cells, embryos were dechorionated at the dome stage, and incubated with 0.1 μM Halo-ligand JF546 (GA1110, Promega) or Halo-ligand JF646 (GA1120, Promega) in 0.3X Danieau’s solution (30X solution 50.5 g NaCl, 0.78 g KCl, 1.48 g MgSO_4_·7H_2_O, 2.125 g Ca(NO_3_)_2_·4H_2_O, 17.8 g HEPES, pH 7.6). Before mounting (see ‘Mounting of embryos for in vivo imaging’), positive embryos were screened based on the double fluorescent signal, and the embryos were washed with E3 medium to remove excess ligand. For 53BP1 and Halo-laminB2 double fluorescent in vivo live imaging, UAS-Halo-laminB2 fish were outcrossed with sox10Kalt4;sox10-emGFP-53BP1, the embryos were dechorionated at the dome stage and incubated with 0.1 μM Halo-ligand JF646 (GA1120, Promega) and 1:20,000 tamoxifen to drive the overexpression in 0.3X Danieau’s solution. For Halo-laminB2 overexpression in fixed samples, UAS-Halo-laminB2 fish were outcrossed with Sox10Kalt4, and treated with 1:10,000 tamoxifen to induce expression. For all LaminB2 overexpression experiments, the final selection of embryos for timelapse imaging was done at the microscope based on the mosaicism of Halo-laminB2-positive cells.

### Phenotype characterization

Representative images of Halo-laminB2 overexpression and laminB2 MO were captured at 2 dpf. For laminB2 MO, WT (TLF) embryos were injected with morpholinos (see ‘Morpholino injections’). For Halo-laminB2 overexpression, UAS-Halo-laminB2 fish were outcrossed with Sox10ind-Kalt4 embryos and treated with 1:10,000 tamoxifen from the shield stage (6 hpf) onwards. Positive and negative embryos for UAS-Halo-laminB2 were selected by green heart fluorescence at 24 hpf from the same dish. The embryos were mounted, and single images were captured using a Leica M205 FA fluorescence microscope.

### Visualization of the space

To visualize the extracellular space between adjacent tissues in live embryos, Neurocan-GFP mRNA injection or 10-kDa dextran red or far red was used (Alexa 546 10,000 M_w_ dextran, Invitrogen D22911, Alexa 647 10,000 *M*_w_ dextran, Invitrogen D22914). Dextran red was used for space quantification at a concentration of 0.2 ng per embryo, and far red was used for live imaging at a concentration of 0.3–0.6 ng per embryo. To visualize the extracellular space in the cranial region NCan-GFP mRNA was injected at the 1-cell stage (single-timepoint imaging for space quantification), or dextran-647 was injected at the 1 K blastula stage into the extracellular space (in vivo live imaging). To image the space in the trunk, embryos were injected with dextran shortly before experiments. The embryos were mounted laterally on a 60-mm plastic dish in single drops of 0.6% low-melting-point agarose (LMPA, Sigma A4018). To visualize the extracellular space in anterior and mid trunk, dextran was injected into the extracellular space between the yolk sac and EVL next to the head (Extended Data Fig. 2a). To visualize the space in the posterior trunk, dextran was injected into the extracellular space at the tip of the yolk extension. Differences in extracellular space labelling and injections are justified by the different developmental stages; at earlier stages, injections directly into the extracellular space between the yolk and EVL are difficult to achieve.

### Perturbation experiments

#### Laser ablation

To remove individual somites from the live embryo, presomitic mesoderm (psm) was ablated using a TriM Scope II Upright 2-photon scanning fluorescence microscope controlled by Imspector Pro software (LaVision Biotec) and equipped with a tunable near-infrared laser source delivering 120-fs pulses with a repetition rate of 80 MHz (Insight DeepSee, Spectra-Physics). The laser was set to 927 nm, with power ranging between 1.05 and 1.20 W. The maximum laser power reaching the sample was set to 220 mW, and an electro-optical modulator was used to allow microsecond switching between the imaging and treatment laser powers. Laser light was focused by a ×25, 1.05-numerical-aperture (NA) water immersion objective lens with a 2-mm working distance (XLPLN25XWMP2, Olympus). Ablations were carried out during image acquisition (with a dwell time of 9.27 µs per pixel), with the laser power switching between treatment and imaging powers as the laser scanned across the sample. Embryos were treated at around the 14- to 17-somite stage. mGFP-positive embryos were selected and mounted in 0.6% LMPA (in E3 medium) laterally on a 60-mm plastic dish in agarose wells (see ‘Mounting embryos for in vivo imaging’). First presomitic mesoderm (PSM) was detected by morphology, and somites already segmented from the PSM were not targeted. The region of interest (ROI) was selected by creating a ROI manually avoiding targeting neural tube or already segmented somites. The laser was set at 927 nm, and 85–100% of 1.0–1.2-W laser power was applied only to the selected ROI with a 2-µm *z*-interval for a few micrometres at a time. Several rounds of ablation were performed, and each round ROI was re-defined, securing an accurate targeting to the PSM tissue only. The deepest PSM tissue adjacent to the neural tube and notochord (likely representing adaxial cells) was left untreated to avoid tissue damage in the underlying structures (neural tube and notochord) and to provide guidance cues for migrating tNCs. After the ablation, the embryos were carefully removed from the agarose and left to develop at 28 °C for 2 h, then remounted laterally in 0.6% agarose for in vivo live imaging.

For the proof-of-concept experiments for the NE rupture reporters emGFP, BAF and cGas in vivo, a TriM Scope II upright 2-photon scanning fluorescence microscope and a Nikon AX R multiphoton microscope with tunable coherent chameleon vision laser and an axon 1024 fixed line laser were used. Sox10::Kalta4;UAS-nls-emGFP was injected with BAF-Cherry mRNA, and Sox10Kalt4::cGASa-EGFP embryos were mounted dorsally for live imaging (see ‘Mounting of embryos for in vivo imaging’). With the Nikon AX R multiphoton set-up, a CFI75 Apo 20XC W (NA 1) objective was used. Imaging was performed with a 920-nm laser line at 40% power with a 0.8-ms dwell time per pixel. A few micrometres at the NE were targeted, and the ablation was applied with 6-ms dwell time per pixel. For the TriM Scope II upright 2-photon scanning fluorescence microscope, a 920-nm laser line was used. Ablation of the NE was carried out using 85–100% of the 1.0–1.2-W power.

#### Spadetail mutant

Double heterozygous sox10mG;*spt104R* fish were incrossed. For measurements of the extracellular spaces, embryos were injected at the 1-cell stage with Ncan-GFP mRNA. For live imaging, embryos were injected at the 1,000-cell stage with 10-kDa dextran-647. Embryos positive for both sox10:mG and dextran were selected, and mounted for live imaging at 16 hpf as described in the following. *Spt* mutant embryos were morphologically identified by lack of trunk somites and by the spade-shaped tailbud. After imaging, control embryos were recovered and genotyped as previously described. Both heterozygous and *wt* siblings were included in the analysis as we did not detect differences in inter-tissue spaces or nuclear shape dynamics (data not shown).

#### Drug treatments

For the validation of 53BP1mApple mRNA, 40 pg of mRNA was injected at the 1-cell stage in WT embryos. Embryos were dechorionated at 6 hpf (for 53BP1 mRNA validation) and incubated for 3 h in olaparib (100 μM) or in dimethyl sulfoxide (DMSO; 1:500) in E3 medium, prior to imaging. For the validation of Sox10:emGFP-53BP1trunc transgenicity, embryos were dechorionated at 12 hpf and incubated in olaparib (100 μM) or in the corresponding DMSO (1:500) in E3 medium for 6 h before imaging.

For BMP inhibition, LDN193189 hydrochloride was used at a concentration of 5 μM. Embryos were dechorionated at 12 hpf and incubated in the drug 6 h before imaging.

### In vivo imaging

#### Mounting of embryos for in vivo imaging

Embryos were manually dechorionated using fine forceps and mounted on a 35-mm glass-bottomed dish (Ibidi, 81158) in 0.6% LMPA/E3 medium with 0.6% MS222 (Sigma A5040-100G). For imaging of the extracellular space, embryos were mounted dorsal side up on 60-mm plastic dishes in agarose wells. The wells were created prior to the experiments in 1% agarose in E3 medium with custom-made moulds with a well diameter of 500 µm. To image NC dynamics, the embryos were mounted laterally on a glass-bottomed 30-mm dish (Ibidi, 81158). To image the posterior trunk, agarose was removed around the tail so that it could freely elongate. The imaging dish was filled with E3 medium with 0.2–0.4% MS222 depending on the developmental stage.

#### Microscopy

The extracellular space in the cranial region was examined using a Nikon Eclipse E1000 instrument equipped with a spinning disc unit (Yokogawa CSU10), a laser module providing 491-nm and 561-nm excitation (Spectral Applied Research LMM2) and a C9100-13 EM-CCD (electron-multiplying charge-coupled device) camera (Hamamatsu). Image acquisition was carried out using the Volocity software (Perkin Elmer), using a Nikon FLUOR ×40/0.80 W DIC M WD 2.0 water immersion objective with a *z*-interval of 0.5 µm. Extracellular space in the anterior, mid and posterior trunk regions was imaged using two-photon fluorescence microscopy on a TrimScopeII multiphoton microscope, using a Ti:sapphire tunable laser at 927 nm for Ncan-GFP and a 1,040-nm fixed-wavelength laser for 10-kDa Rhodamine-dextran at 1.2 W at 10–17% laser power with a ×25, 1.05-NA water immersion objective lens with a 2-mm working distance (XLPLN25XWMP2, Olympus), using a *z*-interval of 0.5 µm.

For live imaging of NC dynamics, we used a Perkin Elmer Ultraview Vox spinning disc microscope, with a ×30 UPlanSApo Silicone immersion lens (Olympus) and a Nikon W1 SoRa set-up with ×25/1.05-NA, CFI Plan Apo Lambda S, 0.55-mm, silicone oil, MRD73250 objective. Imaging was carried out at 28 °C using a pre-equilibrated incubation chamber. With the Perkin Elmer Ultraview Vox spinning disc, 100-μm stacks with a *z*-interval of 1 μm were usually acquired every 3 min for 8 h with 15–22% laser power and 50- to 100-ms exposure time for the 488- and 561-nm channels, and 5–10% laser power and 20- to 50-ms exposure time for the 633-nm channel. For mApple-53BP1 trunc and BAF-mCherry experiments, live imaging was performed using a time resolution of 1 min. With the Nikon W1 SoRa set-up, 50- to 100-μm stacks with a *z*-interval of 1 μm were acquired every 1–3 min for 6–8 h with 2–5% laser power and 80- to 200-ms exposure time for the 488-nm channel, 2–10% laser power with 80- to 200-ms exposure time for 561 nm, and 1–5% laser power with 80- to 150-ms exposure time for 633 nm, depending on the experiment. Three-colour imaging for emGFP-53BP1, H2B-cherry and Halo-laminB2 was carried out with a dual camera.

NCs cultured in pillar devices were imaged using a CSU-W1 Nikon SoRa spinning disc confocal microscope equipped with an environmental chamber maintained at 28.5 °C and 5% CO_2_. Timelapse imaging was performed using a ×20 air objective (NA 0.80) with an additional ×2.8 optical zoom. Images were acquired in 16-bit dual-camera mode using 488-nm and 555-nm laser lines at 7% power with 150-ms exposure times. The *z*-stacks were collected with a 2-μm step size at a temporal resolution of 20 frames per hour over a total duration of 10 h.

### Immunostaining

Immunostaining for nuclear antigens was carried out by adapting a previously published deyolking protocol^108^ to improve antibody penetration. Briefly, embryos were dechorionated and fixed for 2 h with 1% paraformaldehyde (PFA) in phosphate-buffered saline (PBS), and washed three times with PBS 0.1% Triton-X-100. The yolk was punctured with a fine needle, and embryos were mechanically agitated with a glass Pasteur pipette to facilitate yolk detachment. Embryos were then postfixed with 4% PFA at room temperature, permeabilized with three PBS 0.5% Triton-X-100 washes, and blocked at room temperature with blocking buffer (PBS 0.5% Triton-X-100, 4% normal goat serum, 2% DMSO). Primary antibodies were incubated for 48 h at 4 °C, washed in PBS 0.5% Triton-X-100, and secondary antibodies were incubated for 2 h at room temperature in blocking buffer at a dilution of 1:500. The embryos were mounted on glass slides in 75% glycerol/PBS and imaged using either an Olympus FV3000 confocal microscope on a ×30 silicone immersion lens or a Leica SP8 confocal microscope using a ×40 oil immersion lens. For collagen1a1a immunostaining, embryos were dechorionated and fixed with 4% PFA overnight at 4 °C, washed and permeabilized with PBS 0.5% Triton-X-100, and blocked at room temperature with blocking buffer (PBS 0.5% Triton-X-100, 4% normal goat serum, 2% DMSO). Primary antibodies were incubated overnight at 4 °C in blocking buffer and washed with PBS 0.5% Triton-X-100, and secondary antibodies were incubated for 3 h at room temperature in blocking buffer, washed with PBS 0.5% Triton-X-100, then 4′,6-diamidino-2-phenylindole (DAPI) was added in the final round of washes at a concentration of 2.5μg ml^−1^. The embryos were laterally mounted on a glass-bottomed dish in 1% LMPA and imaged using a Leica SP8 confocal microscope with a ×40 oil immersion lens. The following primary antibodies were used: rabbit anti-LaminB1 (Abcam Ab16048) 1:100, mouse anti LaminB2 (Abcam AB8983) 1:100, mouse anti LaminA/C (Cell Signaling, 4777S) 1:100, rabbit anti-gammaH2AX (Genetex IHC-00059) 1:200, rat anti-RFP (Chromotek, 5F8) 1:200, chicken anti-GFP (Abcam Ab13970) 1:200, CPCA anti-mCherry (EnCor) 1:200, mouse F59 (DSHB) 1:50 and Col1a1a (GeneTex, GTX133063) 1:100. The following secondary antibodies were used at 1:500 dilution: Alexa 488 goat anti-chicken (A11039, Invitrogen), Alexa 546 goat antirat (A11081, Invitrogen), Alexa 546 goat anti-chicken (A11040, Invitrogen), Alexa 488 chicken anti-mouse (A21200, Invitrogen), Alexa 647 Fab goat anti-mouse (A48289, Invitrogen) and Alexa 647 Fab goat anti-rabbit (A48285, Invitrogen).

### Sample processing for electron microscopy and SEM imaging

Samples were fixed in fixative (2% glutaraldehyde/2% formaldehyde in 0.05 M sodium cacodylate buffer pH 7.4 containing 2 mM calcium chloride) overnight at 4 °C. The samples were then washed five times with 0.05 M sodium cacodylate buffer pH 7.4, and treated with 1% osmium tetroxide, 1.5% potassium ferricyanide, 0.05 M sodium cacodylate buffer pH 7.4 (osmicated) for three days at 4 °C. After washing five times in deionized (DI) water, the samples were treated with 0.1% (wt/vol) thiocarbohydrazide/DI water for 20 min at room temperature in the dark. After five washes in DI water, the samples were osmicated a second time for 1 h at room temperature (2% osmium tetroxide/DI water), washed again five times in DI water, and block-stained with uranyl acetate (2% uranyl acetate in 0.05 M maleate buffer pH 5.5) for three days at 4 °C. The samples were washed five times in DI water and then dehydrated in a graded series of ethanol (50%/70%/95%/100%/100% dry), and 100% dry acetonitrile, three times in each for at least 5 min. The samples were infiltrated with a 50/50 mixture of 100% dry acetonitrile/Quetol resin (without benzyldimethylamine (BDMA)) overnight, followed by three days in 100% Quetol (without BDMA). The samples were then infiltrated for five days in 100% Quetol resin with BDMA, exchanging the resin each day. The Quetol resin mixture comprised 12 g of Quetol 651, 15.7 g of nonenyl succinic anhydride, 5.7 g of methyl nadic anhydride and 0.5 g of BDMA (all from TAAB). Samples were placed in embedding moulds and cured at 60 °C for two days.

Thin sections (~200 nm) were cut using an ultramicrotome (Leica Ultracut E) and placed on melinex coverslips and allowed to air-dry. The coverslips were mounted on aluminium SEM stubs using conductive carbon tabs, and the edges of the coverslips were painted with conductive silver paint. The samples were then sputter-coated with 30-nm carbon using a Quorum Q150 T E carbon coater.

Samples were imaged in a Verios 460 scanning electron microscope (FEI/Thermofisher) at 4-keV accelerating voltage and 0.2-nA probe current in backscatter mode using the concentric backscatter detector (CBS) in immersion mode at a working distance of 3.5–4 mm. Stitched maps were acquired using FEI MAPS software using the default stitching profile and 5% image overlap.

### Acute tissue sectioning

Sox10:mG zebrafish embryos at 24 hpf were anaesthetized in MS222 and embedded in 2% low-gelling-temperature agarose (A9414, Sigma) in PBS. Transverse tissue sections of 500-µm thickness were prepared on a Leica VT1000S vibratome using Gillette Platinum blades in 0.3x Danieau on ice (forward speed 62.5 µm s^−1^, vibration frequency 60 Hz, vibration amplitude 600 µm). The agarose surrounding the tissue section was fixed to the bottom of a 40-mm-diameter tissue culture dish (93040, TPP) with cyanoacrylate, and the entire sample was submerged in phenol red-free L-15 medium with 2% fetal bovine serum (FBS) and 0.02% MS222.

### Atomic force microscopy

AFM measurements were conducted with a CellHesion 200 AFM head (JPK) with the sample located on a motorized precision stage (JPK) in a PetriDishHeater (JPK) set to 28 °C. AFM measurements were conducted with tipless silicon cantilevers (Arrow TL1, NanoWorld) with a spring constant of 0.047 N m^−1^ (determined in air using the in-built thermal noise method^109^). A spherical polystyrene bead with a diameter of 19.67 µm (PS/Q-R-B1246, microParticles) was glued (M-Bond 610-1 Adhesive, Micro-Measurements) to each cantilever. AFM measurements were conducted in force spectroscopy mode (force setpoint 5 nN, extend speed 20 µm s^−1^, sample rate 4,000 Hz, closed loop) in rectangular grids with a spacing of 6.4–8.5 µm. Force–distance curves were analysed with ForceDancer, custom-written MATLAB software (available upon request), using default settings. In brief, the baseline of each force–distance curve was determined and fitted with a linear model. The parameters of the linear model were then used to apply an offset and slope correction to the entire curve. Subsequently, the indentation part of the curve was determined and fitted with the Hertz model^110^ ($F=\frac{4}{3}K{R}^{1/2}{\delta }^{3/2}$, where *F* is the force, *R* is the indenter tip radius and *δ* is the indentation depth) to retrieve the apparent reduced elastic modulus *K*. We report the apparent reduced elastic modulus *K* instead of Young’s modulus *E* ($K=\frac{E}{1-{\nu }^{2}}$) to avoid assumptions about the value of Poisson’s ratio *v*. The indentation part of the curve used for fitting was refined in such a way to yield a *K* value representing the tissue properties at the setpoint force of 5 nN. Per animal, between 67 and 136 AFM measurements were analysed.

### In vitro assay

#### PDMS micropillar preparation

Micropillars were generated as described in ref. ^62^. Briefly, PDMS (Sylgard 184, Biesterfield) was mixed in a 1:10 elastomer-to-curing agent ratio, poured over a silicon wafer with negative micropillar structures (height of 11.3 μm and distances between the pillars of either 3 or 4 μm) imprinted by photolithography, degassed under vacuum, and cured at 80 °C overnight. The PDMS was cut into blocks of ~7 × 12 mm^2^, and 2-mm-sized loading holes were punched. The devices and glass coverslips, the latter cleaned with isopropanol and ethanol in an ultrasonic bath, were incubated in a plasma cleaner for 2 min, then combined, followed by bonding at 120 °C.

#### Neural crest culture preparation

Heterogeneous neural crest cultures were established as described in ref. ^61^, with some modifications. Briefly, embryos were anaesthetized in 0.02% tricaine (MS222) and dechorionated using 1 mg ml^−^^1^ pronase. For cranial neural crest cultures, 150 embryos at 14 hpf were collected and mechanically dissociated by trituration in calcium- and magnesium-free Dulbecco’s phosphate-buffered saline (DPBS; Corning, 21-040-CV) for 5 min. For trunk neural crest cultures, 150 embryos were manually dissected using forceps to remove the cranial regions, and trunk tissues were dissociated in DPBS using the same procedure. Cell suspensions were passed through a 40-μm cell strainer and centrifuged at 400*g* for 6 min. The resulting pellets were resuspended in complex medium, which consisted of DMEM/F12 without phenol red (Gibco, 21041-025), supplemented with 5% zebrafish embryo extract, 12% FBS, 0.02 mg ml^−^^1^ insulin, 20 ng ml^−^^1^ basic fibroblast growth factor (bFGF; Bio-Techne, 233-FB), 20 ng ml^−^^1^ epidermal growth factor (EGF; Bio-Techne, 236-EG), 1× N2 supplement (Gibco, 17502-048), 1 μg ml^−^^1^ amphotericin B (Sigma, A2942) and 50 U ml^−^^1^ penicillin–streptomycin (Gibco, 15070-063). The medium was sterile-filtered (0.22 μm) and used within one week of preparation.

#### Pillar forest device preparation and cell seeding

Pillar forest devices were fabricated as described above. Following fabrication, 2-mm loading holes were created using a biopsy punch, and devices were plasma-bonded to glass coverslips. The coverslip–device assemblies were then affixed to 35-mm glass-bottomed dishes from which the central glass had been removed, using UV-curable resin. Devices were activated by plasma-cleaning for 2 min at maximum power. PBS was added to all loading holes and allowed to fill the pillar chamber by capillary action, after which the devices were rinsed twice with PBS. The surfaces were coated with 100 μg ml^−^^1^ fibronectin diluted in PBS and incubated at 37 °C for 1 h, followed by two PBS washes. Devices were either used immediately or stored submerged in PBS at 4 °C overnight. On the day of the experiment, loading holes were filled with complete medium and the devices were equilibrated at 37 °C and 5% CO_2_ while cell suspensions were prepared. For seeding, medium was removed from the loading holes and 5 μl of cell suspension (10 × 10^4^ cells) was added per hole. Cells were allowed to adhere for 1 h at 37 °C and 5% CO_2_, after which dishes were filled with complete medium and returned to the incubator for imaging the following day.

### RNA sequencing

#### Photoconversion, dissociation and fluorescence-activated cell sorting

Sox10:H2B-Dendra2 embryos were selected, dechorionated and mounted laterally on a a glass-bottomed μ-Dish (35 mm, high) (ibidi, USA) in 5% carbomethylcellulose (Sigma) supplemented with 0.4 mg ml^−1^ of MS-22/tricaine. Using a Zeiss LSM 880 microscope, the mounted H2B-Dendra2 embryos were imaged using argon 488-nm and DPSS561-10 561-nm lasers, respectively, to determine and mark the location of the targeted NC cell within the trunk of each embryo. Only somites 10–15 were considered. For photoconversion, nuclei of premigratory (16 hpf) or migratory NC cells (23–24 hpf) were marked using circular ROIs, with premigratory NCs marked using a larger free-handed ROI encompassing multiple cells in the dorsal region of the embryo trunk, with a 405-nm UV diode 405-30 laser at 40% laser intensity and a pixel dwell of 131.07 μs for two iterations. For each population, eight embryos were photoconverted, aiming for four replicates. The software used to operate the microscope was Zen (v2.3 SP1). The trunks of 24-hpf embryos and their wild-type siblings (for cell sorting controls) were manually dissected immediately below the yolk sac with sharp forceps and transferred into 500 μl of chilled E3 solution (5 mM NaCl, 0.17 mM KCl, 0.33 mM CaCl_2_, 0.33 mM MgSO_4_) in a 1.5-ml Eppendorf tube. The dissected trunks were centrifuged at 400*g* for 1 min at 4 °C. The E3 medium was carefully removed from the samples, then 200 μl of 0.25% trypsin-EDTA in PBS was added to the samples and vigorously triturated using PBS-Triton-coated pipette tips, with each tip kept separately for each population, for up to 10 min per sample when no discernible clumps of tissue could be seen under a light microscope at room temperature. At this point, 10 μl of heat-inactivated fetal calf serum was added to the samples to stop enzymatic reactions. Afterwards, cell suspensions were centrifuged at 400*g* for 5 min at 4 °C, and the supernatant was removed and replaced with 200 μl of pre-cooled FACSmax cell dissociation solution (Amsbio). The samples were gently agitated for resuspension and centrifuged again at 400*g* for 5 min at 4 °C. The FACSmax supernatant was removed without agitating the cell pellet by pipetting, then replaced with 400 μl of fresh, cooled FACSmax solution and resuspended by gentle trituration. The solutions were then transferred into a Corning Falcon test tube with a Cell Strainer Snap Cap (Fisher Scientific) by vigorously pushing the cell suspension through the strainer with the pipette. This process was repeated for each sample. Samples were kept on ice for immediate FACS analysis. All cell dissociation steps were performed with RNase- and DNase-free pipette tips, Eppendorf tubes, and collection tubes within a sterile flow hood. The samples were sorted according to their fluorescence with a BD FACS ARIA III system under a flow hood at 4 °C, with the UV laser disabled to minimize the chances of photoconversion during the cell-sorting process. The first sample loaded was the wild-type zebrafish control sample, to determine the quality and behaviour of the cells, and allowing for adjustments of the gates separating cell debris, dead cells and doublets from live, viable, single cells. Next, 1 μl of 5 mg μl^−1^ DAPI cell marker was added to the samples to stain dead cells. The cells were gated for cell debris, single cells versus doublets or cell clumps, viability via the DAPI cell marker and finally for fluorescence. Cells expressing RFP and double-positive for RFP and GFP were sorted in to 9.3-μl cold, sterile PBS in 0.2-ml PCR tubes. Operation of the machinery involved in this protocol was performed with assistance of the Flow Cytometry Centre at Guy’s and St Thomas’ NHS Foundation Trust Biomedical Research Centre.

#### mRNA extraction and cDNA library production

The extraction of RNA, synthesis of complementary DNA (cDNA) libraries and initial quantifications were completed in collaboration with the KCL Genomics Centre. Twenty cells of migratory and/or premigratory cells were collected to generate their corresponding cDNA libraries. For each population, four biological replicates were collected. Samples were collected in 9.3 μl of sterile PBS, flash-frozen, and stored at −80 °C. RNA was extracted from the cells and made into cDNA libraries to be sequenced using the SMART-Seq v4 ultra low input kit (Takara Bio). Amplified cDNA was purified using an Agencourt AMPure XP kit (Beckman Coulter) and validated using the Agilent 2100 Bioanalyser and Agilent’s high sensitivity DNA kit (Agilent Technologies). Finally, the library was prepared for sequencing using the Nextera DNA library preparation kit (Illumina) with 100–150 pg of amplified cDNA.

#### RNA sequencing

The prepared cDNA libraries were quantified and analysed with Qubit Fluorometric Quantitation (Thermo-Fisher Scientific) for a sequencing run on the 2500 HiSeq platform (Illumina). The samples where then amplified using the Kappa library quantification kit (Roche), and 4 molar of the amplified samples were placed in all four lanes of the Illumina 2500 HiSeq sequencing system.

#### Bioinformatic analysis

Raw FASTQ sequence files were initially assessed for adapter contamination and over-represented sequences using FastQC. Adapter trimming and quality filtering were then performed using fastp^111^ with a custom adapter sequence list. Transcript abundance was quantified using Salmon^112^. A genome-aware Salmon index was generated using the concatenated zebrafish genome and transcriptome assembly (GRCz11) with a k-mer length of 23. Trimmed reads were pseudo-aligned to this index to obtain transcript-level quantifications. Salmon output was imported into R using the tximport package for downstream analysis. Differential gene expression between migratory and premigratory conditions was performed using DESeq2 (ref. ^113^). For principal component analysis, raw counts were transformed using variance-stabilizing transformation (VST), principal components were extracted using the plotPCA function, and plots were generated using ggplot2.

#### Plotting

RNA-seq downstream analyses and visualizations were performed in R. For principal component analysis, raw counts were transformed using VST, principal components were extracted using the plotPCA function, and plots were generated using ggplot2. Following DESeq2 analysis of migratory versus premigratory cell populations, differentially expressed genes (DEGs) were defined by an adjusted *P* value < 0.05 and absolute fold change > 2, and visualized via a volcano plot specifically highlighting DNA damage-related genes. A heatmap was then generated to display the expression patterns of 21 selected upregulated DNA damage-related DEGs. Gene Ontology (GO) enrichment analysis was performed for ‘Biological Processes’ using clusterProfiler (v4.16.0) and zebrafish annotation (org.Dr.eg.db). To minimize the redundancy of similar pathways, eight representative terms were displayed from the top 20 upregulated enriched terms, alongside eight representative terms displayed from the top ten downregulated enriched terms.

### Image analysis

All image analyses were performed by using ImageJ (Fiji (ImageJ 1.54 f) (NIH, https://imagej.net/fiji).

#### Extracellular space measurement

To quantify the extracellular space along the neural crest migratory path, dorsally acquired stacks were used. For cranial analysis, the space was measured in the region behind the eye. By using ‘Line tool’ a 5-pixel line was drawn across the extracellular space and an intensity plot profile was created with the ‘Plot profile’ function. The width of the distinguished high-intensity peak was measured at every fifth *z*-slize (2.5-µm interval) though the entire stack or until the signal was clear enough for analysis (Supplementary Fig. 1). For the trunk region, similarly, a line was drawn across all somites that could be followed from the dorsal-most region to the level of the neural tube. A line was drawn over the extracellular space normal to the somite at the mid aspect of each somite, and the distinguished high-intensity peak width was measured at every 2.5 µm from dorsal to ventral until the signal was clear enough for analysis.

#### Dynamic nuclear shape measurement

To obtain the parameters of neural crest nuclear shapes over time, maximum projected *z*-stacks of the nuclear channel with the best nuclear signal and minimum overlapping with neighbouring cells from each dataset were changed into 8-bit or 16-bit form and segmented using the 2D StarDist^114^ plugin in ImageJ. The Versatile (fluorescent nucleus) model was used with percentile between 25 and 100. From the segmented data, mostly nucleus transitioning from premigratory regions to migratory regions was manually notated and tracked over time. Nucleus tracking was continued until the cell divided or disappeared from the field of view. Daughter cells of the dividing cells were tracked as independent cells. Incorrectly segmented nuclei split into two or more units were corrected manually using the brush tool (5-pixel size) and merged together. Timepoints with incorrectly segmented merged nuclei were separated when possible or discarded from the analysis. For the premigratory to migratory transition analysis, with the original 4D *z*-stacks with dextran labelling the extracellular space, the timepoint when cells moved under the somite could be determined. Alternatively, the bright-field channel could be used to determine this timepoint in posterior videos. Stacks with selected and annotated nuclei were turned into the RGB format, and the following data-processing pipeline was applied: ‘Maximum filter’ with radius=1 (to help obtain the edges of the shape) > ‘Find edges’ function (to create an outline of the nucleus shape) > ‘Make binary’ function with method=MinError > ‘Skeletonize’ function (to refine the edges to help a separation of neighbouring nuclei) > ‘Analyze particles’ (to obtain shape parameters, including circularity and aspect ratio) (macro included in the data repository).

All the available timepoints along the tracks were included to the analysis. The overall track length varies due to cell division, migration out of the field of view, and occasional cell death. For minimum circularity alignment, cells with minimum circularity at the first or last timepoint of the track were excluded from the analysis.

#### Duration of deformation events

To analyse the duration of the deformation events, the mean circularity value of all migratory cells in all regions was first calculated (0.81); this was then set as a threshold, and circularity values below the threshold were defined as a deformation. Consecutive timepoints under the threshold were defined as one deformation event, and the duration of these events was calculated for each nucleus. Mean duration was calculated for those nuclei that had more than one deformation event.

#### Dynamic 53BP1 intensity analysis

To measure the 53BP1 intensity fluctuation of 53mApple or Sox10-emGFP-53BP1 together with nuclear shape over time, the ROIs of nuclear edges obtained from the 2D StarDist segmentation (see ‘Dynamic nuclear shape measurement’) were applied in the 53BP1 channel, and the mean and maximum intensities of the signal from the maximum projected stack were obtained together with the nuclear shape parameters by using the ‘Analyze particles’ function. To obtain the transient accumulation of the 53BP1 signal, the maximum intensity of 53BP1 was divided by the mean intensity of the nucleus at each timepoint. Similar to the shape analysis, all timepoints along the tracks were included to calculate the mean intensity per nucleus. For minimum circularity alignment, cells with minimum circularity at the first or last timepoint of the track were excluded from the analysis. For the 53BP1 intensity versus mean circularity correlation analysis, timepoints beyond 60 min after the minimum circularity event were excluded from the analysis.

To analyse bright foci, the mTrackJ (ref. ^115^) plugin was used to manually track foci over time.

#### Nucleocytoplasmic leakage of nls-emGFP

Image analysis was carried out using Fiji. Videos were corrected for Z and XY drift using the ‘Correct 3D Drift’ plugin in Fiji. Any *z*-stacks that contained migrating neural crest streams were maximum intensity projected. Nucleus deformation events were manually identified, and substacks of individual cells generated. Segmentation of the nucleus or cytoplasmic signal was carried out using the ‘Weka Segmentation’^116^ plugin. The nucleus mask was subtracted from the cytoplasmic binary image using the Fiji ‘Image Calculator’ function. Binary nucleus and cytoplasm segmentation masks were converted into ROIs using the ‘Analyse Particle’ function in Fiji, and ROIs were applied to the original videos to quantify nucleus or cytoplasm fluorescence intensity. The nucleus masks were used to measure nucleus shape descriptors using Fiji. For the cranial neural crest, cytoplasm segmentation was obtained by mosaically labelling NCs with sox10-lynTomato DNA^107^. Nucleus intensity values were normalized to their respective timepoint of higher nucleus circularity within 30 min of the maximum deformation event, defined as the timepoint of the minimum nucleus circularity, and cells were aligned to each other by identifying the timepoint of maximum deformation. For Halo-LaminB2 overexpression datasets and morpholino datasets, ROIs of nuclear edges obtained from the 2D StarDist segmentation (see ‘Dynamic nuclear shape measurement’) were applied in the nls-emGFP channel to obtain the mean intensity value at each timepoint. Nucleus intensity values were normalized to their respective timepoint of higher nucleus circularity within 18 min of the maximum deformation event.

#### Lap2b-EGFP intensity profiles

Image analysis was carried out using Fiji. Videos were corrected for Z and XY drift using the ‘Correct 3D Drift’ plugin in Fiji. Any *z*-stacks that contained migrating neural crest streams were maximum intensity projected. Nucleus deformation events were manually identified and substacks of individual cells generated. Segmentation of the nucleus signal was carried out using the ‘Weka Segmentation’ plugin. The nucleus masks were used to measure nucleus shape descriptors using Fiji. To quantify Lap2b-EGFP intensity profiles, the centre of the nucleus was identified manually in the 3D+t video stack. For every timepoint of a deformation event, a 10-pixel-wide line was traced from the posterior to the anterior of the nucleus along the direction of migration, and the ‘plot profile’ function in Fiji was used to measure fluorescence intensity along the selection. The fluorescence intensity along each pixel of the profile was normalized by the mean fluorescence intensity of each line selection. Because nuclei change in length over time, individual datapoints were binned into 20 bins for each profile, where 0% represented the back of the nucleus and 100% the front of the nucleus relative to the direction of cell migration. For each cell, the nucleus circularity was plotted to identify four phases of nucleus deformation (before deformation, during deformation, after deformation, recovery), and for the profiles of each phase were averaged together. For front/back polarization quantification, the rear peak of Lap2b-EGFP intensity was extracted by averaging the values between 5% and 30%, and the front peak was extracted by averaging the values between 75% and 90% for each intensity profile.

### γH2AX fluorescence intensity quantification

γH2AX immunostaining was carried out on sox10:mG zebrafish embryos. Briefly, *z*-stacks were reordered as a time series using the Hyperstack>Reorder Hyperstack function in Image J. DAPI was used to segment the nuclei of surrounding tissues, and sox10-H2B was used to segment the nuclei of NCs. Nuclear shapes were segmented using Stardist 2D. Segmented stacks were thresholded, and a 1-pixel watershed filter was applied to separate nuclear masks that were in contact with each other. Using the magic wand tool, individual nuclear masks corresponding to the mid *z* point of the NC nuclei were selected, added to the ROI manager, and annotated according to anatomical location (that is, premigratory or migratory tNC). Only one ROI was selected for each cell. For each NC nucleus, the nuclei of the immediate neighbours in the same *z*-stack were selected and added to the manager to generate a pool of *z*-matched surrounding neighbours (Supplementary Fig. 2). ROIs were then applied to the confocal *z*-stack, and the fluorescence intensities of γH2AX and nucleus shape descriptors were measured. The γH2AX mean fluorescence intensity for each NC nucleus of each image stack was normalized to the mean grey value of the *z*-matched surrounding tissue ROIs.

### LaminB2 fluorescence intensity quantification

LaminB2 immunostaining was carried out on sox10:Kalt4 zebrafish embryos, where H2B-mCherry was expressed under the sox10 promoter. Briefly, *z*-stacks were reordered as a time series using the Hyperstack>Reorder Hyperstack function in ImageJ, and sox10-H2B was used to segment the nuclei of NCs. Nuclear shapes were segmented using Stardist 2D. Segmented stacks were thresholded and eroded three times. Using the magic wand tool, individual nuclear masks corresponding to the mid *z* point of the NC nuclei were selected, added to the ROI manager, and annotated according to anatomical location (that is, premigratory or migratory tNCs). Only one ROI was selected for each cell. Each ROI was converted in a outline of 5 pixels to detect the NE using the ‘Make band’ function of ImageJ, added to the ROI manager, and annotated. NE ROIs were then applied to the confocal *z*-stack, and the fluorescence intensity of LaminB2 was measured. The LaminB2 mean fluorescence for each NE outline of each image stack was normalized to the mean grey value of the ROIs (Premigratory + Migratory tNC).

### Nuclear pore diameter measurement

Individual SEM ×10,000 images of trunk cross-sections (*n* = 25 cross-sections, from three embryos) were calibrated using the Image>Set Scale menu in ImageJ. Nuclei of premigratory and migratory tNCs were visually identified as described in Extended Data Fig. 4. Nuclear pores were visually identified as gaps in the double-layered NE, and nuclear pore cross-section width was manually measured using the Segmented Line tool in ImageJ.

### Statistical data analysis

The statistical tests used in each dataset, *P* values, number of data points (*n*) and number of independent embryos for each data panel are stated in the figure legends. In terms of sample size, for nuclear deformation studies, power analysis predicted that comparing nine cells per experimental condition might be sufficient to detect significant differences (*P* < 0.05, power 0.9) between samples. However, this is a small number of cells. We observed four or five migrating leader cells per neural crest stream and four or five streams per embryo, using a ×30 magnification. We thus analysed at least four videos from five embryos per condition whenever possible (5 × 4 = at least 20 cells per experimental condition). In terms of randomization, for laser-ablation studies, embryos injected with the correct set of fluorescent markers were randomly selected. For mutant studies, the *spt* mutation is recessive: when heterozygous parents were crossed, 25% of the progeny will carry the mutation, which could be morphologically assessed. An equal number of mutants or control siblings carrying the fluorescent reporter were randomly selected and mounted for live imaging. For fixed studies, embryos were randomly selected for fluorescence of the neural crest reporter and fixed. Data collection and analysis were not performed blind to the conditions of the experiments. Statistical tests were performed using GraphPad Prism version 10.4.1. (for Windows). The normality of the dataset was tested before further statistical analysis by using both Shapiro–Wilk test and Kolomogorov–Smirnov tests. All statistical tests were two-sided. To calculate the effect size, Cohen’s *d* was used with pooled standard deviation; *d* = (*M*_1_ − *M*_2_)/*S*_pooled_, where *M* is the mean per group. Data plots were generated using Python scripts (Python 3 reference manual^117^) with the Matplotlib and Seaborn libraries, and using GraphPad Prism. In line plots, mean and s.e.m. are presented. Box plots show the mean and first and third quartiles, and the whiskers extend from the box to the farthest data point lying within 1.5× the interquartile range (IQR) from the box.

### Reporting Summary

Further information on research design is available in the Nature Portfolio Reporting Summary linked to this Article.

## Online content

Any methods, additional references, Nature Portfolio reporting summaries, source data, extended data, supplementary information, acknowledgements, peer review information; details of author contributions and competing interests; and statements of data and code availability are available at https://doi.org/10.1038/s41556-026-02065-w.

## Supplementary information

Supplementary Information Supplementary Figs. 1 and 2.

Reporting Summary

Peer Review File

Supplementary Video 1 **Cranial neural crest migration**. Cranial neural crest migration in the region behind the eye at 10 hpf onwards, shown in maximum projection. Overlay of membranes (magenta) and nucleus (grey) labelled tissue specifically in the neural crest population using the Sox10mG fish line (left) and nucleus channel alone (right), highlighting the nuclear shape changes during cell migration. Time: hh:mm:ss. Anterior left, dorsal up.

Supplementary Video 2 **Anterior trunk neural crest migration**. Anterior tNC cells transitioning from the premigratory region to migratory region under the first two somites from 16 hpf onwards, shown in maximum projection. Overlay of membranes (magenta) and nucleus (grey) labelled tissue specifically in the neural crest population using the Sox10mG fish line (left) and nucleus channel alone (right), highlighting the nuclear shape changes during cell migration. Dextran labels the extracellular space (cyan). Time: hh:mm:ss. Anterior left, dorsal up.

Supplementary Video 3 **Mid trunk neural crest migration**. Mid tNC cells transitioning from the premigratory region to migratory region under two somites in the mid trunk region from 18 hpf onwards, shown in maximum projection. Overlay of membranes (magenta) and nucleus (grey) labelled tissue specifically in the neural crest population using the Sox10mG fish line (left) and nucleus channel alone (right), highlighting the nuclear shape changes during cell migration. Dextran labels the extracellular space (cyan). Time: hh:mm:ss. Anterior left, dorsal up.

Supplementary Video 4 **Posterior trunk neural crest migration**. Posterior tNC cells transitioning from the premigratory region to migratory region under the somite in the posterior trunk region from 23 hpf onwards, shown in maximum projection. Overlay of membranes (magenta) and nucleus (grey) labelled tissue specifically in the neural crest population using the Sox10mG fish line (left) and nucleus channel alone (right), highlighting the nuclear shape changes during cell migration. Dextran labels the extracellular space (cyan). Time: hh:mm:ss. Anterior left, dorsal up.

Supplementary Video 5 **Trunk neural crest cells migration in** ***spt*** **mutant embryos**. Mid tNC cells transitioning from the premigratory region to the migratory region in WT embryo (top) and in *spt* embryo (bottom), shown in maximum projection. Overlay of membranes (magenta) and nucleus (grey) labelled tissue specifically in the neural crest population using Sox10mG;*spt* fish (left) and nucleus channel alone (right), highlighting the nuclear shape changes during cell migration. Dextran labels the extracellular space (grey). Time: hh:mm:ss. Anterior left, dorsal up.

Supplementary Video 6 **Neural crest migration in laser ablated embryo**. Mid tNC cells transitioning from the premigratory region to the migratory region in an unablated adjacent region (left) and in a laser-ablated region (right), shown in maximum projection. Nuclei are labelled specifically in the neural crest population using the Sox10mG fish line, showing the nucleus shape before and after entering the migratory region. Time: hh:mm:ss. Anterior left, dorsal up.

Supplementary Video 7 **nls-emGFP dynamics in trunk migratory neural crest**. Mid tNC cells transitioning from the premigratory region to the migratory region, showing the neural crest-specific nlsGFP signal in the Sox10::Kalta4;UAS-nls-emGFP fish line, shown in maximum projection. The video shows nlsGFP localization in the cytoplasm and nucleus before, during and after a nuclear deformation event. Time: hh:mm:ss. Anterior left, dorsal up.

Supplementary Video 8 **nls-emGFP dynamics in trunk premigratory neural crest**. Mid tNC cells in the premigratory region showing the neural crest-specific nlsGFP signal in the Sox10::Kalta4;UAS-nls-emGFP fish line, shown in maximum projection. The video shows nlsGFP localization in the cytoplasm and nucleus. Time: hh:mm:ss. Anterior left, dorsal up.

Supplementary Video 9 **BAF-mCherry localization during trunk neural crest migration**. Injected BAF-mCherry mRNA localization in mid tNCs in the migratory region during and after a nuclear deformation event, shown in maximum projection. The overlay identifies the neural crest cells in green by showing the neural crest-specific nlsGFP signal in the Sox10::Kalta4;UAS-nls-emGFP fish line. The mid panel shows the location of the neural crest nucleus. The right panel shows homogenous BAF localization during and after the deformation event. Time: hh:mm:ss. Anterior left, dorsal up.

Supplementary Video 10 **cGAS-GFP in migratory trunk neural crest cell**. NC-specific cGAS-GFP expression (green) in migratory NC cells in the midtrunk region upon a high nuclear deformation event, shown in maximum projection. H2B-cherry shows the NC nucleus (magenta). Time: hh:mm. Anterior left, dorsal up.

Supplementary Video 11 **Lap2β-EGFP dynamics in migratory trunk neural crest cells**. Injected lap2β mRNA localization in tNCs in the migratory region before, during and after a deformation event, shown in maximum projection. The overlay identifies neural crest cells in green by showing the neural crest-specific nucleus signal in sox10Kalt4 embryos, where chromatin is labelled with H2B-mCherry. The white arrowhead shows the lap2β around the nucleus in non-deformed migratory tNC cells, and the white arrowheads show the depletion of lap2β during nuclear deformation events. Time: mm:ss. Anterior left, dorsal up.

Supplementary Video 12 **53BP1-dynamics during the trunk neural crest premigratory to migratory transition**. Injected mApple-53BP1 trunk mRNA localization in mid tNCs during the premigratory to migratory transition are shown in maximum projection. The overlay identifies the neural crest cells in green by showing the neural crest-specific nlsGFP signal in the Sox10::Kalta4;UAS-nls-emGFP fish line before cells enter the migratory region (left panel, orange arrowheads). White arrowheads (right panel) show the transient accumulation of 53BP1 foci. Time: hh:mm. Anterior left, dorsal up.

Supplementary Video 13 **mApple-53BP1-dynamics in premigratory trunk neural crest**. Injected mApple-53BP1 trunk mRNA localization in mid tNCs in the premigratory region, shown in maximum projection. The overlay identifies neural crest cells in green by showing the neural crest-specific nlsGFP signal in the Sox10::Kalta4;UAS-nls-emGFP fish line (left panel, orange arrowheads), shown in maximum projection. White arrowheads (right panel) show the transient accumulation of 53BP1 foci. Time: hh:mm. Anterior left, dorsal up.

Supplementary Video 14 **Midtrunk NC nucleus dynamics in laminB2 injected embryos**. Neural crest cell migration in the mid trunk region in control MO (left) and LaminB2 MO (right) injected embryos in the Sox10::Kalta4;UAS-nls-emGFP fish line, shown in maximum projection. Time: hh:mm. Anterior left, dorsal up.

Supplementary Video 15 **Halo-LaminB2 expression in trunk neural crest cells**. UAS-Halo-LaminB2 tissue-specific expression in migratory neural crest cells in the midtrunk region in the Sox10::Kalta4;UAS-nls-emGFP fish line, shown in maximum projection. Time: hh:mm. Anterior left, dorsal up.

Supplementary Video 16 **Em53BP1-GFP dynamics in Halo-LaminB2 positive and negative neural crest during migration**. 53BP1 and LaminB2 double-positive NC cells (white arrows) and LaminB2 negative cells (cyan arrows) in the midtrunk region in Sox10-emGFP-53P1×UAS-Halo-LaminB2 embryos, shown in maximum projection. Arrowheads indicates the foci appearance in LaminB2-positive cells (green) and in Halo-LaminB2-negative cells (yellow). Time: hh:mm. Anterior left, dorsal up.

## Source data

Source Data Figs. 1–8 Statistical source data, organized by panel.

Source Data Extended Data Figs. 1–8 Statistical source data, organized by panel.

## Data Availability

Data supporting the findings of this study are available from the corresponding author on reasonable request. Sequencing data that support the findings of this study have been deposited in the Gene Expression Omnibus (GEO) under accession code GSE330051. Source data are provided with this paper.

## Code availability

Code used in this study for data plotting, RNA-seq analysis and plotting is available on Zenodo at https://doi.org/10.5281/zenodo.21375498 (ref. ^118^). Code used in this study for analysis of AFM raw data is available from the authors (J.M.B. and K.F.) upon request.
