# Supplementary material for: In vivo DNA damage protection during cell migration across confining embryonic tissue environments
Source: Nat Cell Biol. 2026 Oct 9;28(10):2088–105. doi: 10.1038/s41556-026-02065-w (PMC13661264; doi:10.1038/s41556-026-02065-w)
Supplement: Supplementary file 1 [file 41556_2026_2065_MOESM1_ESM.pdf]

nature cell biology

Supplementary information https://doi.org/10.1038/s41556-026-02065-w

In vivo DNA damage protection during cell migration across confining embryonic tissue environments

In the format provided by the authors and unedited

Supplementary Figures

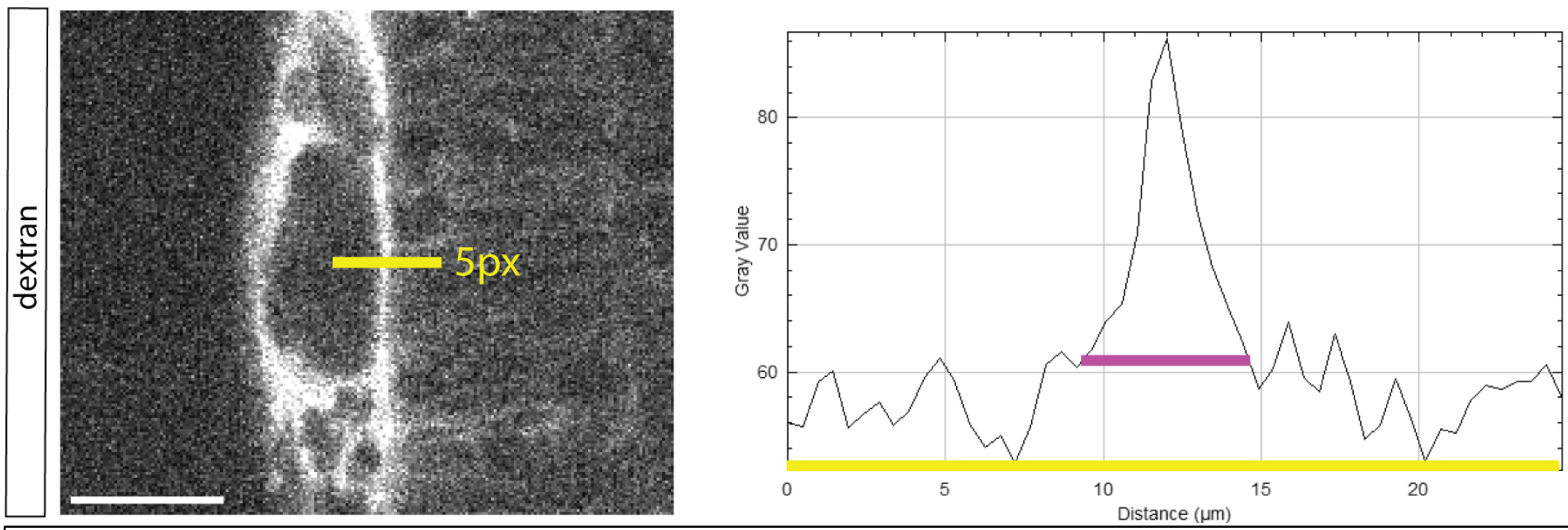

Supplementary Figure 1. Example of a line selection drawn across the somite-neural tube interstitial space on a transverse section of a dorsally oriented embryo, and example of a intensity profile. The base of the peak was used to measure the width of the space. Scale bar 20µm.

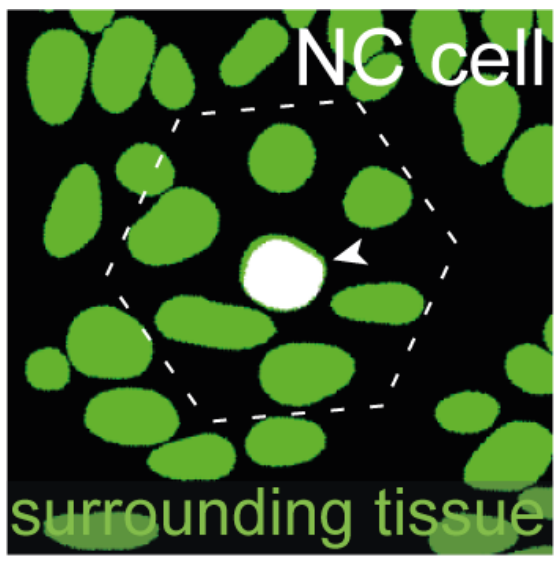

Supplementary Figure 2. Example of NC nucleus mask (arrowhead) and its surrounding tissue neighborhood (dashed hexagonal line).
