# Supplementary material for: In vivo DNA damage protection during cell migration across confining embryonic tissue environments
Source: Nat Cell Biol. 2026 Oct 9;28(10):2088–105. doi: 10.1038/s41556-026-02065-w (PMC13661264; doi:10.1038/s41556-026-02065-w)
Supplement: Supplementary file 2 [file 41556_2026_2065_MOESM2_ESM.pdf]

nature portfolio

Corresponding author(s): Elena Scarpa

Last updated by author(s): Jul 9, 2026

Reporting Summary

Nature Portfolio wishes to improve the reproducibility of the work that we publish. This form provides structure for consistency and transparency in reporting. For further information on Nature Portfolio policies, see our Editorial Policies and the Editorial Policy Checklist.

Statistics

For all statistical analyses, confirm that the following items are present in the figure legend, table legend, main text, or Methods section.

n/a	Confirmed	
☐	☒	The exact sample size (*n*) for each experimental group/condition, given as a discrete number and unit of measurement
☐	☒	A statement on whether measurements were taken from distinct samples or whether the same sample was measured repeatedly
☐	☒	The statistical test(s) used AND whether they are one- or two-sided<br>*Only common tests should be described solely by name; describe more complex techniques in the Methods section.*
☒	☐	A description of all covariates tested
☐	☒	A description of any assumptions or corrections, such as tests of normality and adjustment for multiple comparisons
☐	☒	A full description of the statistical parameters including central tendency (e.g. means) or other basic estimates (e.g. regression coefficient) AND variation (e.g. standard deviation) or associated estimates of uncertainty (e.g. confidence intervals)
☐	☒	For null hypothesis testing, the test statistic (e.g. *F*, *t*, *r*) with confidence intervals, effect sizes, degrees of freedom and *P* value noted<br>*Give P values as exact values whenever suitable.*
☒	☐	For Bayesian analysis, information on the choice of priors and Markov chain Monte Carlo settings
☒	☐	For hierarchical and complex designs, identification of the appropriate level for tests and full reporting of outcomes
☐	☒	Estimates of effect sizes (e.g. Cohen's *d*, Pearson's *r*), indicating how they were calculated

Our web collection on statistics for biologists contains articles on many of the points above.

Software and code

Policy information about availability of computer code

Data collection	Spinning disc live imaging datasets were acquired using Volocity(v6.3, v7.0) or NIS Elements. Confocal imaging datasets were acquired using LasX v3.5 (Leica) or Fluoview software (Olympus/Evident).
Data analysis	Data were analysed using Fiji v1.54f(NIH, https://imagej.net/fiji). Nucleus segmentation was carried out using the Stardist 2D plugin embedded in Fiji (https://github.com/stardist/stardist). Statistical tests were performed using GraphPad Prism version 10.4.1. (for Windows). Data plots were generated using Python 3, R and GraphPad Prism. Code used in this study for data plotting, RNA-seq analysis and plotting is available on Zenodo: https://doi.org/10.5281/zenodo.21375498 . Code used in this study for analysis of AFM raw data is available from the authors (JMB, KF) upon request.

For manuscripts utilizing custom algorithms or software that are central to the research but not yet described in published literature, software must be made available to editors and reviewers. We strongly encourage code deposition in a community repository (e.g. GitHub). See the Nature Portfolio guidelines for submitting code & software for further information.

Data

Policy information about availability of data

All manuscripts must include a data availability statement. This statement should provide the following information, where applicable:

- Accession codes, unique identifiers, or web links for publicly available datasets
- A description of any restrictions on data availability
- For clinical datasets or third party data, please ensure that the statement adheres to our policy

Statistical source data are provided with this study. All other data supporting the findings of this study are available from the corresponding author on reasonable request. Sequencing data that support the findings of this study have been deposited in the Gene Expression Omnibus (GEO) under accession code GSE330051.

Research involving human participants, their data, or biological material

Policy information about studies with human participants or human data. See also policy information about sex, gender (identity/presentation), and sexual orientation and race, ethnicity and racism.

Reporting on sex and gender	n/a
Reporting on race, ethnicity, or other socially relevant groupings	n/a
Population characteristics	n/a
Recruitment	n/a
Ethics oversight	n/a

Note that full information on the approval of the study protocol must also be provided in the manuscript.

Field-specific reporting

Please select the one below that is the best fit for your research. If you are not sure, read the appropriate sections before making your selection.

☒ Life sciences ☐ Behavioural & social sciences ☐ Ecological, evolutionary & environmental sciences

For a reference copy of the document with all sections, see nature.com/documents/nr-reporting-summary-flat.pdf

Life sciences study design

All studies must disclose on these points even when the disclosure is negative.

Sample size	For nuclear deformation studies, power analysis predicted that comparing 9 cells per experimental condition might be sufficient to detect significant differences ($p<0.05$, power 0.9) between samples. However, this is a small number of cells. We observe 4/5 migrating leader cells per neural crest stream, 4/5 streams per embryo using a 30X magnification. Therefore we analysed at least 4 movies from 5 embryos per condition whenever possible (5x4=at least 20 cells per experimental condition).
Data exclusions	no datapoints were excluded from statistical analysis. Quality control for xyz drift of live 4D datasets and embryo survival was carried out (ie. movies were excluded from analysis if the embryo died during live imaging or if the cells of interest disappeared from the field of view due to drift or xy stage instability).
Replication	All attempts at replication were successful.<br><br>Embryos were bred from breeding tanks where 3-4 females were crossed to 2-3 males. As Zebrafish fertilisation occurs in water, each embryo in such clutches might have a different set of parents and hence each embryo is considered to be an independent experiment.<br>To take into account biological variation between individual embryos during live imaging, we attempted to analyse at least 4 movies from 2-5 embryos per condition from at least 2-3 independent crosses whenever possible.<br>For mosaic mRNA analysis, as only a subset of neural crest cells may receive the mRNA it was more difficult to achieve high numbers of injected cells despite an increased number of biological replicates. However, as our power analysis for nuclear deformation studies had predicted that comparing 9 cells per experimental condition might be sufficient to detect significant differences ($p<0.05$) between groups, we occasionally accepted a lower number of cells for analysis of mosaic injections datasets.<br>For fixed analysis of endogenous antibody stainings at least 2-3 independent experiments (3 independent crosses) with a minimum of 5 embryos per dataset were analysed.
Randomization	For laser ablation studies, embryos injected with the correct set of fluorescent markers were randomly selected. For mutant studies, the spt mutation is recessive: when heterozygous parents were crossed, 25% of the progeny will carry the mutation, which could be morphologically

assessed. An equal number of mutants or control siblings carrying the fluorescent reporter were mounted for live imaging. For fixed studies, embryos were randomly selected for fluorescence of the neural crest reporter and fixed.

Blinding

Blinding was not used in this study. In experimentally perturbed embryos (ie. spt mutant, laser ablated embryos) the gross morphological changes in embryo anatomy caused by the perturbation were such that blinding would not be possible. Similarly, when different AP positions were compared, variations in embryo anatomy along the anteroposterior axis made blinding not possible.

Reporting for specific materials, systems and methods

We require information from authors about some types of materials, experimental systems and methods used in many studies. Here, indicate whether each material, system or method listed is relevant to your study. If you are not sure if a list item applies to your research, read the appropriate section before selecting a response.

Materials & experimental systems

n/a	Involved in the study
☐	☒ Antibodies
☒	☐ Eukaryotic cell lines
☒	☐ Palaeontology and archaeology
☐	☒ Animals and other organisms
☒	☐ Clinical data
☒	☐ Dual use research of concern
☒	☐ Plants

Methods

n/a	Involved in the study
☒	☐ ChIP-seq
☒	☐ Flow cytometry
☒	☐ MRI-based neuroimaging

Antibodies

Antibodies used

Primary antibodies used to detect endogenous Zebrafish proteins:
Rabbit anti-LaminB1 (Abcam Ab16048) , Mouse anti LaminB2 (Abcam AB8983), Mouse anti LaminA/C (Cell Signaling, 4777S), Rabbit anti-gammaH2AX (Genetex IHC-00059), Mouse F59 (DSHB), Col1a1a (GeneTex, GTX133063).
Primary antibodies to detect fluorescently tagged proteins: Rat anti-RFP (Chromotek, 5F8), Chicken anti-GFP (Abcam Ab13970, , CPCA anti-mCherry (EnCor) .
Secondary antibodies: Alexa 488 goat anti-chicken (A11039,Invitrogen); Alexa 546 Goat AntiRat (A11081, Invitrogen); Alexa 546 Goat Anti-chicken (A11040, Invitrogen); Alexa 488 chicken anti-mouse (A21200,Invitrogen); Alexa 647 Fab goat anti-mouse (A48289,Invitrogen); Alexa 647 Fab goat anti-rabbit (A48285,Invitrogen).

Validation

Rabbit anti-LaminB1 (Abcam Ab16048) :Yanakieva et al, 2019: https://doi.org/10.1083/jcb.201901077
Mouse anti LaminB2 (Abcam AB8983): Madakashira et al, 2021 DOI: 10.3390/genes12071081
Mouse anti LaminA/C (Cell Signaling, 4777S) :https://www.cellsignal.com/products/primary-antibodies/lamin-a-c-4c11-mouse-mab/4777?srsltid=AfmBOopMdI-IaDNa8G8WdgxKgGwHWsJDKZwVph67eekLARp6BZrCgN_m . 596 citations
Validated in Zebrafish in this study (Supplementary Figure 6). I also have a personal communication from Ms Laura Bader (Rashmi Priya lab, Crick institute) who also validated the same antibody in Zebrafish.
Rabbit anti-gammaH2AX (Genetex IHC-00059): Vierstraete et al 2021 DOI: 10.1016/j.dnarep.2020.103023
Mouse F59 (DSHB): Honjo and Eisen, 2005 10.1242/dev.02026
Col1a1a (GeneTex, GTX133063): Lencer et al, 2023 DOI: 10.1242/dev.201304

Primary antibodies to detect fluorescently tagged proteins: Rat anti-RFP (Chromotek, 5F8: https://www.ptglab.com/products/RFP-antibody-5F8.htm?srsltid=AfmBOooVF5DIWEcG-kMZgC7I1jwUDwma45gqqVW48dhXV3jyLu_FJCXE, 708 publications) 1:200, Chicken anti-GFP (Abcam Ab13970, https://www.abcam.com/en-us/products/primary-antibodies/gfp-antibody-ab13970?srsltid=AfmBOoppZFiwf0-1uqDOPHj1WiyqU0ivTNgEYvAjTriWfy6FTOdDAVmj, 3612 publications) 1:200, CPCA anti-mCherry (EnCor) 1:200 (https://encorbio.com/products/chicken-polyclonal-antibody-to-mcherry-cat-cpca-mcherry).

Alexa 488 goat anti-chicken (A11039,Invitrogen:https://www.thermofisher.com/antibody/product/Goat-anti-Chicken-IgY-H-L-Secondary-Antibody-Polyclonal/A-11039, 2866 citations); Alexa 546 Goat AntiRat (A11081, Invitrogen: https://www.thermofisher.com/antibody/product/Goat-anti-Rat-IgG-H-L-Cross-Adsorbed-Secondary-Antibody-Polyclonal/A-11081, 504 citations); Alexa 546 Goat Anti-chicken (A11040, Invitrogen: https://www.thermofisher.com/antibody/product/Goat-anti-Chicken-IgY-H-L-Secondary-Antibody-Polyclonal/A-11040, 122 citations); Alexa 488 chicken anti-mouse (A21200,Invitrogen:https://www.thermofisher.com/antibody/product/Chicken-anti-Mouse-IgG-H-L-Cross-Adsorbed-Secondary-Antibody-Polyclonal/A-21200, 325 citations); Alexa 647 Fab goat anti-mouse (A48289,Invitrogen, https://www.thermofisher.com/antibody/product/Goat-anti-Mouse-IgG-H-L-Cross-Adsorbed-Secondary-Antibody-Polyclonal/A48289, 7 citations, validated in Zebrafish by Yanakieva et al, JCB, https://doi.org/10.1083/jcb.201901077); Alexa 647 Fab goat anti-rabbit (A48285,Invitrogen,https://www.thermofisher.com/antibody/product/Goat-anti-Rabbit-IgG-H-L-Cross-Adsorbed-Secondary-Antibody-Polyclonal/A48285, 7 citations, validated in Zebrafish by Yanakieva et al, JCB, https://doi.org/10.1083/jcb.201901077).

Animals and other research organisms

Policy information about studies involving animals; ARRIVE guidelines recommended for reporting animal research, and Sex and Gender in Research

Laboratory animals	Zebrafish were maintained and raised at 28°C on a 10h/14h dark/light cycle according to standard conditions. The following zebrafish lines were used in this study; Wild type (AB and TLF), Sox10:mG (Richardson et al., 2016) Sox10-Kalt4 (Alhashem et al., 2021) Sox10::KaltA4 (Almeida et al, 2015) ;UAS-nls-emGFP (this study) , spt-1 (b104r1) (Kimmel et al, 1989), sox10:emGFP-53BP1 (this study), UAS-cGASa-EGFP (this study), UAS-Halo-LaminB2 (this study).
Wild animals	n/a
Reporting on sex	Zebrafish embryos under 24 hours of age were used in this study. In Zebrafish, dimorphic sex differences in the gonads only become evident at 4-5 days post fertilisation (Aharon, D. & Marlow, F. L. Sexual determination in zebrafish. Cell. Mol. Life Sci. 79, 8 (2022)). Therefore, it was not possible to distinguish the sex of the embryos at the developmental stage analysed in this study.
Field-collected samples	n/a
Ethics oversight	Work was carried out under the Animal (Scientific Procedures) Act 1986 legislation under the HO Project Licence number PP8606293 (PPL holder: Dr. Elena Scarpa). The licence was submitted tho the Home Office upon ethical approval of the University of Cambridge AWERB committee in compliance with the ARRIVE and PREPARE guidelines.

Note that full information on the approval of the study protocol must also be provided in the manuscript.

Plants

Seed stocks	n/a
Novel plant genotypes	n/a
Authentication	n/a
