# Supplementary material for: In vivo DNA damage protection during cell migration across confining embryonic tissue environments
Source: Nat Cell Biol. 2026 Oct 9;28(10):2088–105. doi: 10.1038/s41556-026-02065-w (PMC13661264; doi:10.1038/s41556-026-02065-w)
Supplement: Supplementary file 3 [file 41556_2026_2065_MOESM3_ESM.pdf]

nature portfolio

Peer Review File

In vivo DNA damage protection during cell migration across confining embryonic tissue environments

Corresponding Author: Dr Elena Scarpa

Version 0:

Decision Letter:

*Please delete the link to your author homepage if you wish to forward this email to co-authors.

Dear Dr Scarpa,

I apologize for the delay. Your manuscript, "In vivo nuclear envelope adaptation during cell migration across confining embryonic tissue environments", has now been seen by 3 referees, who are experts in cell-ECM interactions in 3D (referee 1); nuclear mechanics (referee 2); and neural crest and migration (referee 3). As you will see from their comments (attached below) they find this work of potential interest, but have raised substantial concerns, which in our view would need to be addressed with considerable revisions before we can consider publication in Nature Cell Biology.

Nature Cell Biology editors discuss the referee reports in detail within the editorial team, including the chief editor, to identify key referee points that should be addressed with priority, and requests that are overruled as being beyond the scope of the current study. To guide the scope of the revisions, I have listed these points below. We are committed to providing a fair and constructive peer-review process, so please feel free to contact me if you would like to discuss any of the referee comments further.

I should stress that the referees' concerns point to unclear mechanistic links which would need to be addressed with experiments and data, and reconsideration of the study for this journal and re-engagement of referees would depend on strength of these revisions.

In particular, it would be essential to:

A) Experimentally address concerns underlying mechanisms for potential resistant of NC cells to nuclear / DNA damage with additional functional data (all Reviewers)

B) Provide further experimental evidence to buttress claims about underlying molecular mechanisms (all Reviewers)

C) All other referee concerns pertaining to strengthening existing data, providing controls, methodological details, clarifications and textual changes, should also be addressed.

D) Finally please pay close attention to our guidelines on statistical and methodological reporting (listed below) as failure to do so may delay the reconsideration of the revised manuscript. In particular please provide:

- a Supplementary Figure including unprocessed images of all gels/blots in the form of a multi-page pdf file. Please ensure that blots/gels are labeled and the sections presented in the figures are clearly indicated.

- a Supplementary Table including all numerical source data in Excel format, with data for different figures provided as different sheets within a single Excel file. The file should include source data giving rise to graphical representations and statistical descriptions in the paper and for all instances where the figures present representative experiments of multiple independent repeats, the source data of all repeats should be provided.

We would be happy to consider a revised manuscript that would satisfactorily address these points, unless a similar paper is published elsewhere, or is accepted for publication in Nature Cell Biology in the meantime.

When revising the manuscript please:

- ensure that it conforms to our format instructions and publication policies (see below and https://www.nature.com/nature/for-

authors).

- provide a point-by-point rebuttal to the full referee reports verbatim, as provided at the end of this letter.

- for any revision that includes light microscopy data, we ask our authors to please include a completed light microscopy reporting table https://www.nature.com/documents/Light_microscopy_reporting_table.xlsx to ensure the methods are described thoroughly. The table will be available to reviewers and ultimately published should the manuscript be accepted at the journal.

- provide the completed Reporting Summary (found here https://www.nature.com/documents/nr-reporting-summary.pdf). This is essential for reconsideration of the manuscript will be available to editors and referees in the event of peer review. For more information see http://www.nature.com/authors/policies/availability.html or contact me.

When submitting the revised version of your manuscript, please pay close attention to our href="https://www.nature.com/nature-portfolio/editorial-policies/image-integrity">Digital Image Integrity Guidelines. and to the following points below:

-- that unprocessed scans are clearly labelled and match the gels and western blots presented in figures.
-- that control panels for gels and western blots are appropriately described as loading on sample processing controls
-- all images in the paper are checked for duplication of panels and for splicing of gel lanes.

Finally, please ensure that you retain unprocessed data and metadata files after publication, ideally archiving data in perpetuity, as these may be requested during the peer review and production process or after publication if any issues arise.

Nature Cell Biology is committed to improving transparency in authorship. As part of our efforts in this direction, we are now requesting that all authors identified as ‘corresponding author’ on published papers create and link their Open Researcher and Contributor Identifier (ORCID) with their account on the Manuscript Tracking System (MTS), prior to acceptance. ORCID helps the scientific community achieve unambiguous attribution of all scholarly contributions. You can create and link your ORCID from the home page of the MTS by clicking on ‘Modify my Springer Nature account’. For more information please visit please visit www.springernature.com/orcid.

This journal strongly supports public availability of data. Please place the data used in your paper into a public data repository, or alternatively, present the data as Supplementary Information. If data can only be shared on request, please explain why in your Data Availability Statement, and also in the correspondence with your editor. Please note that for some data types, deposition in a public repository is mandatory - more information on our data deposition policies and available repositories appears below.

Please submit the revised manuscript files and the point-by-point rebuttal to the referee comments using this link:

Link Redacted

*This url links to your confidential home page and associated information about manuscripts you may have submitted or be reviewing for us. If you wish to forward this email to co-authors, please delete the link to your homepage.

We would like to receive a revised submission within six months.

We hope that you will find our referees' comments, and editorial guidance helpful. Please do not hesitate to contact me if there is anything you would like to discuss.

Best wishes,

Daryl

Daryl Jason Verzosa David, PhD

Senior Editor, Nature Cell Biology
Advisory Editor, npj Biological Physics and Mechanics
Nature Portfolio

Heidelberger Platz 3, 14197 Berlin, Germany
Email: daryl.david@nature.com
ORCID: https://orcid.org/0000-0002-9253-4805

Reviewers' Comments:

Reviewer #1 (Remarks to the Author):

This manuscript provides novel, potentially interesting evidence that in vivo deformation of neural crest stem cell nuclei does not cause DNA damage even as the cells and nuclei undergo high deformation in very narrow tissue spaces. These findings are valuable in apparently contradicting previous purely in vitro studies, though the authors point out that these differences might be related to the stem cell nature of neural crest cells. They report that by careful measurements of the width of extracellular spaces from cranial to caudal the “level of tissue scale confinement increases along the antero-posterior axis” reflected in altered nuclear morphology; the latter is quantified to show significant nuclear deformation by measuring both nuclear circularity and aspect ratio. The authors use a genetic model in which tissue spaces are enlarged as well as simple laser ablation of adjacent tissue to experimentally prevent the nuclear deformation. They report that their neural crest cells are able to reduce DNA damage under confinement even with the loss of soluble intranuclear molecules resulting from nuclear pore enlargement. This protection is reportedly associated with localised reduction in nuclear lamin B2 levels when the cells are within confined spaces but not before and after this confinement. The authors posit the interesting concept that the neural crest cells show “dynamic adaptation to tissue confinement in vivo” without the DNA damage that most in the field would have expected with this extent of nuclear deformation.

This reviewer is enthusiastic about the approach used in this clearly written and presented study to test the role of confinement of migrating cells in vivo with the very interesting and potentially important finding that neural crest cells do not display the expected level of DNA damage after even major nuclear deformation. On the other hand, there are some substantive reservations. For example, the evidence for the statement that “level of tissue scale confinement increases along the antero-posterior axis” is weakened slightly by the quantitative posterior embryo data, and the evidence for “dynamic adaptation to tissue confinement in vivo” providing protection is weak. Overall, the authors will need to use revised text consistent with their data and to avoid the temptation to over-interpret in overly broad conclusions.

Major points:

1. I sympathize with the authors that there is an unfortunate discrepancy between the morphological data showing significantly lower nuclear circularity plus deformation with increasingly posterior localization (Figures 1C and Supplementary Fig. 2D) compared to the non-significant quantification of mean spaces in mid versus posterior tissues in Figure 2F. Yet the examples shown in Figures 2E and F show that there is closer spacing between tissues in posterior trunk tissues, whereas the quantitative data actually show no differences between mid and posterior trunk tissues, and if anything a very slight non-significant widening in posterior tissues. The authors need to choose images that match the quantitative data, regardless of the possible dilution of the simplicity of the story. Instead, there may well be other causes/reasons besides merely tissue space differences, such as differing elasticity or local matrix/tissue pore size in posterior regions. In fact, if there is more collagen posteriorly, that might explain the morphological data. If the authors wish to claim an increase in tissue scale confinement by pointing to the nuclear morphological data, they need to make this point clear, since measurements of tissue spaces do not show a further decrease in the posterior region of embryos.

2. The reported alterations in nuclear lamin localisation in Figure 6 during deformation initially seemed quite intriguing in support of the concept of dynamic adaptation to tissue confinement. However, there are problems with the data here and in the supplementary data for this figure. First, even though the differences are highly statistically significant during deformation, the actual difference seems to be quite small, i.e., less than 5% because the Y axis scale is so expanded, with an entire 0.8 of the scale missing. Second, the authors do not show a statistical comparison of the nucleus front versus rear in the ‘Before deformation’ state. Finally, in Supplementary Figure 6C, there is an equally major, important and statistically notable decrease in localisation at the nucleus front during deformation of cranial when compared to more-posterior neural crest cells, yet the authors state in the text that the localisation “does not show a polarized localisation” - whereas the data show that there is an equally significant reverse localisation! These issues will need to be resolved by significantly toning down the claim and clarifying the findings in the text.

3. Figure 1 shows striking heterogeneity in the outlines of cells at the different levels of neural crest from cranial to posterior consistent with differences in confinement. The morphologies of cells can provide a good indication of the lateral forces on cells and nuclei, so a key question is whether the examples shown are representative of the shapes of cells at each of these different levels. For example, are the cranial neural crest cells really that strikingly varying in their protrusions compared to those at all the other levels, and are the mid-trunk neural crest cells so laterally compressed compared to those in the anterior and posterior neural crest regions? It would be much better for the authors to provide truly representative outlines of cell bodies both here and perhaps in expanded supplemental materials showing readers the range of cell morphologies at each of the different levels.

4. It is surprising to this reviewer that the quantified mean nuclear circularity of the neural crest cells shows disturbingly quite modest differences in effect size, albeit highly statistically significant. It would be useful, perhaps in supplementary material, for the authors to indicate the proportions of cells that show striking differences in morphology versus those that are unaffected compared to evidence of DNA damage. The reason for this concern is to ask the important question: do cells with the largest differences in nuclear deformation still show no significant evidence of DNA damage?

5. The findings presented in the study are important in providing an in vivo test of the role of confinement/tissue spaces in

nuclear morphology and DNA damage. They are obviously more authentic biologically than the multiple in vitro studies published in recent literature. However, there is one potential major difference: tissues in vivo have variable boundaries and most importantly local elasticity and viscoelasticity. It is conceivable that the in vitro studies were misleading due to the nature of the confining material. To substantially enhance the importance of the findings in this study and to evaluate more rigorously the conclusion that neural crest stem cells are intrinsically different from the other types of cells studied in the literature, a seemingly straightforward test would be for the authors to test at least one class of their cells, e.g., posterior or mid trunk neural crest cells. in an in vitro system used by other researchers in in vitro studies. The goal would be to clarify whether there is something truly special about crest stem cell resistance to nuclear DNA damage, or whether this the finding described by the authors as surprising is simply due to the more rigid nature of the confinement used in previous in vitro studies. Previous studies have shown that it is indeed practical to culture zebrafish neural crest cells (e.g., PMID: 24326414, PMID: 28832322). Although this type of evaluation is not essential for verifying validity of the findings reported here, it would greatly enhance the impact and importance of this study for the field in general.

6. Wherever there is a linear scale on the Y axis where the origin does not start at zero, the authors need to clarify that they are focusing on a particular portion of the scale by using a double diagonal line to interrupt the Y axis, or some other indication that the scale does not start at zero.

Less major points:
7. Although the chondroitin sulfate proteoglycan neurocan can bind hyaluronan as stated, it can also bind tenascins, cell surface receptors such as N-CAM, and HSPGs. Therefore, its localisation does not provide evidence concerning the presence of only hyaluronan, since it is itself a extracellular proteoglycan that can link to a variety of other molecules and even cell surfaces. This point needs to be clearer, because otherwise a more specific marker for HA such as its binding protein HABP would be needed. This point could be resolved by appropriate text alterations.

8. There is a noticeable discrepancy between the data shown in Figure 1C for the differences in mean nuclear circularity between pre migratory and migratory cells compared to the data shown for the controls in Figure 3E. The text does not appear to indicate from which stage and anatomical location these results were obtained. Were these results pooled from anterior, mid, and posterior tissues, or from just one of these regions? It is also a little puzzling that the authors change from using box plots to linked individual data points in Figure 3 E and J. Was there some reason for this change?

9. Concerning point #1 above and lines 174-176, the collagen 1a1a result is interesting in suggesting the possibility of more collagen matrix resistance and confinement in posterior tissues, which might be due to anatomical location or to the fact that the tissues are older. Although not absolutely essential, the authors would ideally have examined whether the collagen is fibrillar, since presence of a single collagen subunit might not indicate the presence of a collagen matrix. Although EM (which they do perform) or SEM could provide the answer, this point is not crucial except as a possible explanation of the discrepancies noted in point #1.

10. Although it may not be practical for the authors to test this possibility, a theoretical possibility is that the age of the embryo might change the nature of the local microenvironment in terms of local spaces/confinement, and even the distensibility of nuclei. Although experimentally evaluating and ruling out these possibilities may not be practical, the authors should ideally discuss these possibilities. For example, at stage 30ss, is there evidence for collagen starting to appear in anterior regions?

11. Have the authors tested that all the antibodies they list actually cross-react with zebrafish antigens?

12. On line 143, the authors refer to a “basal membrane of fibronectin and laminin” but if they are referring to a basement membrane, the main components are collagen IV and laminin, and fibronectin is bound peripherally.

13. Very minor: In the legend to Figure 1, the author should ideally define the term”ss” for non-experts, since the use of somite-based staging compared to days post fertilization does seem more precise but could be confusing.

14. Very minor: There are only quite rare typos or confusing wording in this carefully prepared manuscript, e.g., line 362 “trough”

Reviewer #2 (Remarks to the Author):

The authors have submitted a well-written manuscript describing the use of the Zebrafish embryo neural crest to study nuclear deformation during migration through dense environments in an in vivo model. The authors take advantage of the excellent intravital imaging conditions in Zebrafish embryos and the natural variation in confinement along the neural crest antero-posterior axis, and the authors make clever use of genetic manipulation and laser ablation to further modulate the degree of confinement encountered by the neural crest cells. Using this model system, the authors convincingly demonstrate that whereas cells in the cranial neural crest show little nuclear deformation as they migrate in a largely unconfined environment, cells in the trunk neural crest encounter a more confined environment due to the presence of somite cells and consequently exhibit transient large nuclear deformations. The authors further report that despite the large nuclear deformations, the migrating neural crest cells do not encounter extensive nuclear envelope rupture or increased DNA damage.

The analysis of the spatial variation in confinement and the nuclear deformation during the migration is convincing and provides further support for the idea that migration through tight interstitial spaces results in large nuclear deformation. Other

findings, however, including the reported increase in nuclear pore size, decreased DNA damage, and changes in nuclear envelope composition, appear more preliminary and will require additional experiments and analyses to fully support the authors' conclusions.

The authors must address the following comments:

1) The authors report only a very small (~5%) change in the nuclear and cytoplasmic intensity of the nls-emGFP reporter and conclude that this change is not due to nuclear envelope rupture but arises from increased expression of the nls-emGFP over time and the leakage of the reporter through more permeable, stretched nuclear pores. This reviewer does not doubt the absence of extensive nuclear envelope rupture, and Stephen Young and Loren Fong's group had already described previously that migrating neurons in the mouse brain only exhibit nuclear envelope rupture upon deletion of lamin B1 or B2 (Chen et al. PNAS 2019, PMID 31796586). However, the nls-emGFP reporter and the BAF-mCherry reporters used here are not sufficiently characterized or lack relevant positive controls to fully interpret the findings and demonstrate that they have sufficient sensitivity to detect nuclear envelope ruptures. The authors show cytoplasmic localization of the nls-emGFP in mitotic cells, but in this case the nuclear envelope breaks down completely and chromatin becomes condensed, which is very different than the scenario of interphase nuclear envelope rupture, where holes in the nuclear membranes are only a few 100nm in diameter and chromatin is not condensed, which could affect interactions of the reporter with chromatin. The authors should demonstrate that their nls-emGFP reporter can reliably detect interphase nuclear envelope rupture, e.g., using laser ablation to damage the nuclear envelope or compressing cells in vitro. Similar assays should be used to validate the BAF-mCherry reporter. Furthermore, since fluorescence intensity measurements are sensitive to image settings, photobleaching, and changes in protein expressions, the authors should consider plotting the ratio of nuclear to cytoplasmic nls-emGFP, thus making it independent of the absolute intensity levels.

2) The authors attribute the changes in nuclear nls-emGFP intensity to stretch induced opening of nuclear pores, citing data from Suppl. Figure 4F-G as support. However, the reported difference in nuclear pore width is very small, less than 5 nm (Suppl. Fig. 4G), and appears well below what can be reliable measured based on the representative EM images in panel F", where the pore channel boundaries are not well delineated. Furthermore, the statistical significance appears to result only from the effect of pooling a large number of pores, all from a single embryo, which does not account for variation from experiment to experiment, sample prep, or embryo-to-embryo variability. Furthermore, the authors do not provide any functional evidence demonstrating increased nuclear transport through the pores under the confined conditions. The authors should either revise the text and soften their conclusions, or provide more thorough experimental data and more robust statistical analysis to support their interpretations.

3) There are similar concerns regarding the analysis of the DNA damage. Although the data presented in the manuscript suggest that the confined migration does not lead to increased damage, the interpretation that DNA damage DECREASES is less well supported. The positive control for the 53BP1 marker is based only on a single representative image (Suppl. Fig. S5A) and lacks quantification. Furthermore, the differences in DNA damage reported for the migratory cells based on gH2AX staining (Fig. 5B) and 53BP1 imaging (Fig. 5F') are quite small and might only be statistically significant by pooling a large number of cells, which does not account for experiment-to-experiment or embryo-to-embryo variability. It is also possible that migration, regardless of confinement, alters the rate of DNA damage. To address this possibility, the authors should also quantify DNA damage in premigratory and migratory cranial neural crest cells.

4) The authors hypothesize that the lack of nuclear envelope rupture and DNA damage can be attributed to the reduced Lamin B2 expression and altered distribution of LAP2beta. While elucidating the complete mechanism would be beyond the scope of this manuscript, the current findings appear quite preliminary and insufficient to support the authors' interpretation. First, the authors should provide a more detailed quantification of the expression levels lamin A/C, lamin B1, and lamin B2, instead of only presenting quantitative data for lamin B2 (Fig. 6B). These data should also include cranial neural crest cells to show whether they also exhibit changes in lamin B2 levels, despite the less confined environment. Since immunofluorescence labeling of lamins is sensitive to epitope masking or conformational changes (PMID 26301768 and 36809122), the authors should confirm the finding of reduced lamin B2 labels using orthogonal approaches. Furthermore, given that previous studies examining neuronal migration in the mouse brain found that deletion of lamin B1 and B2 increased, rather than decreased, nuclear envelope rupture, the authors should provide further support for their hypothesis, e.g., by testing whether overexpressing or depleting lamin B2 increases nuclear envelope rupture in the trunk neural crest cells. Lastly, the authors mention that lamin A/C is only found in some somite cells, but the figure referenced to support this statement (Fig. S6A) does not include any specific label to identify the cells with high lamin A/C levels as somite cells.

5) Related to the above comment, the authors' sole focus on LAP2beta is not sufficiently justified, and the authors should examine additional nuclear envelope proteins to determine whether the observed redistribution is specific to LAP2beta or reflects broader changes in nuclear envelope organization. The authors should particularly consider other nuclear envelope proteins that have been implicated in modulating nuclear envelope rupture, such as some of the candidates identified in a recent high-content screen by the group of Emily Hatch (PMID 38472343).

6) The authors should explore other mechanisms that might protect the neural crest cells from DNA damage and NE rupture, such as the role of BMP and WNT signaling, as alluded to in the discussion.

7) It appears that the trunk neural crest cells already exhibit more irregularly shaped nuclei in the premigration state (Fig 1C,D). The authors should comment on this and provide potential explanations, including whether this suggests differences in the physical environment or in the cells themselves.

8) In the description of the extracellular environment encountered by the trunk neural crest cells, the authors focus only on the role of collagen, particularly Col1a1a (Fig. 1C, D), while also mentioning the presence of hyaluronic acid (Fig. S2B, Fig. 2C). The authors should also comment on the presence of other extracellular matrix molecules that could contribute to the confinement, such as laminin, fibronectin, etc.

9) In Figure 2D and 2F, as well as the corresponding explanations in the methods, figure legends, and results sections, the authors present inter-tissue spaces as a linear distances (i.e., measured in µm). While this is a useful metric, the authors should also provide information on the other dimensions, or the cross-sectional area of the interstitial spaces, which might be a better predictor of cell behavior than a one-dimensional measurement.

10) For Figure 5B, the authors applied a Kruskal-Wallis test to test the comparison of the mean gamma-H2AX intensity between the premagratory and migratory neural crest cells. As the comparison is between two groups, the authors should use another more appropriate statistical test, such as a Mann-Whitney U test.

Minor comments

11) The authors should better acknowledge previous in vivo studies that demonstrated increased nuclear deformation, nuclear envelope rupture, and DNA damage. In addition to the Denais et al. and Raab et al. Science 2016 publications, which are cited only for their in vitro findings, the authors should include work by Abby Dernburg's group on nuclear deformation and nuclear rupture during oogenesis in C elegans (PMID 37436986), work by Stephen Young and Loren Fong in mouse neuronal migration (PMID 34161290 and 31796586), and live imaging in human blastocysts by Nicolas Plachta's group (PMID 37413989).

12) Page 5, Line 181. It appears that the beginning of the sentence is missing some works, which have been accidentally replaced by "ts". Please correct the sentence.

13) Page 5, line 185. The authors should comment on whether the spt mutant also affects neural crest cells, or only somite cells.

14) Page 5, line 193. The authors use the acronym EVL without defining it. Please revise the sentence to provide a definition.

Reviewer #3 (Remarks to the Author):

The manuscript studied the adaptive migration of trunk neural crest cells in zebrafish embryo as they navigate through narrow passageways between the neural tube and somite. The authors observed dynamic and significant nuclei deformation during this process, which is responsive to tissue confinement. Such deformation led to stretches of the nuclear envelope, expansion of the nuclear pores, but not rapture of the nuclear envelope or DNA damage. The protection from DNA damage likely results from relaxed nuclear envelope by decreased LaminB2 levels and decreased chromatin stress by depletion of Lap2beta from the leading edge of the nuclei.

While the concept of adaptive migration under confinement and nuclear deformation is not new, detailed analysis of this process in vivo is novel. This study provides insights into how neural crest cells adapt to tissue confinement without increasing DNA damage, in contrast to rupture of nuclear envelope in T-cell migration in highly confined in vitro conditions and increased genome instability during cancer cell metastasis.

The zebrafish trunk neural crest cell is a good model to study adaptive migration in vivo. The design of the study is logical and appropriate, using validated approaches and necessary controls are included. The quality of data is good in general, and the results are described clearly. Statistical analyses were performed when needed. Sufficient references are included. The conclusions are reliable.

There are some errors and minor issues noticed.

Figure 1, the definition of "premigratory zone" is unclear. It is more of NCCs before entering the narrow space underneath the somite rather than before NCCs initiate migration.

Figure 1 and S2, is the nuclear diameter the length of the short axis? The size of nuclei appears less than 10um (scale bar) in Fig.1A but measured as >= 10um in FigS2D.

Figure 3, in spt mutant, while the inter-tissue space is significantly wider, the effect on nuclei circularity or aspect ratio was not obvious. Here, pairwise comparison was performed. Is it because the sample size is smaller? Also, are the measurements done at mid-trunk level or posterior-trunk level? This is not indicated.

Figure 3H, in the ablated region, despite wide opening space, there does not seem to be increased number of NCCs migrating into the region. A discussion of whether tissue confinement influences cell entry or migration speed will be helpful.

Figure 4C’, D’ and Figure S4C’, the use of different scales for nuclear and cytosolic GFP intensity is unnecessary and confusing.

Figure 5A-B. It is surprising that in addition to not having an increased DNA damage, NCCs under confined migration show decreased DNA damage. The manuscript later discussed potential mechanisms to reduce stress, but why DNA damage is even less than cells without confinement stress? Is cell-cycle delay sufficient for lower DNA damage? Is this decrease in DNA damage also observed in migrating neural crest cells at cranial/vagal levels where there is no confinement or in embryos with ablated or mutated somites? Also, why premgratory and migrating NCCs have a significant level of phosphorylated gammaH2AX comparing to neighboring mesenchymal cells? In text, line 296, “compare pink arrows and arrowheads”, should arrowheads be yellow arrows?

Figure 5D, why compare nuclear circularity prior to 53BP1 foci appearance in premigratory and migratory crest cells? No difference here may mean that DNA damage appears at a certain pattern (deformation of nuclei). However, I think the point the authors want to make here is that the foci appear randomly. This should be demonstrated by a broad distribution of circularity index.

Figure 6. The polarized distribution of Lap2beta was not very clear. It seems be a general decrease of signal levels in highly deformed/stretched nuclei. When there is blebbing of nuclei, due to the stretching of the nuclear envelope, it may naturally be a decrease in the concentration of proteins associated with the envelope. This is not distinguished from an active polarization event. In the figure legend for Fig6C, “white arrowheads” should be “white arrows”.

Line 50, “device to for cells”
Line 130, “Fig1C, D, arrows”, there are no arrows in Fig1C, D.
Line 181, the first sentence in the paragraph needs revising.
Figure S3C, the label was messed up.

GUIDELINES FOR MANUSCRIPT SUBMISSION TO NATURE CELL BIOLOGY

READABILITY OF MANUSCRIPTS – Nature Cell Biology is read by cell biologists from diverse backgrounds, many of whom are not native English speakers. Authors should aim to communicate their findings clearly, explaining technical jargon that might be unfamiliar to non-specialists, and avoiding non-standard abbreviations. Titles and abstracts should concisely communicate the main findings of the study, and the background, rationale, results and conclusions should be clearly explained in the manuscript in a manner accessible to a broad cell biology audience. Nature Cell Biology uses British spelling.

MANUSCRIPT FORMAT – please follow the guidelines listed in our Guide to Authors regarding manuscript formats at Nature Cell Biology.

TITLE – should be no more than 100 characters including spaces, without punctuation and avoiding technical terms, abbreviations, and active verbs..

AUTHOR NAMES – should be given in full.

AUTHOR AFFILIATIONS – should be denoted with numerical superscripts (not symbols) preceding the names. Full addresses should be included, with US states in full and providing zip/post codes. The corresponding author is denoted by: "Correspondence should be addressed to [initials]."

ABSTRACT AND MAIN TEXT – please follow the guidelines that are specific to the format of your manuscript, as listed in our Guide to Authors (http://www.nature.com/ncb/pdf/ncb_gta.pdf) Briefly, Nature Cell Biology Articles, Resources and Technical Reports have 3500 words, including a 150 word abstract, and the main text is subdivided in Introduction, Results, and Discussion sections. Nature Cell Biology Letters have up to 2500 words, including a 180 word introductory paragraph (abstract), and the text is not subdivided in sections.

ACKNOWLEDGEMENTS – should be kept brief. Professional titles and affiliations are unnecessary. Grant numbers can be listed.

AUTHOR CONTRIBUTIONS – must be included after the Acknowledgements, detailing the contributions of each author to the paper (e.g. experimental work, project planning, data analysis etc.). Each author should be listed by his/her initials.

FINANCIAL AND NON-FINANCIAL COMPETING INTERESTS – the authors must include one of three declarations: (1) that they have no financial and non-financial competing interests; (2) that they have financial and non-financial competing

interests; or (3) that they decline to respond, after the Author Contributions section. This statement will be published with the article, and in cases where financial and non-financial competing interests are declared, these will be itemized in a web supplement to the article. For further details please see https://www.nature.com/licenceforms/nrg/competing-interests.pdf.

REFERENCES – are limited to a total of 70 for Articles, Resources, Technical Reports; and 40 for Letters. This includes references in the main text and Methods combined. References must be numbered sequentially as they appear in the main text, tables and figure legends and Methods and must follow the precise style of Nature Cell Biology references. References only cited in the Methods should be numbered consecutively following the last reference cited in the main text. References only associated with Supplementary Information (e.g. in supplementary legends) do not count toward the total reference limit and do not need to be cited in numerical continuity with references in the main text. Only published papers can be cited, and each publication cited should be included in the numbered reference list, which should include the manuscript titles. Footnotes are not permitted.

METHODS – Nature Cell Biology publishes methods online. The methods section should be provided as a separate Word document, which will be copyedited and appended to the manuscript PDF, and incorporated within the HTML format of the paper.

Methods should be written concisely, but should contain all elements necessary to allow interpretation and replication of the results. As a guideline, Methods sections typically do not exceed 3,000 words. The Methods should be divided into subsections listing reagents and techniques. When citing previous methods, accurate references should be provided and any alterations should be noted. Information must be provided about: antibody dilutions, company names, catalogue numbers and clone numbers for monoclonal antibodies; sequences of RNAi and cDNA probes/primers or company names and catalogue numbers if reagents are commercial; cell line names, sources and information on cell line identity and authentication. Animal studies and experiments involving human subjects must be reported in detail, identifying the committees approving the protocols. For studies involving human subjects/samples, a statement must be included confirming that informed consent was obtained. Statistical analyses and information on the reproducibility of experimental results should be provided in a section titled “Statistics and Reproducibility”.

All Nature Cell Biology manuscripts submitted on or after March 21 2016 must include a Data availability statement as a separate section after Methods but before references, under the heading "Data Availability”. . For Springer Nature policies on data availability see http://www.nature.com/authors/policies/availability.html; for more information on this particular policy see http://www.nature.com/authors/policies/data/data-availability-statements-data-citations.pdf. The Data availability statement should include:

• Accession codes for primary datasets (generated during the study under consideration and designated as "primary accessions") and secondary datasets (published datasets reanalysed during the study under consideration, designated as "referenced accessions"). For primary accessions data should be made public to coincide with publication of the manuscript. A list of data types for which submission to community-endorsed public repositories is mandated (including sequence, structure, microarray, deep sequencing data) can be found here http://www.nature.com/authors/policies/availability.html#data.

• Unique identifiers (accession codes, DOIs or other unique persistent identifier) and hyperlinks for datasets deposited in an approved repository, but for which data deposition is not mandated (see here for details http://www.nature.com/sdata/data-policies/repositories).

• At a minimum, please include a statement confirming that all relevant data are available from the authors, and/or are included with the manuscript (e.g. as source data or supplementary information), listing which data are included (e.g. by figure panels and data types) and mentioning any restrictions on availability.

• If a dataset has a Digital Object Identifier (DOI) as its unique identifier, we strongly encourage including this in the Reference list and citing the dataset in the Methods.

We recommend that you upload the step-by-step protocols used in this manuscript to protocols.io. More details can found at https://www.protocols.io/help/publish-articles.

DISPLAY ITEMS – main display items are limited to 6-8 main figures and/or main tables for Articles, Resources, Technical Reports; and 5 main figures and/or main tables for Letters. For Supplementary Information see below.

FIGURES – Colour figure publication costs $600 for the first, and $300 for each subsequent colour figure. All panels of a multi-panel figure must be logically connected and arranged as they would appear in the final version. Unnecessary figures and figure panels should be avoided (e.g. data presented in small tables could be stated briefly in the text instead).

All imaging data should be accompanied by scale bars, which should be defined in the legend.
Cropped images of gels/blots are acceptable, but need to be accompanied by size markers, and to retain visible background signal within the linear range (i.e. should not be saturated). The boundaries of panels with low background have to be demarked with black lines. Splicing of panels should only be considered if unavoidable, and must be clearly marked on the figure, and noted in the legend with a statement on whether the samples were obtained and processed simultaneously. Quantitative comparisons between samples on different gels/blots are discouraged; if this is unavoidable, it should only be

performed for samples derived from the same experiment with gels/blots were processed in parallel, which needs to be stated in the legend.

Figures should be provided at approximately the size that they are to be printed at (single column is 86 mm, double column is 170 mm) and should not exceed an A4 page (8.5 x 11"). Reduction to the scale that will be used on the page is not necessary, but multi-panel figures should be sized so that the whole figure can be reduced by the same amount at the smallest size at which essential details in each panel are visible. In the interest of our colour-blind readers we ask that you avoid using red and green for contrast in figures. Replacing red with magenta and green with turquoise are two possible colour-safe alternatives. Lines with widths of less than 1 point should be avoided. Sans serif typefaces, such as Helvetica (preferred) or Arial should be used. All text that forms part of a figure should be rewritable and removable.

We accept files from the following graphics packages in either PC or Macintosh format:

- For line art, graphs, charts and schematics we prefer Adobe Illustrator (.AI), Encapsulated PostScript (.EPS) or Portable Document Format (.PDF). Files should be saved or exported as such directly from the application in which they were made, to allow us to restyle them according to our journal house style.

- We accept PowerPoint (.PPT) files if they are fully editable. However, please refrain from adding PowerPoint graphical effects to objects, as this results in them outputting poor quality raster art. Text used for PowerPoint figures should be Helvetica (preferred) or Arial.

- We do not recommend using Adobe Photoshop for designing figures, but we can accept Photoshop generated (.PSD or .TIFF) files only if each element included in the figure (text, labels, pictures, graphs, arrows and scale bars) are on separate layers. All text should be editable in 'type layers' and line-art such as graphs and other simple schematics should be preserved and embedded within 'vector smart objects' - not flattened raster/bitmap graphics.

- Some programs can generate Postscript by 'printing to file' (found in the Print dialogue). If using an application not listed above, save the file in PostScript format or email our Art Editor, Allen Beattie for advice (a.beattie@nature.com).

Regardless of format, all figures must be vector graphic compatible files, not supplied in a flattened raster/bitmap graphics format, but should be fully editable, allowing us to highlight/copy/paste all text and move individual parts of the figures (i.e. arrows, lines, x and y axes, graphs, tick marks, scale bars etc.). The only parts of the figure that should be in pixel raster/bitmap format are photographic images or 3D rendered graphics/complex technical illustrations.

All placed images (i.e. a photo incorporated into a figure) should be on a separate layer and independent from any superimposed scale bars or text. Individual photographic images must be a minimum of 300+ DPI (at actual size) or kept constant from the original picture acquisition and not decreased in resolution post image acquisition. All colour artwork should be RGB format.

EXTENDED DATA FIGURES - When re-submitting your manuscript, please ensure that any supplementary figures and tables that are crucial to the manuscript's conclusions are converted into Extended Data figures and tables to increase visibility of these data. Extended Data figures and tables are online-only (present in the online PDF and full-text HTML versions of the paper), peer-reviewed display items that provide essential background to the article but are not included in the main article due to space constraints. A maximum of ten Extended Data display items (figures and tables) is permitted.

FIGURE LEGENDS – must not exceed 350 words for each figure to allow fit on a single printed NCB page together with the figure. They must include a brief title for the whole figure, and short descriptions of each panel with definitions of the symbols used, but without detailing methodology.

TABLES – main tables should be provided as individual Word files, together with a brief title and legend. For supplementary tables see below.

SUPPLEMENTARY INFORMATION – Supplementary information is material directly relevant to the conclusion of a paper, but which cannot be included in the printed version in order to keep the manuscript concise and accessible to the general reader. Supplementary information is an integral part of a Nature Cell Biology publication and should be prepared and presented with as much care as the main display item, but it must not include non-essential data or text, which may be removed at the editor's discretion. All supplementary material is fully peer-reviewed and published online as part of the HTML version of the manuscript. Supplementary Figures and Supplementary Notes are appended at the end of the main PDF of the published manuscript.

Supplementary items should relate to a main text figure, wherever possible, and should be mentioned sequentially in the main manuscript, designated as Supplementary Figure, Table, Video, or Note, and numbered continuously (e.g. Supplementary Figure 1, Supplementary Figure 2, Supplementary Table 1, Supplementary Table 2 etc.).

Unprocessed scans of all key data generated through electrophoretic separation techniques need to be presented in a supplementary figure that should be labelled and numbered as the final supplementary figure, and should be mentioned in every relevant figure legend. This figure does not count towards the total number of figures and is the only figure that can be displayed over multiple pages, but should be provided as a single file, in PDF or TIFF format. Data in this figure can be displayed in a relatively informal style, but size markers and the figures panels corresponding to the presented data must be

indicated.

The total number of Supplementary Figures (not including the “unprocessed scans” Supplementary Figure) should not exceed the number of main display items (figures and/or tables (see our Guide to Authors and March 2012 editorial http://www.nature.com/ncb/authors/submit/index.html#suppinfo; http://www.nature.com/ncb/journal/v14/n3/index.html#ed). No restrictions apply to Supplementary Tables or Videos, but we advise authors to be selective in including supplemental data.

Each Supplementary Figure should be provided as a single page and as an individual file in one of our accepted figure formats and should be presented according to our figure guidelines (see above). Supplementary Tables should be provided as individual Excel files. Supplementary Videos should be provided as .avi or .mov files up to 50 MB in size. Supplementary Figures, Tables and Videos much be accompanied by a separate Word document including titles and legends.

GUIDELINES FOR EXPERIMENTAL AND STATISTICAL REPORTING

REPORTING REQUIREMENTS – We are trying to improve the quality of methods and statistics reporting in our papers. To that end, we are now asking authors to complete a reporting summary that collects information on experimental design and reagents. The Reporting Summary can be found here https://www.nature.com/documents/nr-reporting-summary.pdf)If you would like to reference the guidance text as you complete the template, please access these flattened versions at http://www.nature.com/authors/policies/availability.html.

STATISTICS – Wherever statistics have been derived the legend needs to provide the n number (i.e. the sample size used to derive statistics) as a precise value (not a range), and define what this value represents. Error bars need to be defined in the legends (e.g. SD, SEM) together with a measure of centre (e.g. mean, median). Box plots need to be defined in terms of minima, maxima, centre, and percentiles. Ranges are more appropriate than standard errors for small data sets. Wherever statistical significance has been derived, precise p values need to be provided and the statistical test used needs to be stated in the legend. Statistics such as error bars must not be derived from n<3. For sample sizes of n<5 please plot the individual data points rather than providing bar graphs. Deriving statistics from technical replicate samples, rather than biological replicates is strongly discouraged. Wherever statistical significance has been derived, precise p values need to be provided and the statistical test stated in the legend.

Information on how many times each experiment was repeated independently with similar results needs to be provided in the legends and/or Methods for all experiments, and in particular wherever representative experiments are shown.

We strongly recommend the presentation of source data for graphical and statistical analyses as a separate Supplementary Table, and request that source data for all independent repeats are provided when representative experiments of multiple independent repeats, or averages of two independent experiments are presented. This supplementary table should be in Excel format, with data for different figures provided as different sheets within a single Excel file. It should be labelled and numbered as one of the supplementary tables, titled “Statistics Source Data”, and mentioned in all relevant figure legends.

--------- Please don't hesitate to contact NCB@nature.com should you have queries about any of the above requirements ---------

Version 1:

Decision Letter:

Our ref: NCB-A58386A

17th June 2026

Dear Dr. Scarpa,

Thank you for submitting your revised manuscript "In vivo DNA damage protection during cell migration across confining embryonic tissue environments" (NCB-A58386A). It has now been seen by the original referees and their comments are below. The reviewers find that the paper has improved in revision, and therefore we'll be happy in principle to publish it in Nature Cell Biology, pending minor revisions to satisfy the referees' final requests and clarify statistical anlyses; and to comply with our editorial and formatting guidelines.

The current version of your manuscript is in a PDF format, so please email us a copy of the file in an editable format (Microsoft Word or LaTex)-- we can not proceed with PDFs at this stage.

We are now performing detailed checks on your paper and will send you a checklist detailing our editorial and formatting requirements in a couple of weeks. Please do not upload the final materials and make any revisions until you receive this additional information from us.

Thank you again for your interest in Nature Cell Biology Please do not hesitate to contact me if you have any questions.

Sincerely,
Daryl

Daryl Jason Verzosa David, PhD

Senior Editor, Nature Cell Biology
Advisory Editor, npj Biological Physics and Mechanics
Nature Portfolio

Heidelberger Platz 3, 14197 Berlin, Germany
Email: daryl.david@nature.com
ORCID: https://orcid.org/0000-0002-9253-4805

Reviewer #1 (Remarks to the Author):

This re-review of resubmitted manuscript NCB-A58386A shows that the authors have responded directly and conscientiously to all of my concerns and suggestions, with intriguing new information about the notable characteristics of neural crest cells with respect to DNA damage protection. The results help to advance the field conceptually, so I am now happy to support its publication with congratulations to the authors on their fine work.

Reviewer #2 (Remarks to the Author):

The authors have submitted a revised manuscript that includes additional experiments and several updates to the text that have substantially improved the manuscript. The authors are commended for the thorough revision and inclusion of additional data. The new data and revised text address many of the previous concerns and convincingly show the extend of nuclear deformation during trunk neurocrest cell migration in vivo and the lack of nuclear envelope rupture and DNA damage during this confined migration.

Nonetheless, some of the originally stated concerns remain, particularly regarding the statistical analysis, and the new RNA-seq data and experiments using lamin B2 depletion/overexpression raise some new questions.

The authors need to address the following concerns:

1) Many of the reported effects are extremely small and often appear to only reach statistical significance by pooling a large number of cells or nuclear pores, which is prone to results in statistically significant differences that are not biologically relevant. For example, the reported difference in the nuclear pore diameter (Fig. 4I), the differences in the dynamics of nuclear deformation (Fig. 6F), or the 53BP1 accumulation upon lamin B2 overexpression (Fig. 7B) have effect sizes that are well below the variability between individual measurements and possibly between individual embryos. Based on previous comments, the authors now show the mean values for individual embryos in some figure panels (e.g., Fig. 5B, Suppl. Fig. S7B, D), allowing for more robust interpretation, but this remains the exception. Most graphs still only show the individual data points, with the information about the number of embryos and independent experiments only listed in the figure legend, and it is unclear how this information was used in the statistical analysis. To increase the robustness of the findings, the authors should show superplots (i.e., individual data points plus the mean values for individual embryos) in all figures and perform statistical analyses that consider the embryo-to-embryo and experiment-to-experiment variability and then update the text and conclusions accordingly. I do believe that the key findings about nuclear deformability and the lack of nuclear envelope rupture and DNA damage are robust, but many of the other, more subtle findings appear much less robust, which can affect the interpretation of the findings.

2) In the abstract and at the end of the introduction (lines 106-108), the authors highlight the role of lamin B2 as a key adaptive change in nuclear envelope composition to enable confined migration without experiencing DNA damage or nuclear envelope rupture. However, based on their new experiments using lamin B2 depletion or overexpression, it appears that the effect of changes in lamin B2 levels is much more subtle and primarily affects nuclear deformation without necessarily explaining the lack of nuclear envelope rupture or DNA damage. The authors should revise their text and soften their conclusions to reflect this.

3) The new photoactivation/RNA-seq experiments are very laudable and provide new insights. However, their interpretation is not entirely clear and likely not as straight forward as the authors make it appear. DNA damage response pathways are typically upregulated in response to DNA damage, or to facilitate improved DNA repair, and not to prevent DNA damage in the first place. Thus, an alternative interpretation might be that the cells experience subtle DNA damage insufficient for detection by fluorescence microscopy but sufficient to activate DNA damage response pathways. Another interpretation is that the reported changes represent developmental differences, given that the cells for the unconfined conditions were photoactivated and collected at a different time point (16 hpf) than the cells used for the confined migration conditions (23-24 hpf). A more direct comparison would be to photoactivate cells at the same time point, but using cells in different locations

along the migration path. In addition, to more directly test whether the increased expression of various DNA damage response pathways is indeed protective of DNA damage, the authors could test whether the tNC cells at the later stage are protected against other types of DNA damage, e.g., UV or chemically induced DNA damage. While a detailed analysis of the precise protective mechanisms is beyond the scope of the manuscript, without additional data it is difficult to justify the current interpretation that the upregulation of the DNA damage response pathways, or the depletion of lamin B2, is solely the reason for the lack of DNA damage during the trunk neural crest cell migration.

4) Lines 249-250. The authors state that "cells in the ablated region showed a more subtle decrease in nuclear circularity", referring to Fig. 3J. However, the data in Figure panel 3J still show a significant change in the mean circularity upon migratory transition in the ablated region ($p < 0.05$), and apart from two cells in the adjacent somites, which show very large changes, the effect size seems to be quite similar. The authors do not include any statistical analysis comparing the effect size between these two conditions, which would be necessary to justify the statement of a "more subtle decrease", and the conclusions appears to be based solely on a less significant p-value ($p < 0.05$ vs. $p < 0.001$) between the conditions, which is not a measure of the effect size and could simply be due to the smaller number of cells analyzed in the ablation group. The authors should either perform appropriate statistical tests to demonstrate a difference in effect size or revise their text accordingly.

5) Besides the concerns regarding the statistical analysis (see comment #1), this reviewer remains concerned that the SEM images to analyze changes in nuclear pore diameter are of insufficient quality to accurately determine the nuclear pore size to the extent to detect subtle differences of a few nm. While the authors have already made some changes to the text, the authors should either include TEM images to allow for more precise measurements (and conduct measurements by an observer blinded for condition) or better acknowledge this limitation.

Minor comments:

6) Line 285. The authors write that migratory trunk neurocrest cells "show a transient loss of NE integrity". This statement seems to contradict their subsequent findings that these cells do not show nuclear envelope rupture, based on the nls-emGFP and BAF-mCherry experiments. The authors should rephrase this statement to avoid any confusion.

Reviewer #3 (Remarks to the Author):

The authors have provided substantial additional work, which has improved the manuscript and addressed most of the comments from all three reviewers.

My only remaining concern, unfortunately, is that it seems that LaminB2, which is still described as one of the main mechanisms for low DNA damage in confined NC migration, plays a rather modest role in protecting cells from DNA damage. While LaminB2 manipulations leads to changes in nuclei deformation, this does not correlate to DNA damages.

From new data provided by the authors, several mechanisms contribute to the low DNA damage. 1) Tissues surrounding migrating NCCs are rather soft. 2) LaminB2 modulates the dynamics of nuclei deformation and nuclei deformation is relatively transient. 3) Multipotent NCCs seems to have a strong protective program and have a up-regulated DNA repair machinery.

These results weakened the importance of Lamin2B regulation in DNA damage protection.

INTERNAL USE ONLY AIP modules required: ["Clinical","Life Science"]

Version 2:

Decision Letter:

Dear Dr Scarpa,

I am pleased to inform you that your manuscript, "In vivo DNA damage protection during cell migration across confining embryonic tissue environments", has now been accepted for publication in Nature Cell Biology.

Thank you for sending us the final manuscript files to be processed for print and online production, and for returning the manuscript checklists and other forms. Your manuscript will now be passed to our production team who will be in contact with you if there are any questions with the production quality of supplied figures and text.

Over the next few weeks, your paper will be copyedited to ensure that it conforms to Nature Cell Biology style. Once your paper is typeset, you will receive an email with a link to choose the appropriate publishing options for your paper and our

Author Services team will be in touch regarding any additional information that may be required.

After the grant of rights is completed, you will receive a link to your electronic proof via email with a request to make any corrections within 48 hours. If, when you receive your proof, you cannot meet this deadline, please inform us at rjsproduction@springernature.com immediately.

You will not receive your proofs until the publishing agreement has been received through our system.

Due to the importance of these deadlines, we ask that you please let us know now whether you will be difficult to contact over the next month. If this is the case, we ask you provide us with the contact information (email, phone and fax) of someone who will be able to check the proofs on your behalf, and who will be available to address any last-minute problems.

If you have any questions about our publishing options, costs, Open Access requirements, or our legal forms, please contact ASJournals@springernature.com

Once your paper has been scheduled for online publication, the Nature press office will be in touch to confirm the details. An online order form for reprints of your paper is available at https://www.nature.com/reprints/author-reprints.html. All co-authors, authors' institutions and authors' funding agencies can order reprints using the form appropriate to their geographical region.

Publication is conditional on the manuscript not being published elsewhere and on there being no announcement of this work to any media outlet until the online publication date in Nature Cell Biology.

You may wish to make your media relations office aware of your accepted publication, in case they consider it appropriate to organize some internal or external publicity. Once your paper has been scheduled you will receive an email confirming the publication details. This is normally 3-4 working days in advance of publication. If you need additional notice of the date and time of publication, please let the production team know when you receive the proof of your article to ensure there is sufficient time to coordinate. Further information on our embargo policies can be found here: https://www.nature.com/authors/policies/embargo.html

Please note that *Nature Cell Biology* is a Transformative Journal (TJ). Authors may publish their research with us through the traditional subscription access route or make their paper immediately open access through payment of an article-processing charge (APC). Authors will not be required to make a final decision about access to their article until it has been accepted. Find out more about Transformative Journals

Authors may need to take specific actions to achieve compliance with funder and institutional open access mandates. If your research is supported by a funder that requires immediate open access (e.g. according to Plan S principles or the NIH public access policy) then you should select the gold OA route, and we will direct you to the compliant route where possible. Because authors warrant under our subscription licensing terms that they haven't committed to licensing any version of their article under a licence inconsistent with the terms of our agreement – including the applicable embargo period – publication under the subscription model isn't suitable for authors whose funders require no embargo.

To assist our authors in disseminating their research to the broader community, our SharedIt initiative provides you with a unique shareable link that will allow anyone (with or without a subscription) to read the published article. Recipients of the link with a subscription will also be able to download and print the PDF.

If your paper includes color figures, please be aware that in order to help cover some of the additional cost of four-color reproduction, Nature Portfolio charges our authors a fee for the printing of their color figures. Please contact our offices for exact pricing and details.

As soon as your article is published, you will receive an automated email with your shareable link.

If you have not already done so, we strongly recommend that you upload the step-by-step protocols used in this manuscript to protocols.io (https://protocols.io), an open online resource that allows researchers to share their detailed experimental know-how. All uploaded protocols are made freely available and are assigned DOIs for ease of citation. Protocols and Nature Portfolio journal papers in which they are used can be linked to one another, and this link is clearly and prominently visible in the online versions of both. Authors who performed the specific experiments can act as primary authors for the Protocol as they will be best placed to share the methodology details, but the Corresponding Author of the present research paper should be included as one of the authors. By uploading your Protocols onto protocols.io, you are enabling researchers to more readily reproduce or adapt the methodology you use, as well as increasing the visibility of your protocols and papers. You can also establish a dedicated workspace to collect your lab Protocols. Further information can be found at https://www.protocols.io/help/publish-articles.

You can use a single sign-on for all your accounts, view the status of all your manuscript submissions and reviews, access usage statistics for your published articles and download a record of your refereeing activity for the Nature Portfolio.

Nature Cell Biology encourages authors presenting evidence for cell, biological, molecular, and genetic interactions to consider communicating these findings using Biofactoid (https://biofactoid.org/). This tool helps users share a searchable representation of interactions (e.g. binding, gene expression, post-translational modification) between genes, gene products, or chemicals. Information added to Biofactoid, with author attribution, is shared on social media and public databases, such as Pathway Commons, where it can be discovered and analyzed in the context of a large and growing corpus of knowledge.

Please feel free to contact us if you have any questions.

With kind regards,
Daryl

Daryl Jason Verzosa David, PhD

Senior Editor, Nature Cell Biology
Advisory Editor, npj Biological Physics and Mechanics
Nature Portfolio

Heidelberger Platz 3, 14197 Berlin, Germany
Email: daryl.david@nature.com
ORCID: https://orcid.org/0000-0002-9253-4805

Click here if you would like to recommend Nature Cell Biology to your librarian
http://www.nature.com/subscriptions/recommend.html#forms

** Visit the Springer Nature Editorial and Publishing website at www.springernature.com/editorial-and-publishing-jobs for more information about our career opportunities. If you have any questions please click here.**

Open Access This Peer Review File is licensed under a Creative Commons Attribution 4.0 International License, which permits use, sharing, adaptation, distribution and reproduction in any medium or format, as long as you give appropriate credit to the original author(s) and the source, provide a link to the Creative Commons license, and indicate if changes were made.
In cases where reviewers are anonymous, credit should be given to 'Anonymous Referee' and the source.
The images or other third party material in this Peer Review File are included in the article's Creative Commons license, unless indicated otherwise in a credit line to the material. If material is not included in the article's Creative Commons license and your intended use is not permitted by statutory regulation or exceeds the permitted use, you will need to obtain permission directly from the copyright holder.
To view a copy of this license, visit https://creativecommons.org/licenses/by/4.0/

Reviewers' Comments:

Reviewer #1 (Remarks to the Author):

We thank the reviewers for their insightful comments on our manuscript. We are grateful for the constructive feedback of each of the reviewers, which has allowed us to improve the manuscript and clarify our findings.

In the revised manuscript we have extended our findings of neural crest (NC) in vivo migration in confined tissue environments and we have included the following new experimental results:

1) We show with two orthogonal approaches, with immunohistochemistry and a live reporter, that trunk NC do not accumulate DNA damage during confined migration across the antero-posterior body axis. We further provide a novel in vitro tNC assay to compare our in vivo findings with in vitro. We found that tNC do not accumulate DNA damage even in in vitro confined culture conditions
2) We explore the nuclear lamins in early embryonic tissues and provide a knockdown and tissue specific over expression of laminB2 in NC cells. Our new results suggest that regulation of laminB2 in migratory NC cells is important for the rapid recovery from the nuclear deformation events during migration. We show that over expression of laminB2 in migratory cell leads to small but significant increase of DNA damage accumulation in migratory cells.
3) By using a photoconversion and RNA sequencing approach to compare migratory (confined) and premigratory (non confined) cells we found that DNA repair related genes are the most significantly upregulated group in the migratory cells population. Together our new data supports that tNC cells are well protected from DNA damage during confined in vivo migration.

We have carefully addressed the points raised by the reviewers and here we provide point-by-point response below.

This manuscript provides novel, potentially interesting evidence that in vivo deformation of neural crest stem cell nuclei does not cause DNA damage even as the cells and nuclei undergo high deformation in very narrow tissue spaces. These findings are valuable in apparently contradicting previous purely in vitro studies, though the authors point out that these differences might be related to the stem cell nature of neural crest cells. They report that by careful measurements of the width of extracellular spaces from cranial to caudal the “level of tissue scale confinement increases along the antero-posterior axis” reflected in altered nuclear morphology; the latter is quantified to show significant nuclear deformation by measuring both nuclear circularity and aspect ratio. The authors use a genetic model in which tissue spaces are enlarged as well as simple laser ablation of adjacent tissue to experimentally prevent the nuclear deformation. They report that their neural crest cells are able to reduce DNA damage under confinement even with the loss of soluble intranuclear molecules resulting from nuclear pore enlargement. This protection is reportedly associated with localised reduction in nuclear lamin B2 levels when the cells are within confined spaces but not before and after this confinement. The authors posit the interesting concept that the neural crest cells show “dynamic adaptation to tissue confinement in vivo” without the DNA damage that most in the field would have expected with this extent of nuclear deformation.

This reviewer is enthusiastic about the approach used in this clearly written and presented study to test the role of confinement of migrating cells in vivo with the very interesting and potentially important finding that neural crest cells do not display the expected level of DNA damage after even major nuclear deformation. On the other hand, there are some substantive reservations. For example, the evidence for the statement that "level of tissue scale confinement increases along the antero-posterior axis" is weakened slightly by the quantitative posterior embryo data, and the evidence for "dynamic adaptation to tissue confinement in vivo" providing protection is weak. Overall, the authors will need to use revised text consistent with their data and to avoid the temptation to over-interpret in overly broad conclusions.

Major points:

1. I sympathize with the authors that there is an unfortunate discrepancy between the morphological data showing significantly lower nuclear circularity plus deformation with increasingly posterior localization (Figures 1C and Supplementary Fig. 2D) compared to the non-significant quantification of mean spaces in mid versus posterior tissues in Figure 2F. Yet the examples shown in Figures 2E and F show that there is closer spacing between tissues in posterior trunk tissues, whereas the quantitative data actually show no differences between mid and posterior trunk tissues, and if anything a very slight non-significant widening in posterior tissues. The authors need to choose images that match the quantitative data, regardless of the possible dilution of the simplicity of the story. Instead, there may well be other causes/reasons besides merely tissue space differences, such as differing elasticity or local matrix/tissue pore size in posterior regions. In fact, if there is more collagen posteriorly, that might explain the morphological data. If the authors wish to claim an increase in tissue scale confinement by pointing to the nuclear morphological data, they need to make this point clear, since measurements of tissue spaces do not show a further decrease in the posterior region of embryos.

We thank the reviewer for this comment. We agree that confinement *imposed from surrounding tissues* does not differ between mid-trunk and posterior trunk. As the reviewer is pointing out, we provided immunostaining data of Collagen1a1a (col1a1a) at different antero-posterior regions at the time of NC migration, and found that only in the posterior region col1a1a is enriched at the onset of NC migration. Col1a1a is especially enriched at the premigratory region (pink arrow head in cross-section), which can explain why NC cells are also more deformed in the premigratory region in the posterior region. We have now

1) Provided a quantification of col1a1a over the different regions of the embryonic trunk, which highlight the increase in col1a1a in posterior trunk (Figure S2H).
2) Rephrased and toned down our conclusions relative to figure 2 (see lines 188-190 of revised manuscript.
3) Replaced the posterior panels in Figure 2E.

2. The reported alterations in nuclear lamin localisation in Figure 6 during deformation initially seemed quite intriguing in support of the concept of dynamic adaptation to tissue confinement. However, there are problems with the data here and in the supplementary data for this figure.

First, even though the differences are highly statistically significant during deformation, the actual difference seems to be quite small, i.e., less than 5% because the Y axis scale is so expanded, with an entire 0.8 of the scale missing. Second, the authors do not show a statistical comparison of the nucleus front versus rear in the 'Before deformation' state. Finally, in Supplementary Figure 6C, there is an equally major, important and statistically notable decrease in localisation at the nucleus front during deformation of cranial when compared to more-posterior neural crest cells, yet the authors state in the text that the localisation "does not show a polarized localisation" - whereas the data show that there is an equally significant reverse localisation! These issues will need to be resolved by significantly toning down the claim and clarifying the findings in the text.

The reviewer here refers to quantifications of *Lap2β* dynamic localisation (Figure 6D, S6C in the first version of the manuscript,). We have now provided the same plot with the full Y-axis scale, provided the missing statistical tests, toned down and clarified the findings in the text (line 298). Of note, these are normalised values (see Methods section) so the values of individual data points spread around 1. Gray dashed bar has been added to the figure to highlight the normalisation. These results have now been moved to Figure S4H,J in the revised manuscript.

In addition, we have now revised all the plots and largely replaced them with full Y scale. Those line plots that still have truncated Y axis for the sake of better visualisation have been clearly marked in the panels and stated in the figure legends.

3. Figure 1 shows striking heterogeneity in the outlines of cells at the different levels of neural crest from cranial to posterior consistent with differences in confinement. The morphologies of cells can provide a good indication of the lateral forces on cells and nuclei, so a key question is whether the examples shown are representative of the shapes of cells at each of these different levels. For example, are the cranial neural crest cells really that strikingly varying in their protrusions compared to those at all the other levels, and are the mid-trunk neural crest cells so laterally compressed compared to those in the anterior and posterior neural crest regions? It would be much better for the authors to provide truly representative outlines of cell bodies both here and perhaps in expanded supplemental materials showing readers the range of cell morphologies at each of the different levels.

To clarify this point, we have now provided the plasma membrane channel for representative images of the NC cells in Figure 1A' in the Figure S1A. CNC cells migration is not as directional the trunk NC migration (PMID: 27210753) which likely reflect on the protrusion dynamics.

We further analysed the cell shape along the antero-posterior axis and although CNC cells circularity and aspect ratio are reduced in comparison of the tNC cells shapes, the difference is not significant. These new data are placed now in Figure S1B,C,D, and commented in the main text at line 146.

4. It is surprising to this reviewer that the quantified mean nuclear circularity of the neural crest cells shows disturbingly quite modest differences in effect size, albeit highly statistically significant. It would be useful, perhaps in supplementary material, for the authors to indicate the proportions of cells that show striking differences in morphology versus those that are

unaffected compared to evidence of DNA damage. The reason for this concern is to ask the important question: do cells with the largest differences in nuclear deformation still show no significant evidence of DNA damage?

Figure S1A-A' examples of nucleus circularity and aspect ratio curves over time for a single cell to highlight the fluctuating nature of the nuclear shape over time. The mean nuclear circularity values represented in figure 1C represent an average of all these circularity timepoints per each cell. Both low and high circularity values will be included in these averages, thus resulting in modest differences in effect size as highly deformed timepoints are offset by less deformed timepoints.

To highlight the maximum deformation events in different antero-posterior regions as well as in the different conditions in the revised manuscript, we also present an aligned circularity curve in order to compare the maximum deformation events. In the Figure S1F,F' we have now added a pink dashed line to highlight the mean circularity of the migratory NC to highlight how most of the migratory cells show significantly lower circularity levels during the migration.

Regarding the reviewers question about DNA damage, we have now carefully investigated possible correlation between the levels of nuclear deformation and DNA damage response using 53BP1mApple live reporter.
We first measured the mean 53BP1 peak intensity after the maximum deformation in all NC subpopulations (Figure 5D,5E,S5E) and did not detect an increase in 53BP1 mean intensity. We next performed a correlation analysis between the minimum circularity value and the immediate DNA damage response by calculating the mean 53BP1 peak intensity up to 60 min after, and could not detect correlation between the minimum circularity and 53BP1 intensity (Figure 5F, Figure S5F).
We have further provided similar analysis for the tNC in vitro (Figure 5K,L, Figure S5M,N) and for the tissue specific laminB2 over expression in migratory NC (Figure 7C,D). In all conditions, 53BP1 did not show an increase after the minimum circularity, nor was correlation between circularity levels and 53BP1 intensity detected.

As the circularity can also change gradually also before the nucleus reaches the maximum deformation (Figure S1E,F), we also checked whether we observe 53BP1 increase as a response to maximum rate of circularity change which we provide in this letter for the reviewers (Figure R1A). We did not detect an increase of 53BP1 over time as a response to the change of circularity, nor did we find a correlation between the rate of circularity change and 53BP1 intensity. We also performed similar analysis using the emGFP-53BP1 tissue specific reporter and could not detect a 53BP1 response after maximum change of rate of circularity (Figure R1A).

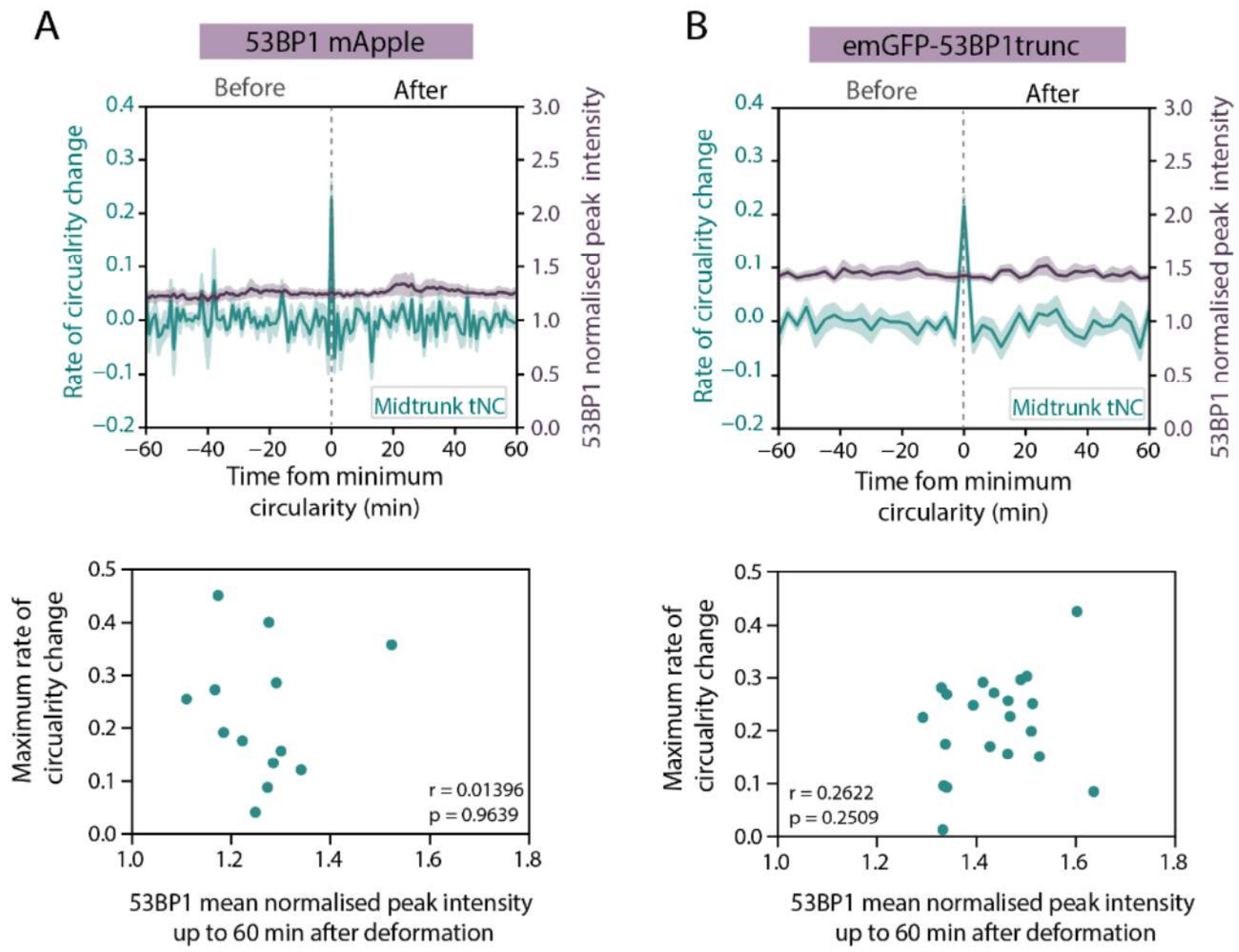

Figure R1.

A) Rate of nuclear circularity rate aligned to the maximum change and 53BP1mApple peak intensity (maximum intensity/mean intensity) change in mid tNC migratory cells (top) and correlation between maximum rate of change and 53BP1 mean peak intensity up to 60min after maximum change of circularity (bottom). n=13 cells from 6 embryos.

B) Rate of nuclear circularity rate aligned to the maximum change and emGFP-53BP1 peak intensity (maximum intensity/mean intensity) change in mid tNC migratory cells (top) and correlation between maximum rate of change and 53BP1 mean peak intensity up to 60min after maximum change of circularity (bottom). n=21 cells from 9 embryos. Mean and SEM presented. Pearson correlation applied, r and p values reported in the scatter plots (bottom).

Similarly, we did not appreciate such correlation for our fixed γH2AX antibody stainings (Supplementary Figure 5G).

Together, we can confidently state that despite increasing levels of tissue confinement and nuclear deformation across neural crest population we do not detect increased DNA damage.

We have now modified the Figure 5 to address better the question of DNA damage response to maximum nuclear deformation (lines 339-345) together with the new data plots (Figure 5E,F,K,L and S5E,F,M,N).

5. The findings presented in the study are important in providing an in vivo test of the role of confinement/tissue spaces in nuclear morphology and DNA damage. They are obviously more authentic biologically than the multiple in vitro studies published in recent literature. However, there is one potential major difference: tissues in vivo have variable boundaries and most importantly local elasticity and viscoelasticity. It is conceivable that the in vitro studies were misleading due to the nature of the confining material. To substantially enhance the importance of the findings in this study and to evaluate more rigorously the conclusion that neural crest stem cells are intrinsically different from the other types of cells studied in the literature, a seemingly straightforward test would be for the authors to test at least one class of their cells,

e.g., posterior or mid trunk neural crest cells in an in vitro system used by other researchers in in vitro studies. The goal would be to clarify whether there is something truly special about crest stem cell resistance to nuclear DNA damage, or whether this the finding described by the authors as surprising is simply due to the more rigid nature of the confinement used in previous in vitro studies. Previous studies have shown that it is indeed practical to culture zebrafish neural crest cells (e.g., PMID: 24326414, PMID: 28832322 cranial). Although this type of evaluation is not essential for verifying validity of the findings reported here, it would greatly enhance the impact and importance of this study for the field in general.

We thank the reviewer for this valuable suggestion. To measure the stiffness of Zebrafish trunk tissues, we carried out atomic force microscopy of thick, live Zebrafish mid-trunk cross-sections (Figure 5H-J). These findings highlight that, in line with previous AFM measurements (Yamaguchi et al, 2022 PMID: 35165417) tissues appear indeed soft (~0.4-0.5 kPa) compared to the PDMS material often used in microfabrication studies (PDMS stiffness ~1.3MPa PMID: 17653351). To test whether neural crest cells might experience DNA damage when confined within more rigid, non-deformable boundary conditions, we adapted a protocol for primary Zebrafish neural crest culture (PMID: 24326414) to trunk neural crest cells (see Methods in the revised manuscript, and Figure S5K) and we confined primary Zebrafish TNC in PDMS pillar forests with pillars spaced at a 3μm distance between each other. Nuclear shapes in these devices appear strongly deformed (Figure 5H,I, S5L,O-O'). Importantly, assessment of DNA damage using the mApple 53BP1 live reporter dynamics reveal that neural crest cells still do not incur DNA damage under stiff *in vitro* confinement (Figure 5,J,KL,). To answer the reviewers comment, tNC do not accumulate DNA damage neither in vivo nor in vitro under higher mechanical stress, supporting that NC might have specific properties to protect from accumulation of DNA damage.

6. Wherever there is a linear scale on the Y axis where the origin does not start at zero, the authors need to clarify that they are focusing on a particular portion of the scale by using a double diagonal line to interrupt the Y axis, or some other indication that the scale does not start at zero.

We have now largely replotted data to show full Y axis. In those panels where Y scale is still truncated for improved visualisation, the truncation is now clearly presented in the panel and stated in the figure legend.

Less major points:

7. Although the chondroitin sulfate proteoglycan neurocan can bind hyaluronan as stated, it can also bind tenascins, cell surface receptors such as N-CAM, and HSPGs. Therefore, its localisation does not provide evidence concerning the presence of only hyaluronan, since it is itself a extracellular proteoglycan that can link to a variety of other molecules and even cell surfaces. This point needs to be clearer, because otherwise a more specific marker for HA such as its binding protein HABP would be needed. This point could be resolved by appropriate text alterations.

The reporter we use (ss-NCan-GFP) is not a full length Neurocan-GFP, but the HA-binding domain only. This reporter has been carefully validated in Zebrafish and reflects HA distribution in live embryos (PMID: 28118600).

8. There is a noticeable discrepancy between the data shown in Figure 1C for the differences in mean nuclear circularity between pre migratory and migratory cells compared to the data shown for the controls in Figure 3E. The text does not appear to indicate from which stage and anatomical location these results were obtained. Were these results pooled from anterior, mid, and posterior tissues, or from just one of these regions? It is also a little puzzling that the authors change from using box plots to linked individual data points in Figure 3 E and J. Was there some reason for this change?

The control datasets for Figure 3E are control siblings of spadetail mutant embryos imaged in the mid-trunk region. We have now better clarified in the text that we investigate the spadetail mutant in the mid-trunk region (line 198-199), where somites are affected (PMID: 2911386, also cited in main text).

Figure 3E,J: we used linked individual data points here as we specifically address changes in nuclear shape for cells that transition from the premigratory region to under somite confinement in control versus perturbed somite conditions.

9. Concerning point #1 above and lines 174-176, the collagen 1a1a result is interesting in suggesting the possibility of more collagen matrix resistance and confinement in posterior tissues, which might be due to anatomical location or to the fact that the tissues are older. Although not absolutely essential, the authors would ideally have examined whether the collagen is fibrillar, since presence of a single collagen subunit might not indicate the presence of a collagen matrix. Although EM (which they do perform) or SEM could provide the answer, this point is not crucial except as a possible explanation of the discrepancies noted in point #1.

We have now clarified point 1 in the text and provided quantifications of col1a1a fluorescence (see lines 182-188 of revised manuscript, Supplementary Figure S2H).

10. Although it may not be practical for the authors to test this possibility, a theoretical possibility is that the age of the embryo might change the nature of the local microenvironment in terms of local spaces/confinement, and even the distensibility of nuclei. Although experimentally evaluating and ruling out these possibilities may not be practical, the authors should ideally discuss these possibilities. For example, at stage 30ss, is there evidence for collagen starting to appear in anterior regions?

It is possible that the age of embryo might change the nature of the local microenvironment and that as cells differentiate into specific fates this might lead to changes in nuclear envelope composition and mechanical properties. Indeed, whilst we do not detect Col1a1a in anterior regions at the stages of neural crest migration (14-18 hours post fertilisation) we do detect it at 26 hours post fertilisation (Figure R2). We add the representative image to this letter for clarification. However, the aim of this work was to interrogate how *migrating* cells cope with

their physiological environment, hence we chose to test timepoints at which neural crest populations undergo migration.

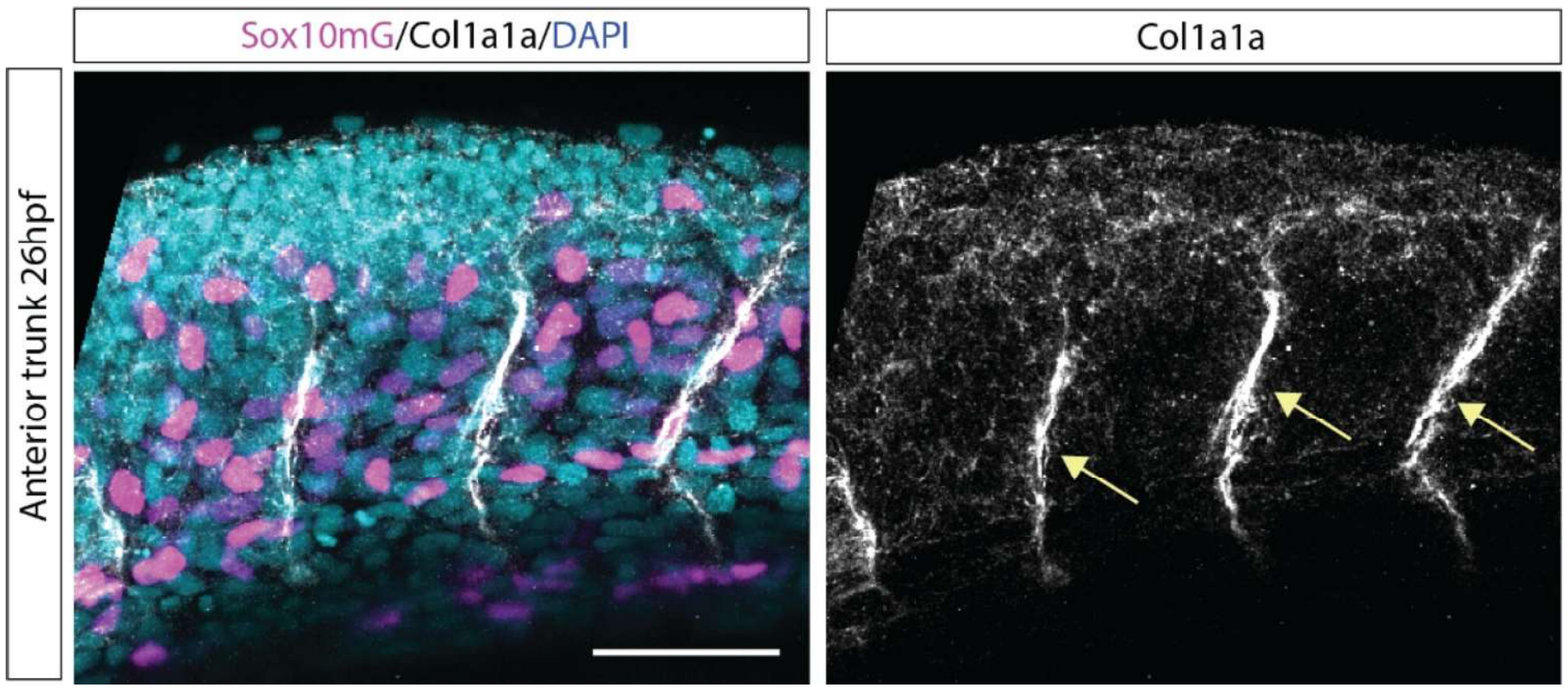

Figure R2.
Maximum projection of anterior trunk region at 26hpf when NC cells (magenta) have already migrated from the dorsal to ventral . Yellow arrows highlights the localisation of the collagen1a1a at the somite boundaries. Scalebar 50um.

11. Have the authors tested that all the antibodies they list actually cross-react with zebrafish antigens?

Yes. The relevant references list is already in the data reproducibility table.

12. On line 143, the authors refer to a "basal membrane of fibronectin and laminin" but if they are referring to a basement membrane, the main components are collagen IV and laminin, and fibronectin is bound peripherally.

This has now been edited as "lined with fibronectin and laminin" (line 155-156).

13. Very minor: In the legend to Figure 1, the author should ideally define the term"ss" for non-experts, since the use of somite-based staging compared to days post fertilization does seem more precise but could be confusing.

This has now been edited. We use the term hpf (hours post fertilization) though out the paper.

14. Very minor: There are only quite rare typos or confusing wording in this carefully prepared manuscript, e.g., line 362 "trough"

This has now been edited.

Reviewer #2 (Remarks to the Author):

The authors have submitted a well-written manuscript describing the use of the Zebrafish embryo neural crest to study nuclear deformation during migration through dense

environments in an in vivo model. The authors take advantage of the excellent intravital imaging conditions in Zebrafish embryos and the natural variation in confinement along the neural crest antero-posterior axis, and the authors make clever use of genetic manipulation and laser ablation to further modulate the degree of confinement encountered by the neural crest cells. Using this model system, the authors convincingly demonstrate that whereas cells in the cranial neural crest show little nuclear deformation as they migrate in a largely unconfined environment, cells in the trunk neural crest encounter a more confined environment due to the presence of somite cells and consequently exhibit transient large nuclear deformations. The authors further report that despite the large nuclear deformations, the migrating neural crest cells do not encounter extensive nuclear envelope rupture or increased DNA damage.

The analysis of the spatial variation in confinement and the nuclear deformation during the migration is convincing and provides further support for the idea that migration through tight interstitial spaces results in large nuclear deformation. Other findings, however, including the reported increase in nuclear pore size, decreased DNA damage, and changes in nuclear envelope composition, appear more preliminary and will require additional experiments and analyses to fully support the authors' conclusions.

The authors **<u>must address</u>** the following comments:

1) The authors report only a very small (~5%) change in the nuclear and cytoplasmic intensity of the nls-emGFP reporter and conclude that this change is not due to nuclear envelope rupture but arises from increased expression of the nls-emGFP over time and the leakage of the reporter through more permeable, stretched nuclear pores. This reviewer does not doubt the absence of extensive nuclear envelope rupture, and Stephen Young and Loren Fong's group had already described previously that migrating neurons in the mouse brain only exhibit nuclear envelope rupture upon deletion of lamin B1 or B2 (Chen et al. PNAS 2019, PMID 31796586). However, the nls-emGFP reporter and the BAF-mCherry reporters used here are not sufficiently characterized or **lack relevant positive controls** to fully interpret the findings and demonstrate that they have sufficient sensitivity to detect nuclear envelope ruptures. The authors show cytoplasmic localization of the nls-emGFP in mitotic cells, but in this case the nuclear envelope breaks down completely and chromatin becomes condensed, which is very different than the scenario of interphase nuclear envelope rupture, where holes in the nuclear membranes are only a few 100nm in diameter and chromatin is not condensed, which could affect interactions of the reporter with chromatin. The authors should demonstrate that their nls-emGFP reporter can reliably detect interphase nuclear envelope rupture, e.g., using laser ablation to damage the nuclear envelope or compressing cells in vitro.

We thank the reviewer for this comment. We have now provide positive controls (laser ablation of the nuclear envelope *in vivo*) for both nls-emGFP and BAF-mCherry (Figure S4B, lines 251-253; 275-77). In addition, we have further investigated NE ruptures using an additional reporter, cGAS-EGFP, expressed under control of a tissue-specific promoter and provide relevant laser ablation controls for this too (Figure S4E,F, lines 279-81). In summary, none of the 3 reporters detected evidence of nuclear envelope ruptures in in vivo migrating neural crest cells.

Furthermore, since fluorescence intensity measurements are sensitive to image settings, photobleaching, and changes in protein expressions, the authors should consider plotting the ratio of nuclear to cytoplasmic nls-emGFP, thus making it independent of the absolute intensity levels.

We thank the reviewer for this comment. We explored different ways to detect nlsGFP nuclear to cytoplasmic leakage in the NC cells. Here we provide cytoplasm to nucleus ratio for migratory and premigratory tNC cells as well as CNC migratory NC cells upon nuclear deformation, and we can similarly detect changes in this ratio upon deformation (Figure R3A). However, we can more sensitively detect changes in nuclear nls-GFP intensity than in the cytoplasm (Figure R3B). For this reason, we decided to quantify changes in *nuclear* nls-GFP throughout the manuscript. Of note, this intensity measurement is already carefully normalised and independent of absolute intensity levels (see Methods, *'Image analysis'* and PMID: 27013426).

Furthermore, to better compare the nuclear nlsGFP in different conditions throughout the paper, we have now also analysed the difference in nuclear nlsGFP upon maximum nuclear deformation (form -6min to 0min from max deformation) (Figure 4G, Figure S6J,L).

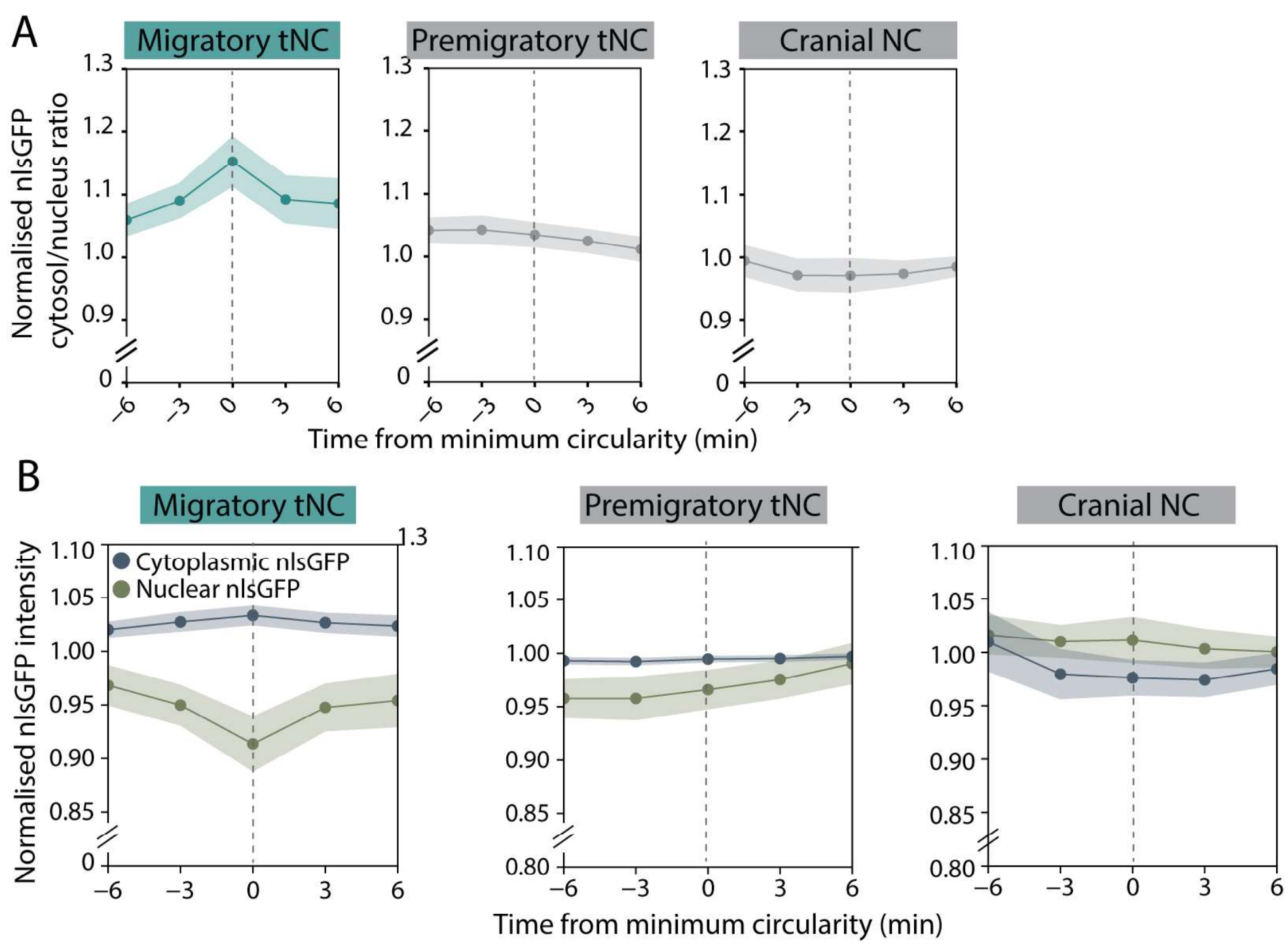

Figure R3.
A) Cytoplasm to nucleus ratio in migratory and premigratory tNC and CNC migratory NC cells upon nuclear deformation. B) Normalised cytoplasmic and nuclear nlsGFP signal in migratory and premigratory tNC and CNC migratory NC cells upon nuclear deformation. Midtrunk migratory n = n=50 cells from 9 embryos, premigratory n=17 cells from 4 embryos, CNC n=15 cells from 5 embryos. Mean and SEM presented. Y axis cut for better visualisation.

2) The authors attribute the changes in nuclear nls-emGFP intensity to stretch induced opening of nuclear pores, citing data from Suppl. Figure 4F-G as support. However, the reported difference in nuclear pore width is very small, less than 5 nm (Suppl. Fig. 4G), and appears well below what can be reliable measured based on the representative EM images in panel F", where the pore channel boundaries are not well delineated. Furthermore, the statistical significance appears to result only from the effect of pooling a large number of pores, all from a single embryo, which does not account for variation from experiment to experiment, sample prep, or embryo-to-embryo variability. Furthermore, the authors do not provide any functional evidence demonstrating increased nuclear transport through the pores under the confined conditions. The authors should either revise the text and soften their conclusions, or provide more thorough experimental data and more robust statistical analysis to support their interpretations.

We have now updated the quantifications to include data from 3 independent embryos. We still detect a small, but consistent increase in nuclear pore size.
We discussed with the EM specialists and -whilst TEM microscopy would have provided higher resolution, allowing more accurate measurements of nuclear pore sizes, a main limitation would have been the identification of *bona fide* neural crest cells in a highly magnified sample where spatial tissue organisation is hard to interpret.
Because of these technical limitations, we have toned down our conclusions in the text (lines 287, 289) and added a sentence about nuclear transport (line 495-6) in Discussion.

3) There are similar concerns regarding the analysis of the DNA damage. Although the data presented in the manuscript suggest that the confined migration does not lead to increased damage, the interpretation that DNA damage DECREASES is less well supported. The positive control for the 53BP1 marker is based only on a single representative image (Suppl. Fig. S5A) and lacks quantification.

We now provide the relevant quantifications for the validation of the mApple-53BP1 marker (Figure S5A,B).

Furthermore, the differences in DNA damage reported for the migratory cells based on gH2AX staining (Fig. 5B) and 53BP1 imaging (Fig. 5F') are quite small and might only be statistically significant by pooling a large number of cells, which does not account for experiment-to-experiment or embryo-to-embryo variability.
It is also possible that migration, regardless of confinement, alters the rate of DNA damage. To address this possibility, the authors should also quantify DNA damage in premigratory and migratory cranial neural crest cells.

We now provide analysis with two DNA damage reporters, γH2AX and 53BP1, for all NC regions, premigratory and migratory (CNC, aTNC, midTNC, postTNC) in Figure 5. We now plot both individual datapoints overlaid with single embryo averages for gH2AX to reduce the weight of a relatively large number of datapoints and to take into account embryo to embryo variability. Statistics reveals that DNA damage is significantly reduced only in the posterior tNC dataset (Figure 5A,B). We have also investigated 53BP1 dynamics in detail across all regions (Figure

5D). These results show that across the NC populations DNA damage does not increase in migratory cells, so we toned down conclusions accordingly in the text (lines 375-6).

The authors hypothesize that the lack of nuclear envelope rupture and DNA damage can be attributed to the reduced Lamin B2 expression and altered distribution of LAP2beta. While elucidating the complete mechanism would be beyond the scope of this manuscript, the current findings appear quite preliminary and insufficient to support the authors' interpretation. First, the authors should provide a more detailed quantification of the expression levels lamin A/C, lamin B1, and lamin B2, instead of only presenting quantitative data for lamin B2 (Fig. 6B).

We thank the reviewer for this comment. We have now provide quantitation of LaminA/C, LaminB1 and LaminB2 in the trunk embryonic tissues in Figure S6A-D and comment in the text on lines 386-387. Our quantifications indicate that LaminB2 is expressed at comparatively higher levels in neural crest cells than other Lamins, therefore we focus on LaminB2 for further functional analysis.

These data should also include cranial neural crest cells to show whether they also exhibit changes in lamin B2 levels, despite the less confined environment.

We now provide LaminB2 quantifications for all NC regions, including CNC (Figure 6A,B and comment in the text on lines 388-390). We find that LaminB2 level significantly decrease in migratory cells in more confined midtrunk and posterior in but not in cranial neural crest or in anterior trunk NC.

Since immunofluorescence labeling of lamins is sensitive to epitope masking or conformational changes (PMID 26301768 and 36809122), the authors should confirm the finding of reduced lamin B2 labels using orthogonal approaches.

These references show that *LaminA/C* epitopes are sensitive to conformational changes, and in fact one of the references uses the same LaminB1 antibody we use in this manuscript as a control; epitope masking has not been reported for LaminB1 or B2 to our knowledge. We attempted to validate our LaminB2 results (LN-43 mouse monoclonal, Abcam, previously validated in Zebrafish) using another antibody (X223 mouse monoclonal, Santa Cruz) targeted against the full length LaminB2, however, unfortunately this antibody did not cross react with Zebrafish (Figure R4).

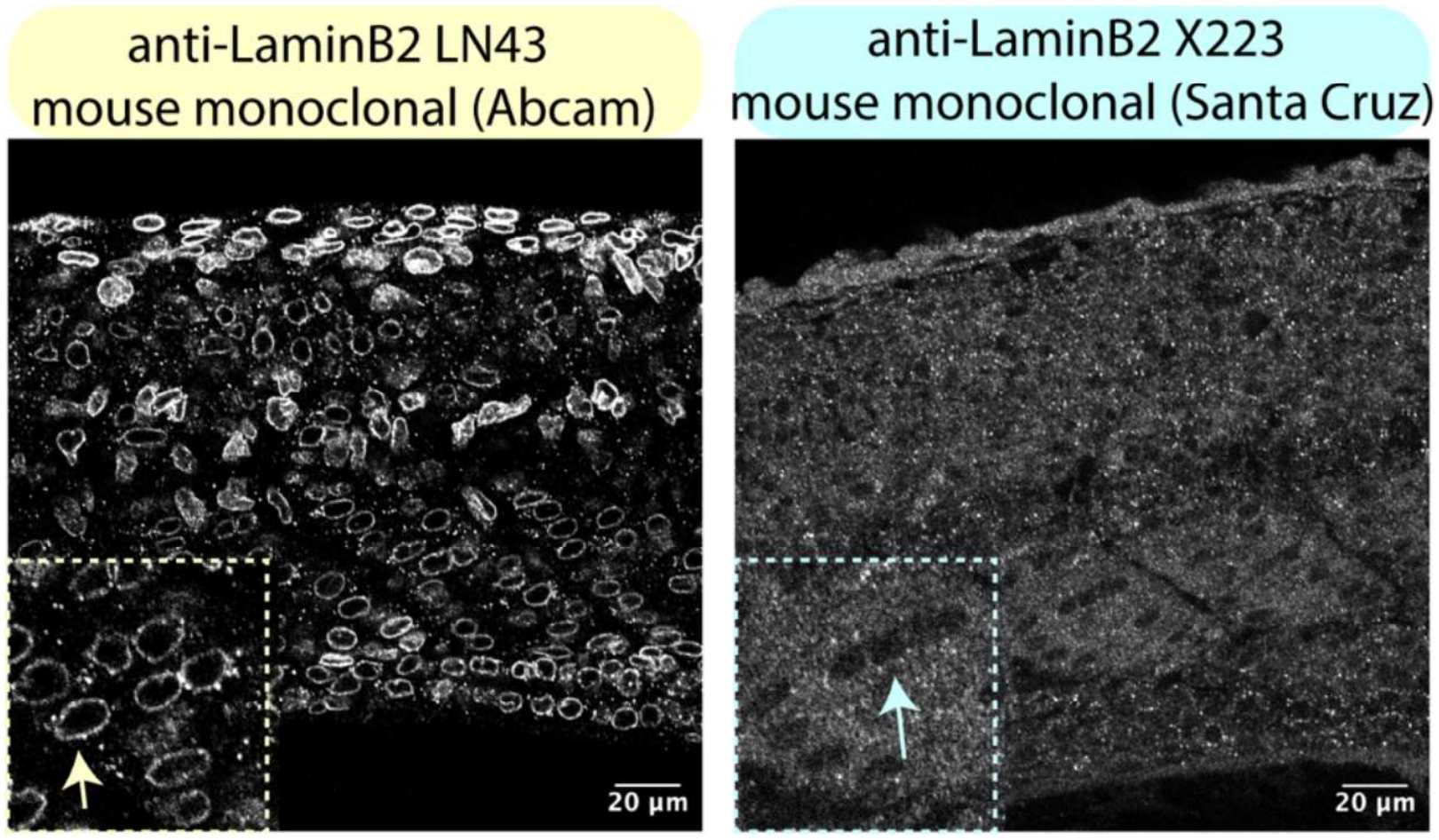

Figure R4. *Immunostaining of LaminB2 in Zebrafish trunk at 24 hpf using either the Abcam LN43 or the Santa Cruz X223 monoclonal antibody. Insets: zoomed in images, (arrow,left): LaminB2 immunoreactivity at NE was detected with LN43 but not with X223 (arrow, right).*

We discussed with the scientific team at Abcam, however, they did not disclose to us the exact epitope the LN43 antibody is raised against. As we cannot rule out that we are detecting epitope masking, we now mention this possibility in the main text, Discussion section (lines 508-510).

Furthermore, given that previous studies examining neuronal migration in the mouse brain found that deletion of lamin B1 and B2 increased, rather than decreased, nuclear envelope rupture, the authors should provide further support for their hypothesis, e.g., by testing whether overexpressing or depleting lamin B2 increases **nuclear envelope rupture** in the trunk neural crest cells.

We thank the reviewer for this comment. Studies in the mouse brain (PMID: 20145110) have shown that loss of Lamin B2 results in perinatal death defects in neuronal migration and in a mild nuclear envelope rupture and repair defect (PMID: 31796586). On the other hand, a recent study (PMID: 41706894) in melanoma reports that knockdown of LaminB2 abrogates NE permeability and NE ruptures under confinement. Our findings highlight a *physiological* downregulation of LaminB2 immunoreactivity in migratory trunk cells (Figure 6A-B).
We now provide extensive functional analysis of LaminB2 knockdown (Figure 6C-F, S6G,I,J) and tissue-specific overexpression (Figure 6G-J, S6H,K-L). We carried out Lamin B2 knockdown using a translation blocking morpholino oligonucleotide. Quantification of Lamin B2 levels in control and MO embryos showed efficient knockdown (77%, Figure S6E,F). We found that LaminB2 knockdown does not significantly affect nuclear circularity of mid-tNC (Figure 6D, E). However, it leads to shorter-lived nuclear deformation events (Figure 6F). Injection of LaminB2 MO in sox10KalTA4:nls-GFP embryos reveals that neither nls-emGFP nucleocytoplasmic leakage nor DNA damage are affected by LaminB2 knockdown in neural crest cells (Figure S6I,J). Thus, LaminB2 regulation in migrating tNC might support efficient nucleus deformation and recovery cycles.

To test whether increasing LaminB2 expression might affect the ability of neural crest to deform under confinement, we developed a tissue-specific, inducible approach for overexpression of LaminB2 (Figure 6G). We generated a new UAS-HaloTag-LaminB2 transgenic fish for

conditional expression of Halo-LaminB2 under control of a splitKaltA4 (PMID: 26485616) or of an inducible sox10-Kalt4 PMID: 32939715) and expressed HaloLaminB2 in mid tNCs. HaloTag-LaminB2 localised at the nuclear envelope and induced remarkable deformations of migratory tNC nuclei (Figure 6H,I). Quantification of nucleus circularity over time (Figure 6I) highlighted that nuclei remained significantly deformed in HaloLaminB2^{+} compared to HaloLaminB2^{-} control tNC cells leading to long-lived deformations (Figure 6J). Quantification of nls-emGFP nucleus intensity revealed that sustained expression of HaloLaminB2 did not affect nucleus permeability (Figure S6K,L) compared to HaloLaminB2^{-} control cells.

Sustained nuclear deformations can induce DNA damage in confined cancer cells (PMID 33326770). Thus, we investigated whether sustained expression of HaloLaminB2 in confined tNC could result in DNA damage using both γH2AX immunostaining and live imaging. For this, we generated a novel neural crest specific sox10: emGFP-53BP1^{trunc} transgenic reporter line (emGFP-53BP1). Similarly to the mApple-53BP1^{trunc} mRNA reporter, we validated this reporter line using Olaparib (Figure S7E) and investigated 53BP1 dynamics in triple positive sox10Kalt4, sox10: emGFP-53BP1^{trunc}, UAS-HaloLaminB2 embryos. We found that 53BP1 mean peak intensity in Halo-LaminB2+ versus Halo-LaminB2- control cells was slightly, but significantly increased (Fig 7A,B, yellow arrows). However, time-resolved analysis of 53BP1 peak intensity before and after maximum nuclear deformation did not show an increase (Fig 7C). Furthermore, we did not detect a correlation between minimum nuclear circularity and 53BP1 normalised peak intensity for up to 60 minutes after deformation (Fig 7D). Our results suggest that although persistent expression of Halo-LaminB2 in migratory tNC results in increase of 53BP1 peak intensity, the small difference to the control cells suggest that in addition to LaminB2 regulation, additional mechanisms might contribute to dampening DNA damage in migrating tNCs.

Lastly, the authors mention that lamin A/C is only found in some somite cells, but the figure referenced to support this statement (Fig. S6A) does not include any specific label to identify the cells with high lamin A/C levels as somite cells.

We now more clearly highlight these cells in Figure S6A. Unfortunately, both somitic markers antibodies commonly used in Zebrafish (DSHB F59: slow muscle cell marker, DSHB F310: fast muscle cell marker PMID: 16162652) are mouse monoclonal and incompatible with our LaminA and LaminB2 Zebrafish validated mouse monoclonals. We now quantified nuclear envelope enrichment of LaminA/C across all trunk tissue and specifically in this somite subpopulation as a positive control for the LaminA/C antibody (Figure S6B).

5) Related to the above comment, the authors' sole focus on LAP2beta is not sufficiently justified, and the authors should examine additional nuclear envelope proteins to determine whether the observed redistribution is specific to LAP2beta or reflects broader changes in nuclear envelope organization. The authors should particularly consider other nuclear envelope proteins that have been implicated in modulating nuclear envelope rupture, such as some of the candidates identified in a recent high-content screen by the group of Emily Hatch (PMID 38472343).

We decided to investigate Lap2beta because another study had previously highlighted a depletion of Lap2beta at the nucleus front during *in vitro* confined migration (PMID2 7013426).

Critically, Lap2beta links the INE with peripheral chromatin and it is essential for preserving genome homeostasis (PMID: 41735607). However, a recent report investigating NE ruptures in melanoma excluded a role for Lap2beta in NE ruptures (PMID 41706894), hence we focussed our functional investigations on LaminB2.

We now provide a detailed quantification of LaminA/C, Lamin B1 and Lamin B2 across the Zebrafish embryonic tissues (Figure S6A-D). Investigating NE proteins in Zebrafish is a challenge due to limited availability of cross-reacting antibodies. Upon consideration of a recently published report highlighting a role for a LaminB2 interactor, Lamin B Receptor, in nuclear envelope fragility (PMID 41706894), we also attempted investigation of endogenous Lamin B Receptor localisation as the antibody epitope was 100% identical between human and Zebrafish, however the antibody staining provided inconsistent, unreproducible results.

We thank the reviewer for the helpful suggestion of the Hatch Lab screen. This is indeed a valuable resource to us and to the community for future studies. For the revised version of the manuscript we have generated an RNA seq dataset of premigratory (non confined) versus migratory (confined) tNC (Figure 8A-F, S8C). We investigated the expression of candidate genes from the Hatch lab in these datasets and we cross-checked these with publicly available Zebrafish sc-RNAseq datasets (PMID: 39454574). However, we found that only a handful of these candidates are expressed in the migratory Zebrafish neural crest at relevant developmental stages, and only 2 (sun1b, nrm) are upregulated in migratory cells:

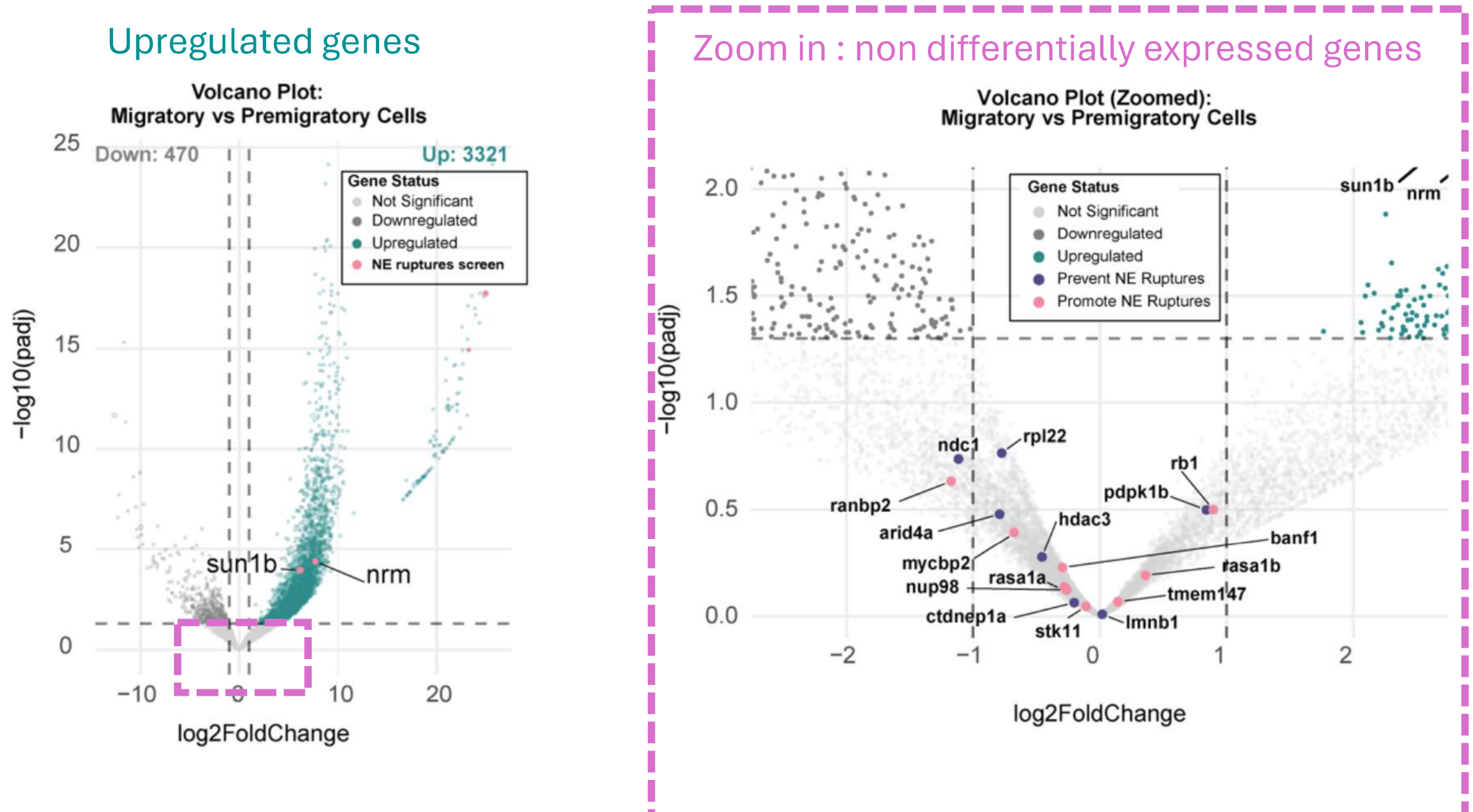

Figure R5. A) Volcano plot of significantly upregulated versus significantly downregulated genes in Migratory tNCs. The two significantly upregulated genes (sun1b, nrm) from the Gunn et al. screen are highlighted. B) Zoom in of the volcano plot on non significantly different genes, genes found to regulate NE rupture in the Gunn et al. screen are highlighted.

Of these, we were already investigating *sun1b* for a separate project in the lab, and we found that Sun1b is only detectable by antibody staining in cranial, but not trunk neural crest, and that indeed Sun1b knockdown by using a previously published MO does not affect trunk neural crest migration or nuclear deformation dynamics (S. Villaseca, unpublished data). For nrm, the

Zebrafish ortholog has not been yet characterised (https://zfin.org/ZDB-GENE-060503-445#summary), investigating this would require significant time and resource investment beyond the scope of this paper.

6) The authors should explore other mechanisms that might protect the neural crest cells from DNA damage and NE rupture, such as the role of BMP and WNT signaling, as alluded to in the discussion.

Two recent papers identified a link between BMP signalling and replication-stress induced DNA damage during Zebrafish heart regeneration (PMID: 39962064) and in Zebrafish primordial germ cells (PMID: 41839895). BMP also acts upstream of Wnt in trunk neural crest (PMID: 15456730). We inhibited BMP with 5μM of a BMP inhibitor previously validated in Zebrafish (PMID: 41839895) for 6 hours prior to imaging (from 12 hpf to 18hpf) in emGFP-53BP1trunc line to directly assess DNA damage response. We found that mid tNC were able to migrate under BMP inhibition and we could not detect changes in 53BP1 in migratory population, although 53BP1 was slightly increased in premigratory population (Figure S8A,B).

We also addressed whether a Zebrafish validated ATR inhibitor (PMID: 39962064) affects the mid tNC migration and leads to DNA damage response incubating emGFP-53BP1trunc embryos in 40 uM VE821fr 6 hours prior to imaging. ATR inhibition did not lead to DNA damage response in the premigratory nor migratory NC populations (Figure R7, see *Response to Reviewer 3* section).

7) It appears that the trunk neural crest cells already exhibit more irregularly shaped nuclei in the premigration state (Fig 1C,D). The authors should comment on this and provide potential explanations, including whether this suggests differences in the physical environment or in the cells themselves.

Premigratory neural crest are moderately confined in height, as they lie a flat space between the embryonic epidermis and the neural tube (see cross section view in Figure 1B, cyan arrow and SEM cross section (Figure 4H).
This has now been clarified in the main text (lines 118-19) and a comment on stronger deformation of posterior premigratory cells has been added (line 186).

8) In the description of the extracellular environment encountered by the trunk neural crest cells, the authors focus only on the role of collagen, particularly Col1a1a (Fig. 1C, D), while also mentioning the presence of hyaluronic acid (Fig. S2B, Fig. 2C). The authors should also comment on the presence of other extracellular matrix molecules that could contribute to the confinement, such as laminin, fibronectin, etc.

Networks of fibrillar collagen, but not laminin or fibronectin, have been previously shown to confine nuclei of migrating cells (PMID 23798731). We already comment on Fibronectin and Laminin organisation in the Zebrafish trunk on lines 155-156:
"trunk neural crest migrate confined in between closely apposed surrounding tissues (Figure 2B), which are lined with fibronectin and laminin"

9) In Figure 2D and 2F, as well as the corresponding explanations in the method, figure legends, and results sections, the authors present inter-tissue spaces as a linear distances (i.e., measured in µm). While this is a useful metric, the authors should also provide information on the other dimensions, or the cross-sectional area of the interstitial spaces, which might be a better predictor of cell behavior than a one-dimensional measurement

We indeed analysed the space between the neural tube and somite by measuring the cross-sectional width of the interstitial spaces between these adjacent tissues (see Figure 2E-F, S2C-F). For maximal resolution the measurements were done from transverse optical sections of a dorsally oriented embryos moving from dorsal toward the ventral along Z every for every 2.5µm. We used the line tool in Fiji spanning across the mediolateral axis of the embryo and created a plot profile of the intensity, and measure the peak of the profile for maximal accuracy. Please see lines 454-463 and an additional Supplementary Methods Figure 1 in methods section.

10) For Figure 5B, the authors applied a Kruskal-Wallis test to test the comparison of the mean gamma-H2AX intensity between the premigratory and migratory neural crest cells. As the comparison is between two groups, the authors should use another more appropriate statistical test, such as a Mann-Whitney U test.

Thank you for spotting this. We had carried out a Mann-Whitney test but reported it incorrectly, this has been now rectified.

Minor comments

11) The authors should better acknowledge previous in vivo studies that demonstrated increased nuclear deformation, nuclear envelope rupture, and DNA damage. In addition to the Denais et al. and Raab et al. Science 2016 publications, which are cited only for their in vitro findings, the authors should include work by Abby Dernburg's group on nuclear deformation and nuclear rupture during oogenesis in C elegans (PMID 37436986), work by Stephen Young and Loren Fong in mouse neuronal migration (PMID 34161290 and 31796586),

Thank you for the suggestions. We now refer to the Young and Fong lab's work in lines 391-392 and 525-26 and we now cite the Penfield et al 2018 paper from the Bahmanyar lab (PMID: 29386297) which directly addressed NE ruptures in a LaminB hypomorphic mutants in C Elegans oogenesis.

and live imaging in human blastocysts by Nicolas Plachta's group (PMID 37413989).

We focussed the introduction on examples of in vivo nuclear deformation in the context of developmental or physiological cell migration, so, although this work is fantastic and very valuable, we did not include it in the interest of space.

12) Page 5, Line 181. It appears that the beginning of the sentence is missing some works, which have been accidentally replaced by “ts”. Please correct the sentence.
This has now been rectified, thank you for noticing.

13) Page 5, line 185. The authors should comment on whether the spt mutant also affects neural crest cells, or only somite cells.

Revised manuscript Line 198-99: We now clarify that neural crest formation is not affected in spt mutants but that migration is unrestricted due to lack of somites (PMID 8565850, 16162652).

14) Page 5, line 193. The authors use the acronym EVL without defining it. Please revise the sentence to provide a definition.
This has now been clarified in the main text (EVL is defined at line 119).

Reviewer #3 (Remarks to the Author):

The manuscript studied the adaptive migration of trunk neural crest cells in zebrafish embryo as they navigate through narrow passageways between the neural tube and somite. The authors observed dynamic and significant nuclei deformation during this process, which is responsive to tissue confinement. Such deformation led to stretches of the nuclear envelope, expansion of the nuclear pores, but not rapture of the nuclear envelope or DNA damage. The protection from DNA damage likely results from relaxed nuclear envelope by decreased LaminB2 levels and decreased chromatin stress by depletion of Lap2beta from the leading edge of the nuclei.

While the concept of adaptive migration under confinement and nuclear deformation is not new, detailed analysis of this process in vivo is novel. This study provides insights into how neural crest cells adapt to tissue confinement without increasing DNA damage, in contrast to rupture of nuclear envelope in T-cell migration in highly confined in vitro conditions and increased genome instability during cancer cell metastasis.

The zebrafish trunk neural crest cell is a good model to study adaptive migration in vivo. The design of the study is logical and appropriate, using validated approaches and necessary controls are included. The quality of data is good in general, and the results are described clearly. Statistical analyses were performed when needed. Sufficient references are included. The conclusions are reliable.
There are some errors and minor issues noticed.

Figure 1, the definition of “premigratory zone” is unclear. It is more of NCCs before entering the narrow space underneath the somite rather than before NCCs initiate migration.

Please also see response to reviewer 2, point 7:

This has now been clarified in the main text (lines 118-19) and a comment on stronger deformation of posterior premigratory cells has been added (line 186).

Figure 1 and S2, is the nuclear diameter the length of the short axis? The size of nuclei appears less than 10um (scale bar) in Fig.1A but measured as >= 10um in FigS2D.

The measurement in Figure S2D is defined as the mean nuclear axis diameter. This metric corresponds to the mean value of the nucleus's short and long axis.

Figure 3, in spt mutant, while the inter-tissue space is significantly wider, the effect on nuclei circularity or aspect ratio was not obvious. Here, pairwise comparison was performed. Is it because the sample size is smaller? Also, are the measurements done at mid-trunk level or posterior-trunk level? This is not indicated.

Please see response to Reviewer 1, point 8:

We have now better clarified in the text that we investigate the spadetail mutant in the mid-trunk region (line 198-199), where somites are affected (PMID: 2911386, also cited in main text).

Figure 3E,J: we used linked individual data points here as we specifically address changes in nuclear shape for cells that transition from the premigratory region to under somite confinement in control versus perturbed somite conditions.

Figure 3H, in the ablated region, despite wide opening space, there does not seem to be increased number of NCCs migrating into the region. A discussion of whether tissue confinement influences cell entry or migration speed will be helpful.

Previous studies have shown that a subset of somitic cells (slow muscle cells) are required for guidance of trunk neural crest in Zebrafish (PMID 16162652, PMID: 37390228). Slow muscle derives from the medial-most part of the presomitic mesoderm (adaxial cells). During ablation experiments, we carefully avoided targeting adaxial cells. However, since somite morphogenesis is a complex process driven by interactions between different somite cell subpopulation (PMID PMID: 30253169), it is possible that ablations affect slow muscle specification, thus reducing guidance cues for migrating neural crest. However, the aim of this experiment was to reduce confinement, rather than interrogating mechanisms of neural crest guidance. We now add a comment in the main text, line 231.

Figure 4C', D' and Figure S4C', the use of different scales for nuclear and cytosolic GFP intensity is unnecessary and confusing.

This has now been rectified in Figure 4D'-F'. The change of nlsGFP nuclear intensity before and after deformation is also directly compared in Figure 4G.

Figure 5A-B. It is surprising that in addition to not having an increased DNA damage, NCCs

under confined migration show decreased DNA damage. The manuscript later discussed potential mechanisms to reduce stress, but why DNA damage is even less than cells without confinement stress?

Please see response to Reviewer2, point 3 for an update on DNA damage quantifications and statistics. Our updated quantifications across all NC subpopulations (Figure 5A-D) highlight that DNA damage in fixed samples only significantly decreases in the posterior tNC. Live quantifications of DNA damage dynamics do not highlight a significant decrease, however we are now very confident that DNA damage does not increase even in strongly deformed cells in tNC (Figure 5E,F).

In the revised manuscript we test several hypotheses to explain why this happens :

1) We manipulate the levels of LaminB2 to test whether the physiological regulation of LaminB2 immunoreactivity we observe contributes to protect tNCs from DNA damage. We found that whilst knockdown of LaminB2 does not affect DNA damage (Figure 6A,B), tissue-specific sustained expression of LaminB2 induces nucleus shape changes and results in a mild, but significant, increase in DNA damage (Figure 7A,B).
2) However, since overall DNA damage levels in migratory tNC remained low, we reasoned this might originate from the nature of the tissue confinement, as in vivo embryonic tissues might be soft. See Response to reviewer 1, point 5 " *For this reason, carried out atomic force microscopy of thick, live Zebrafish mid-trunk cross-sections and measured tissue stiffness (Supplementary Figure S5H-J). These findings highlight that, in line with previous AFM measurements of Zebrafish trunk tissues (PMID: 35165417) tissues appear indeed soft (~0.4-0.5 kPa) compared to the PDMS material often used in microfabrication studies (PDMS stiffness ~1.3MPa PMID: 17653351). To test whether neural crest cells might experience DNA damage when confined within rigid, non deformable boundary conditions, we adapted a protocol for primary Zebrafish neural crest culture (PMID: 24326414) to trunk neural crest cells. We confined primary Zebrafish TNC in PDMS pillar forests with pillars spaced at a 3μm distance between each other and assessed DNA damage using the mApple 53BP1 live reporter (Figure 5J-L). Nuclear shapes in these devices appear strongly deformed, however quantifications of 53BP1 dynamics reveal that neural crest cells still do not incur DNA damage under stiff in vitro confinement".*
3) See response to reviewer 2, point 6. We tested whether BMP or ATR inhibition might be involved in protecting migrating tNC from DNA damage, but we found this not to be the case (see Figure R7 below).
4) To further investigate how migrating tNC may prevent DNA damage when migrating in confinement, we have now carried out low-input bulk RNA sequencing (Figure 8A-E, Supplementary Figure 8C). Using a neural crest specific photoconvertible transgenic line (Sox10:H2B-Dendra, PMID 32939715), we photoconverted trunk neural crest before confined migration (16hpf) or during confined migration (23-24hpf) and carried out bulk RNA-seq for a small sample of cells (n=20 cells per sample, 4 replicates per condition). PCA analysis revealed that confinement significantly alters gene expression of tNCs. Strikingly, the most significantly upregulated biological process in confined tNC is the DNA damage response (70 genes). Careful analysis of this gene group reveals that components of several pathways involved in repair of DNA-ds breaks are significantly upregulated in confined migrating tNCs, thus indicating that this stem cell population likely is endowed

with a complex programme allowing them to cross unscathed through confining tissue environments.

Is cell-cycle delay sufficient for lower DNA damage?

A previous study in Zebrafish trunk neural crest indicated modulation of cell cycle in leader cells versus follower cells with leaders predominantly initiating migration in S/G2 phase of the cell cycle and followers predominantly migrating whilst in G1 (PMID 35438077). Therefore, as this might indirectly indicate whether DNA damage might occur in a specific phase of the cell cycle we quantified γH2AX in leaders versus follower migratory cells. However, we did not detect a significant difference in γH2AX between these two subpopulations of migrating cells (Figure R6).

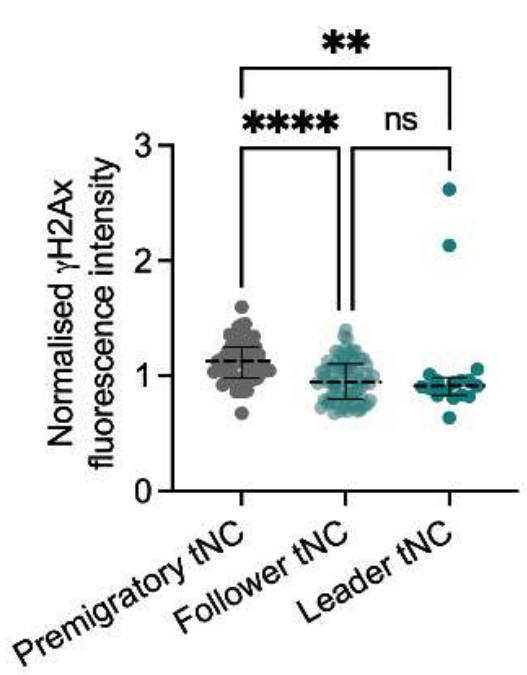

*Figure R6: Normalised γH2AX intensity in leader versus follower tNC subpopulations (n=57 cells Premigratory, n=69 cells Followers, n=17cells Leaders).**p<0.01, ****p<0.0001 ns:not significant, Kruskal Wallis test.*

Sustained nuclear deformations can induce DNA damage due to replication fork stalling in confined cancer cells (PMID 33326770). ATR is the main signalling kinase involved in resolution of replication stress (PMID 24366029). We carried out inhibition of ATR using a highly specific inhibitor previously validated in Zebrafish (PMID 39962064) but this also did not result in increased DNA damage as detected by 53BP1 live imaging or γH2AX immunostaining, thus suggesting that tNC do not experience nucleus deformation-dependent replication stress (Figure R7).

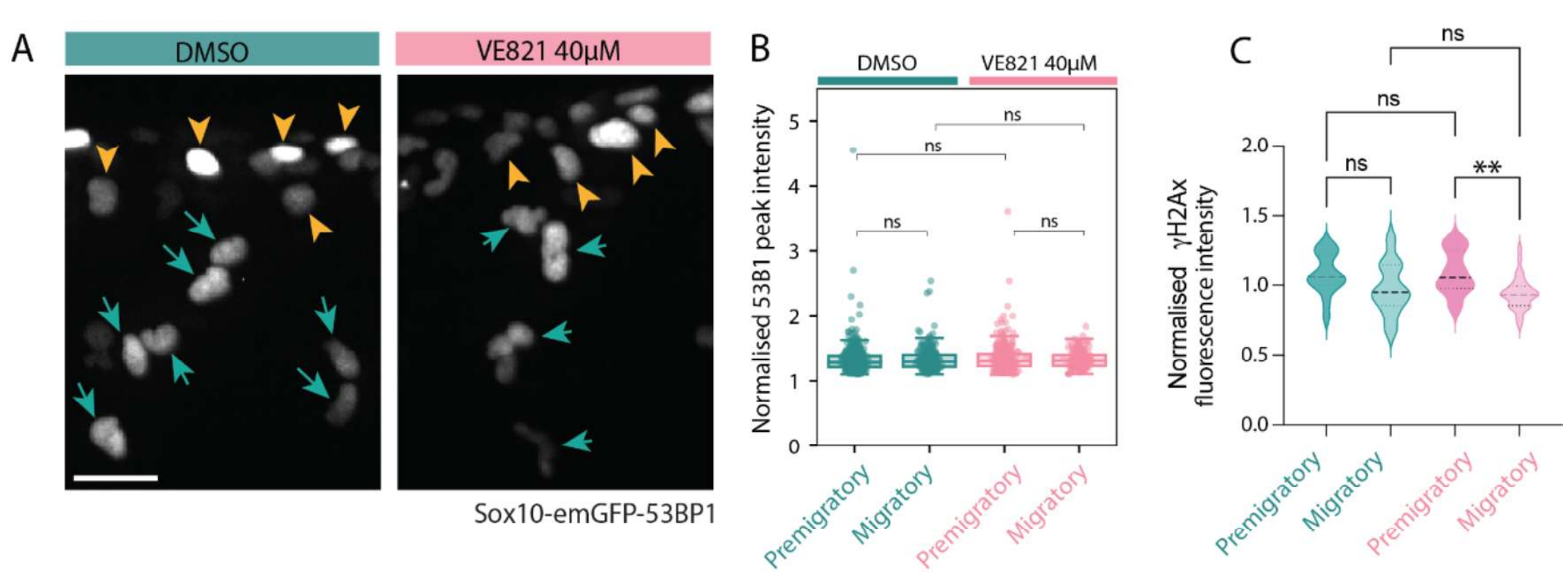

Figure R7.
A) Representative image of midtrunk NC steams in Sox10-emGFP-53BP1 embryos in DMSO and in ATR inhibitor (VE821, 40uM) after 6 hours incubation. Green arrow shows the migratory cells, Orange arrow heads shows the premigratory cells.
B) Quantification of normalised 53BP1 peak intensity in premigratory and migratory cells in DMSO (n= 514 premig cells, n=258 mig cells, VE821 n=351 premig cell, n=169 mig cells). Kruskal Wallis test. ns>0.3.
C) Normalised γH2AX intensity in DMSO versus VE-821 treated cells. (DMSO Premig n=16 cells Mig n=21cells ; VE-821 n=24 cells Premig n=34 cells Mig).
Scalebar 20μm.

Is this decrease in DNA damage also observed in migrating neural crest cells at cranial/vagal levels where there is no confinement or in embryos with ablated or mutated somites?

We have now investigated DNA damage using both γH2AX immunostaining and the mApple-53BP1 live reporter across neural crest regions (Figure 5A,B,D). We do not detect a significant change in DNA damage in less confined regions (CNC, anterior tNC).

Also, why premigratory and migrating NCCs have a significant level of phosphorylated gammaH2AX comparing to neighboring mesenchymal cells?

We normalise γH2AX fluorescence intensity to the NC immediate surroundings (see Supplementary Methods Figure 2). We use this method to minimise the impact of differences in antibody penetration through thick tissue, as the migratory cells are located deeper within the embryo than premigratory cells (see Figure 1B), and hence less accessible to antibodies. We find that premigratory, but not migrating tNC have slightly higher levels of DNA damage than surrounding tissues. However, γH2AX fluorescence intensity varies between tissues, and whilst premig tNC are mostly surrounded by neural tube cells, mig tNC lie at the interface between neural tube and somites, which have a higher γH2AX intensity (also see Fig 5B, arrowheads).

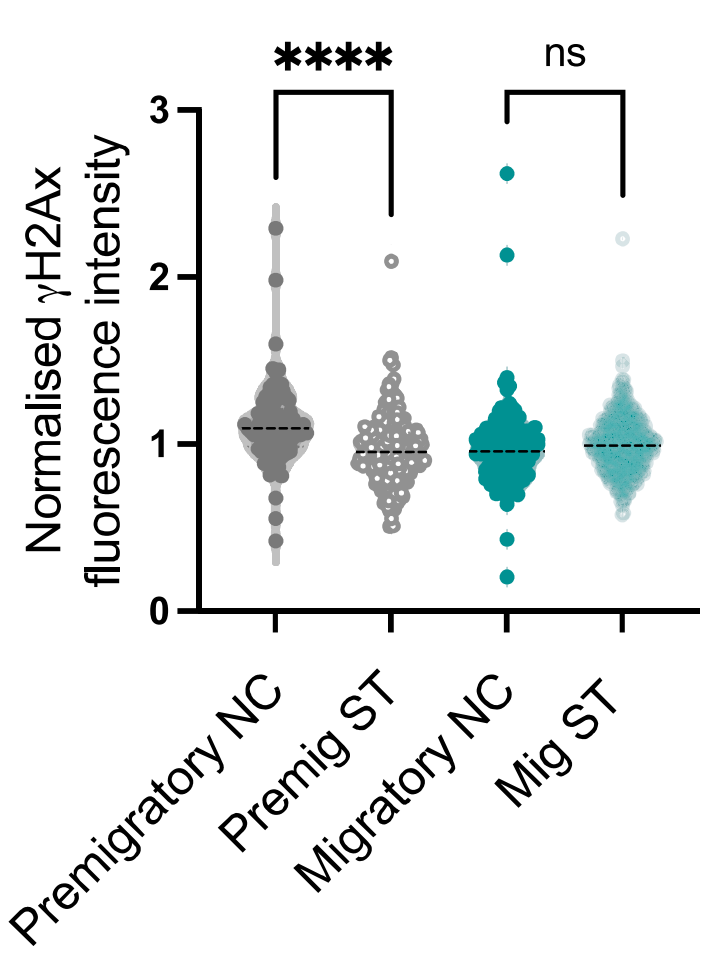

Figure R8: Normalised γH2AX intensity in mid-trunk tNC versus surrounding tissues (n=88 cells Premig tNC, n=272 cells Premig ST, n=117 Mig tNC, n=544 Mig tNC ST). ST = surrounding tissue.

In text, line 296, “compare pink arrows and arrowheads”, should arrowheads be yellow arrows?
This has now been rectified in the text.

Figure 5D, why compare nuclear circularity prior to 53BP1 foci appearance in premigratory and migratory crest cells? No difference here may mean that DNA damage appears at a certain pattern (deformation of nuclei). However, I think the point the authors want to make here is that the foci appear randomly. This should be demonstrated by a broad distribution of circularity index.

Please see Response to Reviewer 1, point 5.

Figure 6. The polarized distribution of Lap2beta was not very clear. It seems be a general decrease of signal levels in highly deformed/stretched nuclei. When there is blebbing of nuclei, due to the stretching of the nuclear envelope, it may naturally be a decrease in the concentration of proteins associated with the envelope. This is not distinguished from an active polarization event. In the figure legend for Fig6C, “white arrowheads” should be “white arrows”.

We agree with the reviewer that the decrease in Lap2beta at the nucleus front might be a consequence of membrane stretching rather than an active polarisation event. Indeed, we did not define this as an “active” polarization event in the first version of the manuscript (see lines 355-356 of first submitted manuscript). We have now further toned down the text (line 296 in reivsed manuscript: we changed “is dynamically depleted ” to “appears dynamically depleted”). Of note, depletion and recovery of Lap2beta from the nucleus front has been previously observed both in HeLa and mouse dendritic cells migrating in PDMS microchannels (PMID 27013426).

Line 50, “device to for cells”
Line 130, “Fig1C, D, arrows”, there are no arrows in Fig1C, D.
Line 181, the first sentence in the paragraph needs revising.
Figure S3C, the label was messed up.

All of these are now rectified in the text, thank you for pointing these to us.

Dear Daryl,

We are grateful for the time the reviewers dedicated to the revised version of our manuscript. We now provide a revised manuscript where we worked our best towards addressing the outstanding reviewers' comments.
Please find enclosed a point by point response below (our responses highlighted in green).

Reviewer #1 (Remarks to the Author):

This re-review of resubmitted manuscript NCB-A58386A shows that the authors have responded directly and conscientiously to all of my concerns and suggestions, with intriguing new information about the notable characteristics of neural crest cells with respect to DNA damage protection. The results help to advance the field conceptually, so I am now happy to support its publication with congratulations to the authors on their fine work.

Reviewer #2 (Remarks to the Author):

The authors have submitted a revised manuscript that includes additional experiments and several updates to the text that have substantially improved the manuscript. The authors are commended for the thorough revision and inclusion of additional data. The new data and revised text address many of the previous concerns and convincingly show the extend of nuclear deformation during trunk neurocrest cell migration in vivo and the lack of nuclear envelope rupture and DNA damage during this confined migration.

Nonetheless, some of the originally stated concerns remain, particularly regarding the statistical analysis, and the new RNA-seq data and experiments using lamin B2 depletion/overexpression raise some new questions.

The authors need to address the following concerns:

1) Many of the reported effects are extremely small and often appear to only reach statistical significance by pooling a large number of cells or nuclear pores, which is prone to results in statistically significant differences that are not biologically relevant. For example, the reported difference in the nuclear pore diameter (Fig. 4I), the differences in the dynamics of nuclear deformation (Fig. 6F), or the 53BP1 accumulation upon lamin B2 overexpression (Fig. 7B) have effect sizes that are well below the variability between individual measurements and possibly between individual embryos. Based on previous comments, the authors now show the mean values for individual embryos in some figure panels (e.g., Fig. 5B, Suppl. Fig. S7B, D), allowing for more robust interpretation, but this remains the exception. Most graphs still only show the individual data points, with the information about the number of embryos and independent experiments only listed in the figure legend, and it is unclear how this information was used in the statistical analysis. To increase the robustness of the findings, the authors should show superplots (i.e., individual data points plus the mean values for individual embryos) in all figures and perform statistical analyses that consider the embryo-to-embryo and experiment-to-experiment variability and then update the text and conclusions accordingly. I do believe that the key findings about nuclear deformability and the lack of nuclear envelope rupture and DNA damage are robust, but many of the other, more subtle findings appear much less robust, which can affect the interpretation of the findings.

We understand this reviewer concerns, and we take this as an opportunity to further clarify our statistics considerations and improve the data presentation.
Wherever observed effect sizes are small and/or statistical tests only show a $p<0.05$ we have been careful with our wording already in the earlier versions of the paper.
We have now carried out statistical tests for effect sizes as requested by the reviewer and reported Cohen's *d* in the relevant figure legends for panels Fig3J, Fig4I, Fig6F, Fig7B.

To further reassure the reviewer, we now provide "superplots" for all datasets represented in boxplots to show both cell to cell variation as well as embryo to embryo variation.

We also adjusted our statistical approach and applied the following criteria:

1) for fixed imaging datasets (Figure 4I, Fig5B, Fig6B, EDFig6B,C,D,F, EDFig7B, EDFig7D), as well as for live data with large number of embryos (EDFig7F, EDFig8B) where one datapoint indicates a single measurements within each nucleus/cell, we provide a superplot of individual embryos and carry out statistics on *per embryo* mean values (as already described in the figure legends).

2) For live imaging quantifications, we have now overlaid a superplot of individual embryos averages to improve data presentation and transparency. However, we think it is reasonable to carry out statistics comparing cells (Figures and ED Figures 1,3,4,5,6,7,8) or somites (Figure 2 and ED Figure 2: tissue space measurements) rather than embryos in the data panels, because datapoints already represent a mean value per cell/per somite rather than single measurements and we have already strongly reduced the dimensionality of the raw time resolved data:

For live imaging data sets (Fig1C, EDFig1G,H, EDFig2D, Fig5D,I,L, Fig6D,F,H,J, Fig7B) each data point in the relevant boxplots is indeed a mean value per cell/per somite calculated over a time series of individual measurements for the time period analysed. Over-time raw data curves are highly variable, as shown in the examples of nuclear shape metrics over time for an individual nucleus we provided in EDFig1E,E'. Similarly, data points in figure panels Fig2D,F, Fig3C represents a mean value of measured space for 5 different positions along the migratory path. Finally, nuclear nls-emGFP analysis shows time resolved changes in nlsGFP dynamics between consecutive time points in Fig4G, EDFig6J,L and each value is derived from more than one measurement per cell.

The information about what each datapoint represents and the statistical test used is already described in the figure legends. We have now further reviewed the figure legends to further clarify what each datapoint and each boxplot represents in every panel for both main figures and ED figures.

2) In the abstract and at the end of the introduction (lines 106-108), the authors highlight the role of lamin B2 as a key adaptive change in nuclear envelope composition to enable confined migration without experiencing DNA damage or nuclear envelope rupture. However, based on their new experiments using lamin B2 depletion or overexpression, it appears that the effect of changes in lamin B2 levels is much more subtle and primarily affects nuclear deformation without necessarily explaining the lack of nuclear envelope rupture or DNA damage. The authors should revise their text and soften their conclusions to reflect this.

We thank the reviewer for this comment. We have now revised abstract (lines 45-46), introduction (lines 99-100),results (lines 462, 467) and discussion (lines 552,554-55) to better clarify our findings and softened conclusions.

3) The new photoactivation/RNA-seq experiments are very laudable and provide new insights. However, their interpretation is not entirely clear and likely not as straight forward as the authors make it appear. DNA damage response pathways are typically upregulated in response to DNA damage, or to facilitate improved DNA repair, and not to prevent DNA damage in the first place.
Thus, an alternative interpretation might be that the cells experience subtle DNA damage insufficient for detection by fluorescence microscopy but sufficient to activate DNA damage response pathways. Another interpretation is that the reported changes represent developmental differences, given that the cells for the unconfined conditions were photoactivated and collected at a different time point (16 hpf) than the cells used for the confined migration conditions (23-24 hpf). A more direct comparison would be to photoactivate cells at the same time point, but using cells in different locations along the migration path.

We thank the reviewer for this comment. We further clarify our experimental design below:
We compared premigratory cells (16 hours post fertilisation, hpf) versus migratory cells at 23-24hpf (endpoint of migration), for two reasons:

a) We wanted to assess the same population of cells (neural crest cells migrating in the ventromedial pathway, between neural tube and somite) *before* and *after* confined migration. The experimental design suggested by the reviewer would compare cells in regions with differential confinement at the same timepoint. However, at 24 hpf a subset of non-migratory neural crest cells are left behind in the premigratory area. These cells will later differentiate into pigment (PMID 8162850), whilst ventromedial cells predominantly take neuron and glial fates (PMID 8162850; PMID 35756992). For this reason, we chose an experimental design aimed at minimising confounding factors linked to differential cell identity.

b) 23-24 hpf is the *endpoint* of migration, a process that takes several hours (8-10 hours at 28°C). This now better clarified in the text and in the figure 8 title too (lines 483, 490, 912).
Thus, we reasoned that our chosen experimental design would allow us to assess the cells' *response* to confined cell migration.

In addition, to more directly test whether the increased expression of various DNA damage response pathways is indeed protective of DNA damage, the authors *could* test whether the tNC cells at the later stage are protected against other types of DNA damage, e.g., UV or chemically induced DNA damage. While a detailed analysis of the precise protective mechanisms is beyond the scope of the manuscript, without additional data it is difficult to justify the current interpretation that the upregulation of the DNA damage response pathways, or the depletion of lamin B2, is solely the reason for the lack of DNA damage during the trunk neural crest cell migration.

We thank the reviewer for this comment, and we agree that these results raise new questions and interesting prospects. How DNA damage response pathways might be activated in confined migratory NC cells remains unclear and will be our interest to investigate this further in future projects in the lab.

Our bulk RNA sequencing data reveals genes involved in multiple DNA damage repair pathways (see Figure 8D, 8E) including HR, NHEJ and checkpoint signalling. Our current interpretation of these results is that migration of NC cells in confined tissue environments might cause mechanical stress for the nucleus and that a large range of DNA damage repair genes is activated for quick repair to avoid accumulation of deleterious DNA damage.

Further investigation is required to understand how cells might be sensing confinement to mount an upregulation of the DNA damage response. To investigate in detail the molecular and cellular mechanism behind the DNA damage response activation in future studies we may conduct further *in vivo* and in vitro assays and carry out RNA seq for cells experiencing different range of confinement. Furthermore, future pharmacological and genetic approaches to inhibit nuclear mechanosensing pathways and screening for the requirement of specific components of the upregulated DNA damage repair pathways could be used to pinpoint specific DNA repair factors involved.

We have now added a paragraph in the discussion section (lines 574-580) to better acknowledge the need for further investigations and include possible future prospectives.

However, our view is that these findings open a new avenue of investigation that is beyond the scope of the current manuscript.

4) Lines 249-250. The authors state that "cells in the ablated region showed a more subtle decrease in nuclear circularity", referring to Fig. 3J. However, the data in Figure panel 3J still show a significant change in the mean circularity upon migratory transition in the ablated region ($p < 0.05$), and apart from two cells in the adjacent somites, which show very large changes, the effect size seems to be quite similar. The authors do not include any statistical analysis comparing the effect size between these two conditions, which would be necessary to justify the statement of a "more subtle decrease", and the conclusions appears to be based solely on a less significant p-value ($p < 0.05$ vs. $p < 0.001$) between the conditions, which is not a measure of the effect size and could simply be due to the smaller number of cells analyzed in the ablation group. The authors should either perform appropriate statistical tests to demonstrate a difference in effect size or revise their text accordingly.

We now provide an effect size test for the figure 3J (see relevant figure legend, Cohen *d* is now reported). We clarified the text accordingly (lines 240-241).

Notably, circularity is not the only metric we used to assess nuclear shape changes. Aspect ratio metrics for the same dataset are provided in ED Figure 3F. These show:

1) a clear effect size difference in circularity between migratory cells in control and ablated embryos;

2) cells in control embryos show a marked increase in aspect ratio, whilst ablated embryos show a non-significant increase in aspect ratio (Wilcoxon matched-pairs signed rank test, $p^{****}<0.0001$ for control, ns for ablated embryos).

5) Besides the concerns regarding the statistical analysis (see comment #1), this reviewer remains concerned that the SEM images to analyze changes in nuclear pore diameter are of insufficient quality to accurately determine the nuclear pore size to the extent to detect subtle differences of a few nm. While the authors have already made some changes to the text, the authors should either include TEM images to allow for more precise measurements (and conduct measurements by an observer blinded for condition) or better acknowledge this

limitation.
We now have toned down our conclusions:

1) We removed the reference to nuclear envelope stretching from the abstract (line 41).
2) We mostly comment on nuclear envelope integrity when discussing SEM results and keep any conclusions about nuclear pores speculative (lines 290-294, lines 510-511).
3) We add a sentence in the discussion (lines 516-520) acknowledging our limitiations and highlighting the need for future TEM investigations.
4) As we explained in the first response to reviewers' comments, we did indeed attempt TEM imaging. However, we were not able to unequivocally identify neural crest cells due to the metal grid used to mount TEM samples being superimposed to most of the tissue section, leading to significant loss of spatial information. By consulting with the EM experts in our EM imaging facility, we deemed lower EM resolution (10,000X SEM, the highest possible SEM magnification on our instrument) to be a more acceptable compromise compared to mistakenly attributing NC cell identity. We agree with the reviewer that future studies utilizing alternative mounting techniques (such as film-coated slot grids to prevent grid-bar occlusion) paired with high resolution TEM imaging will be required to validate these physical dimensions.

5) We now provide superplots for the SEM datasets (mean per embryo, see Figure 4I legend) and acknowledge our experimental limitations in main text (lines 516-520).

Minor comments:

6) Line 285. The authors write that migratory trunk neurocrest cells "show a transient loss of NE integrity". This statement seems to contradict their subsequent findings that these cells do not show nuclear envelope rupture, based on the nls-emGFP and BAF-mCherry experiments. The authors should rephrase this statement to avoid any confusion.

Thank you for spotting this. we have now edited to "transient nucleocytoplasmic leakage" to avoid confusion (line 273).

Reviewer #3 (Remarks to the Author):

The authors have provided substantial additional work, which has improved the manuscript and addressed most of the comments from all three reviewers.

My only remaining concern, unfortunately, is that it seems that LaminB2, which is still described as one of the main mechanisms for low DNA damage in confined NC migration, plays a rather modest role in protecting cells from DNA damage. While LaminB2 manipulations leads to changes in nuclei deformation, this does not correlate to DNA damages.

From new data provided by the authors, several mechanisms contribute to the low DNA damage. 1) Tissues surrounding migrating NCCs are rather soft. 2) LaminB2 modulates the dynamics of nuclei deformation and nuclei deformation is relatively transient. 3) Multipotent NCCs seems to have a strong protective program and have a up-regulated DNA repair machinery.

These results weakened the importance of Lamin2B regulation in DNA damage protection.

We thank the reviewer for this comment. We have now revised abstract (lines 45-46), introduction (lines 99-100),results (lines 462, 467) and discussion (lines 552,554-55) to better clarify our findings and softened conclusions (see Reviewer 2, point 2).
